# Leveraging breeding programs and genomic data in Norway spruce (*Picea abies* L. Karst) for GWAS analysis

**DOI:** 10.1186/s13059-021-02392-1

**Published:** 2021-06-13

**Authors:** Zhi-Qiang Chen, Yanjun Zan, Pascal Milesi, Linghua Zhou, Jun Chen, Lili Li, BinBin Cui, Shihui Niu, Johan Westin, Bo Karlsson, Maria Rosario García-Gil, Martin Lascoux, Harry X. Wu

**Affiliations:** 1 Umeå Plant Science Centre, Department Forest Genetics and Plant Physiology, Swedish University of Agricultural Sciences, SE-90183 Umeå, Sweden; 2 Program in Plant Ecology and Evolution, Department of Ecology and Genetics, Evolutionary Biology Centre and SciLifeLab, Uppsala University, Uppsala, Sweden; 3 College of Life Sciences, Zhejiang University, Zhejiang, 310058 Hangzhou China; 4 College of Biochemistry and Environmental Engineering, Baoding University, Baoding, 071000 Hebei China; 5 Beijing Advanced Innovation Centre for Tree Breeding by Molecular Design, Beijing Forestry University, Beijing, China; 6 Skogforsk, Box 3, SE-91821 Sävar, Sweden; 7 Unit for Field-Based Forest Research, Swedish University of Agricultural Sciences, SE-90183 Umeå, Sweden; 8 Skogforsk, Ekebo, 2250, SE-26890 Svalöv, Sweden; 9 CSIRO National Collection Research Australia, Black Mountain Laboratory, Canberra, ACT 2601 Australia

**Keywords:** Norway spruce, Frost damage, Genome-wide association study, Wood quality, Budburst stage, MAP3K gene

## Abstract

**Background:**

Genome-wide association studies (GWAS) identify loci underlying the variation of complex traits. One of the main limitations of GWAS is the availability of reliable phenotypic data, particularly for long-lived tree species. Although an extensive amount of phenotypic data already exists in breeding programs, accounting for its high heterogeneity is a great challenge. We combine spatial and factor-analytics analyses to standardize the heterogeneous data from 120 field experiments of 483,424 progenies of Norway spruce to implement the largest reported GWAS for trees using 134 605 SNPs from exome sequencing of 5056 parental trees.

**Results:**

We identify 55 novel quantitative trait loci (QTLs) that are associated with phenotypic variation. The largest number of QTLs is associated with the budburst stage, followed by diameter at breast height, wood quality, and frost damage. Two QTLs with the largest effect have a pleiotropic effect for budburst stage, frost damage, and diameter and are associated with MAP3K genes. Genotype data called from exome capture, recently developed SNP array and gene expression data indirectly support this discovery.

**Conclusion:**

Several important QTLs associated with growth and frost damage have been verified in several southern and northern progeny plantations, indicating that these loci can be used in QTL-assisted genomic selection. Our study also demonstrates that existing heterogeneous phenotypic data from breeding programs, collected over several decades, is an important source for GWAS and that such integration into GWAS should be a major area of inquiry in the future.

**Supplementary Information:**

The online version contains supplementary material available at 10.1186/s13059-021-02392-1.

## Background

Understanding how phenotypic traits are genetically controlled has been, and will likely remain for years to come, one of the central challenges in biology. Decades of quantitative trait loci (QTL) mapping and genome-wide association studies (GWAS) have detected a bounty of loci associated with the variation of quantitative traits in organisms ranging from humans to crops [1–3]. These studies have demonstrated that the identified loci only explained a small part of the variation in these traits. For instance, a recent study on human height, one of the most well studied quantitative traits, with ∼700 000 participants uncovered about 3000 near-independent associations explaining only ~25% height variation [4]. GWAS studies have also revealed substantial genotype-phenotype associations and the genetic architecture of complex traits in many crop species [5] including maize [6], rice [7], cotton, soybean, and other crops [8, 9]. By providing many insights into the architecture of complex traits and disease susceptibility, GWAS have facilitated advances in functional biology and gene editing [2, 10] and helped to increase the efficiency of genomic selection [11]. However, most GWAS vindicated Fisher’s infinitesimal model which assumes that most quantitative traits are controlled by a large number of loci of small effects and led to the development of new models of the genetic architecture of quantitative traits. Most notably, Pritchard and colleagues proposed an omnigenic model whereby traits are assumed to be controlled by core and peripheral genes: the core genes may be few and affect directly the trait and are themselves trans-regulated by a vast network of interacting regulatory peripheral genes [5, 12]. Pritchard and colleagues showed that the omnigenic model could explain the variation in complex diseases such as schizophrenia or rheumatoid arthritis or complex phenotypes such as height in humans [12]. All these results indicate that identifying loci underlying quantitative traits is intrinsically difficult and that there is therefore a strong incentive to leverage all available data. This is particularly true in forest trees, in general, and in conifers, in particular. Genotyping in conifers was challenged by their large and highly repetitive genomes [13] and phenotyping by their long-life cycle and heterogeneous living environment. Authors of previous studies made efforts to measure many phenotypic traits in a population of limited size to increase the statistical power. Long-term tree breeding programs usually accumulate a large amount of phenotypic data that are ideal to dissect the genetic basis of complex traits [14]. In the present study, we leveraged data available in the Swedish Norway spruce [*Picea abies* (L.) Karst.] breeding program where large sets of phenotype data were accumulated over the past 50 years from nation-wide field plantations, and genome-wide exomes capture polymorphism to identify genes involved in the variation of growth, phenology, and wood quality traits. Today, one of the main limitations of GWAS is the availability of phenotypic data or, in the cases when those data have been collected from existing breeding programs, their large heterogeneity. Coping with the heterogeneity of these data and using them efficiently is therefore a major challenge. However, success in doing so could unleash a large amount of information gathered over the years and partly help to overcome the dearth of phenotypic data.

Norway spruce is the most economically important conifer species in Europe [15], especially for Nordic countries, and a large Swedish breeding program was established in the middle of last century from seeds collected from elite trees selected across the country [16]. By elite trees we refer to trees that were selected based on their outstanding phenotype or breeding values to constitute the base population of the breeding program. Many of the selected elite trees turned out to be recent introductions from other European countries, and hence, the current Swedish Norway spruce breeding population is representative of most of the species range, at least in its southern part [17]. In contrast, the northern part of the breeding program is genetically more homogeneous (Li et al. 2020, in preparation). A strong population structure needs to be accounted for when doing the association per se, and it also needs to be accounted for when gathering phenotypic data. Indeed, the available phenotypic data on the elite trees are from two sources. In the easiest case, the elite trees were planted in common gardens and highly heritable traits, such as wood quality or budburst, directly measured on the elite trees themselves. However, for lower heritability growth traits, such as height or diameter, direct measurement on elite trees is less reliable and instead, breeding values of the elite trees could be estimated from measurements on their half/full-sib offspring in progeny tests. These progeny tests usually varied in composition, were established in different climatic regions, and were measured at different ages, thereby introducing an added layer of heterogeneity and complexity that needs to be accounted for before the phenotypic data are used for association analysis.

In this study, we conducted a GWAS for budburst stage (BB), frost damage (FD), tree height (Height), diameter at breast height (DBH), and wood quality traits in Norway spruce using data from the Swedish long-term breeding program (Table 1). These highly heterogeneous data were height measured in progeny tests across the whole Sweden, DBH, only in progeny tests from the southern and central Sweden, FD in two large progeny tests, and BB and wood quality traits were measured directly on elite trees in three common gardens. Various steps were devised to account for the heterogeneity of the data. The main aim of the study was to leverage existing data from the Swedish Norway spruce breeding program to identify single-nucleotide polymorphisms (SNPs) associated with the variation in phenotypic traits. For a subset of traits, we also validated the identified SNPs using related populations and RNA-Seq experiments.

## Results

### Genotyping the elite Swedish breeding materials using exome capture sequencing

**Fig. 1:**
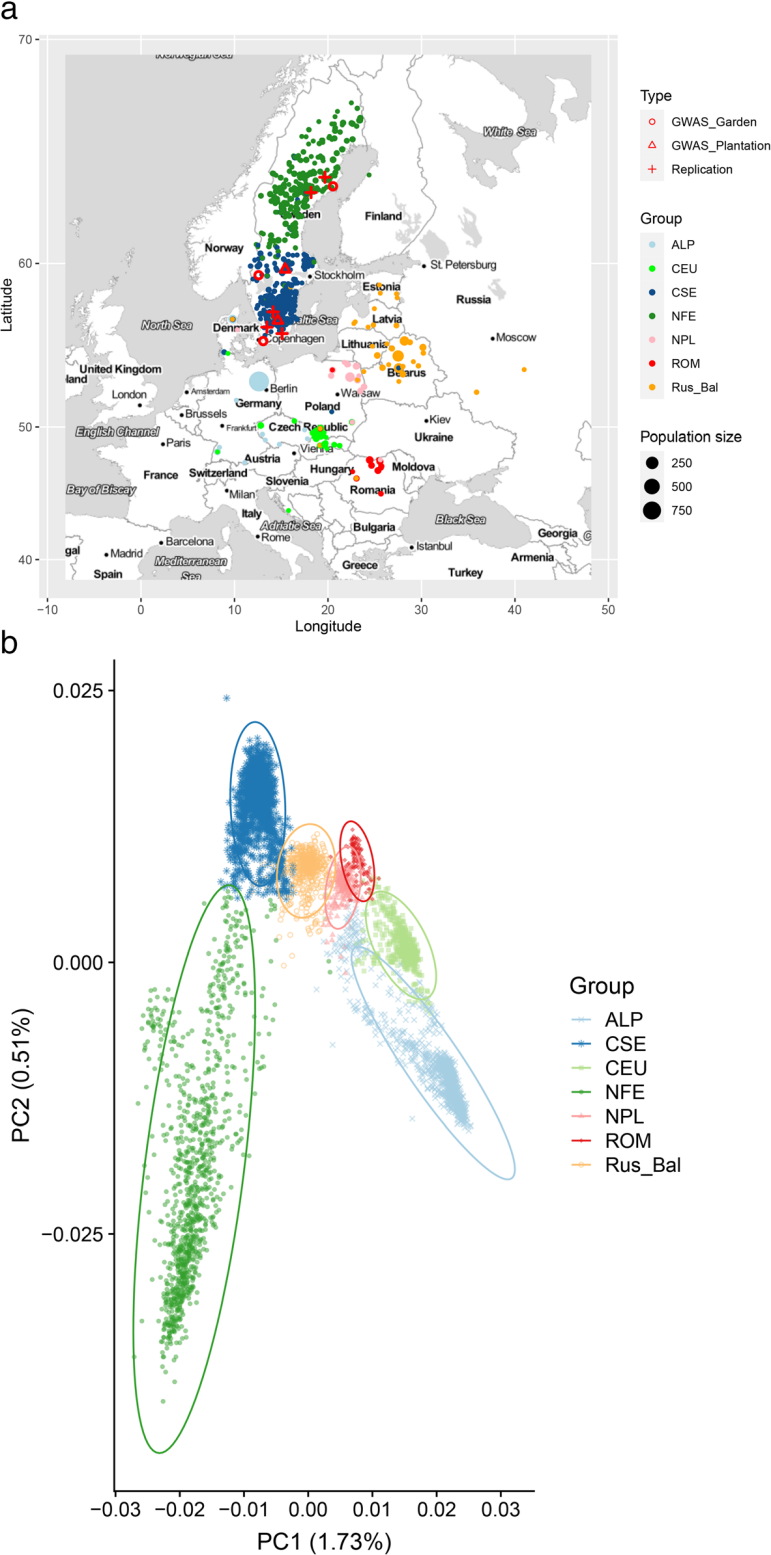
Geographic location and population structure of 5056 elite trees selected for the Swedish breeding program. **a** Map (top) of geographic location of plus trees using the known original location for 2779 trees and predicted location of 2277 trees and **b** PCA results (bottom) of all 5056 individuals colored by cluster groups. For the group without original location information, we used predicted group information; thus, the size of the dot represents the number of individuals in those groups. The different genetic clusters (Carpathian, red, ROM; Alpine, light blue, ALP; Central Europe, green, CEU; Northern Poland, light plink, NPL; Russia-Baltic, orange, Rus-Bal; central and southern Sweden, dodger blue, CSE; Fennoscandia, forest green, NFE). The detail of the number of seven groups was shown in Additional file 1: Table S1. The materials (type) used for GWAS were located in three common gardens (red circles), in which threes wood quality traits and budburst stage (BB) were measured directly, two field half-sib plantations (red triangles), in which FD were scored directly, and the materials used for SNP replication/validation were located in two full-sib progeny field plantations in northern Sweden and three full-sib progeny field plantations in southern Sweden (red plus). The field plantations with growth traits are shown in Additional file 2: Figure S12

Using exome sequencing with customized probes [18], 10.6 M SNPs were identified after initial SNP calling using GATK HaplotypeCaller, out of which 354 401 SNPs passed initial quality control (see the “Materials and methods” section). In the 5056 unrelated elite trees, 299 240 SNPs were found to be in Hardy-Weinberg equilibrium (*P*<1.4 × 10^-7^). These SNPs were distributed across 24,104 contigs (*P. abies* v1.0 annotation) [19]. The mean accuracy of the imputed genotypes was estimated to be 0.97 at both individual and SNP level (Additional file 2: Figure S1). After filtering of SNPs with a low allele frequency (MAF < 0.03), 134 605 SNPs were kept for downstream analysis. The first two principal components explained 1.46% and 0.46% variance of the marker matrix (Additional file 2: Figure S2). As has been reported in [17], PCA detected seven clearly delineated clusters across the native range of the species (Fig. 1b). The seven genetic clusters were Alpine (ALP, light blue), Carpathian (ROM, red), central Europe (CEU, green), Northern Poland (NPL, pink), Russia Baltic (Rus-Bal, orange), Central and southern Sweden (CSE, blue), and Fennoscandia (Northern Sweden, Finland and Norway) (NFE, forest green) (Additional file 1: Table S1 and Fig. 1b). Importantly, Sweden is divided into two main clusters. Out of the 5056 elite trees, 2277 trees had no documented origin. PCA was conducted using the genotype matrix of the 5056 elite trees, and then, a random forest method was used to assign these 2277 trees to one of the seven genetic clusters. 966 trees were assigned to the ALP, 394 to CSE, 154 to Rus-Bal, 91 to NPL, 67 to CEU, 581 to NFE, and 24 to ROM (Additional file 1:Table S1 and S2).

### Genetic variation across different genetic clusters and genetic parameters of phenology, growth, and wood quality traits

**Fig. 2:**
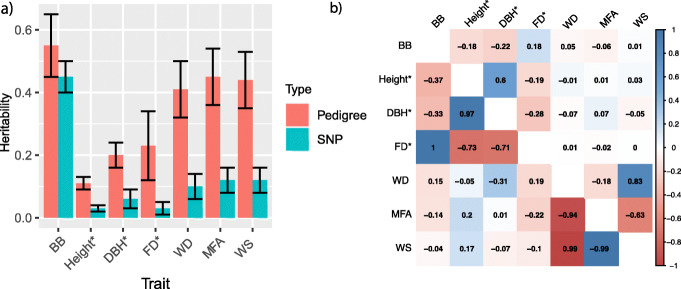
Heritabilities and genetic correlations among traits. **a** Pedigree-based and SNP-based narrow-sense heritability of the seven traits, with black line representing the ± standard errors. **b** Pair-wise Pearson product-moment correlations (upper diagonal) and genetic correlations (lower diagonal) among de-regressed estimated breeding values (dEBVs) of tree height (height), diameter at breast height (DBH), and frost damage (FD), adjusted phenotypic values of budburst stage (BB), wood density (WD), microfibril angle (MFA), and wood stiffness (WS). The color spectrum, bright blue to bright red presents highly positive to highly negative correlations, and the number represents the correlation values. ^*^The SNP heritability only explained the proportion of dEBVs variance explained by SNP-based genomic relationships, and dEBVs of the three traits were used to estimate the phenotypic and genetic correlations in b

In general, de-regressed estimated breeding values (dEBVs) or adjusted phenotypic values of the seven traits showed an approximately normal distribution (Additional file 2: Figure S3). All seven traits showed significant differentiation among genetic clusters (*p*<10^-6^, Additional file 2: Figure S4) although it should be pointed out that some traits are represented by very few individuals in some clusters (e.g., DBH for NFE and most traits for ROM). Growth and phenology traits displayed a significant correlation with latitude and BB, a strong cline with longitude (Additional file 2: Figure S5). Wood quality traits did not show any trend for both latitude and longitude. In the three common gardens, WD, MFA, and WS exhibited moderate to high repeatability ranging from 0.36 to 0.60 (Additional file 1: Table S3) for parental elite tree population. *F*_st_ among seven genetic clusters are shown in Table S4. The NFE and ALP domains were the most divergent (*F*st =0.026), and the ROM domain was closest to NPL. The CSE cluster was much closer to the Rus-Bal cluster than to NFE. The pedigree-based narrow-sense heritabilities of these traits estimated from two full-sib progeny tests were 0.41, 0.45, and 0.44, respectively (Fig. 2). However, SNP heritabilities of WD, MFA, and WS estimated from elite tree population were only 0.10, 0.12, and 0.12, respectively. In the northern Sävar common garden, BB displayed the highest estimated repeatability (0.81). For the same trait, a pedigree-based heritability of 0.55 was obtained from the two full-sib progeny plantations, and SNP heritability was 0.45 from the three common gardens (Additional file 1: Table S3, Fig. 2). For height and DBH, the direct measurement from those common gardens may not be reliable due to the stand management including topping and thinning. Thus, the repeatability of the two growth traits in those common gardens was not estimated. FD was only scored in two large half-sib progeny plantations; therefore, the narrow-sense heritabilities of height, DBH, and FD estimated from the two plantations were shown in Fig. 2 and were 0.11, 0.20, and 0.23, respectively. “SNP heritabilities,” the proportions explained by the genomic relationship matrix (GRM), were 0.25, 0.30, and 0.15, respectively.

Negative genetic correlations between FD and height and between FD and DBH varied from −0.54 to −0.84 in two southern Swedish progeny plantations (Additional file 1: Table S5) and from −0.71 to −0.73 for the whole elite tree population (Fig. 2b), referred to subsequently as the whole population. Significant negative Pearson’s correlation coefficients were observed between adjusted phenotypic BB and both height (−0.18) and DBH (−0.22) for the whole population (Fig. 2b). More strongly negative genetic correlations between BB and both height (−0.37) and DBH (−0.33) were observed from the whole population using GRM. Generally, low correlations between growth traits and wood traits were observed from the whole population (Fig. 2b). For example, genetic correlations between height and wood quality traits varied from -0.05 to 0.20 for the whole population (Fig. 2b). However, a strong positive correlation between height and Pilodyn penetration (inverse of WD) was also observed from two full-sib progeny plantations (Additional file 1: Table S5).

### Mapping alleles underlying the variation of the seven traits

There were strong population genetic structure and phenotypic differences between genetic clusters, especially between NFE (Northern Sweden, Finland, and Norway) and other genetic clusters. The phenotypic values of growth and phenology traits vary clinally and at least three of the seven genetic clusters have enough data for performing GWAS with more than 800 individuals each (Table 1). Thus, we performed GWAS for the whole population and each cluster. To control for false-positive detection risk, remove the confounding between testing markers and kinship, and reduce the computational load, we used a recently developed model, Bayesian Information and Linkage-disequilibrium Iteratively Nested Keyway (BLINK; implemented in the BLINK R package [21]. However, this method will not detect the candidate QTNs that are in strong LD with QTN that have smaller *p* value SNPs. To detect all pseudo QTNs and make results comparable across different genetic clusters, we also used a compression mixed linear model (CMLM) [22] implemented in the Gapit R package [23]. One of the main aims of both BLINK and CMLM is to consider cryptic relationships among individuals and control for false-positive detection risk. Thus, here we only needed to consider the effect of population structure for GWAS. The QQ plots and details of the associated SNPs are described in Additional file 2: Figure S6 and Additional file 1: Table S6. At 5% FDR, a total of 64 unique SNPs were detected by the two methods using the whole population or different genetic clusters for the seven traits (Additional file 1: Table S6, S7 and S8). The LD decayed (*r*^2^) rapidly to 0.2 within 37 base pairs (bps) for contigs harboring significant SNPs (Additional file 2: Figure S7). After grouping SNPs in tight LD (*r*^2^ >0.2) with each other, the number of independent association signals (SNPs) for seven traits was 55 (Additional file 1: Table S7). The number of independent associations varied between 1 (MFA) and 40 **(**BB) (Additional file 1: Table S8). Except for BB, the number of detected significant and independent associations was generally small, height (*n*=3), DBH (*n*=4), FD (*n*=3), WD (*n*=4), MFA (*n*=1), and WS (*n*=4).

**Table 1:** Number of trees genotyped and used in analyses for population structure and GWAS

Genetic cluster used for GWAS	Genotyped^a^	Trait						
		BB	Height	DBH	WD	MFA	WS	FD
ALP	1125	1010	929	877	1005	1006	1005	474
CSE	1571	1501	1418	1146	1504	1508	1501	623
CEU	508	350	301	280	350	350	350	143
NFE	1041	832	824	30	822	821	820	5
NPL	197	189	178	176	190	171	189	78
ROM	124	30	36	28	29	29	29	17
RUS_Bal	490	226	152	137	227	227	227	88
Total no. of genotypes used for GWAS	5056	4138	3838	2674	4127	4112	4121	1428
*GWAS validation* of tree height, BB, and wood quality traits	1370	1067	1370		1370	1370	1370	
*GWAS validation of BB*	914^b^	914^b^						
Grand total	7340	6119	5208	2674	5497	5482	5491	1428

^a^The number of genotyped individuals in each genetic cluster using for population structure

*BB* budburst stage, *Height* tree height, *DBH* diameter at breast height, *FD* frost damage, *WD* wood density, *MFA* microfibril angle, *WS* wood stiffness

The different genetic clusters are Alpine, ALP; central and southern Sweden, CSE; Central Europe, CEU; Fennoscandia, NFE; Northern Poland, NPL; Carpathian, ROM; and Russia-Baltic, Rus-Bal

^b^The 914 individuals were genotyped by a recently developed *Picea abies* SNP array [20]

### SNPs associated with phenology and wood quality traits

**Fig. 3:**
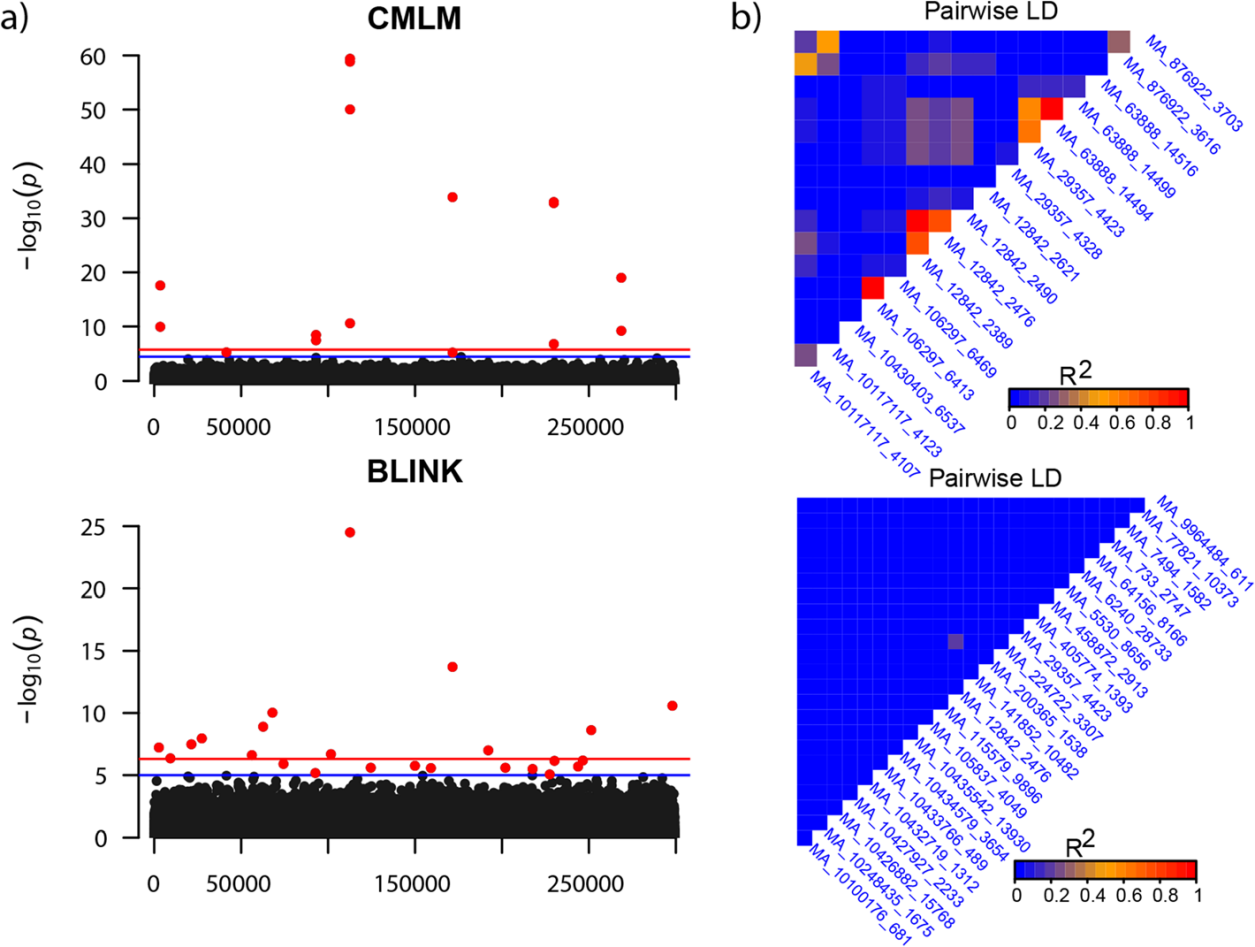
Manhattan plots and linkage disequilibrium (LD) for loci associated with budburst stage (BB). **a** Two Manhattan plots showing significant association (red dots) with BB using CMLM and BLINK in the whole Swedish breeding population, and **b** LD between single nucleotide polymorphisms (SNPs) associated with BB in CMLM and BLINK. Blue and red horizontal lines indicate a threshold of *log*_10_(1*e*^−05^) and *log*_10_(1*e*^−07^), respectively

For BB, we identified 40 independent associations, 31 from BLINK, and 11 from CMLM, with two of them (MA_1284_2490, MA_29357_4423) overlapping (Additional file 1: Table S9). Most of the significant associations (32 SNPs) were detected in the whole population (*n*=4138), while one SNP (MA_798143_7416) was only detected by CMLM in the CEU cluster, and seven associations identified by BLINK were specific to some of the genetic clusters: CEU (*n*=1), CSE (*n*=1), and NFE (*n*=5). More associations were identified by BLINK (*n*=24) than by CMLM (*n*=10) in the whole population as shown in Manhattan plots and pair-wise LD *r*^2^ in Fig. 3. For FD, we only identified three independent associations in the whole population (*n*=1428). Only one association (MA_12842_2490) overlapped between BLINK and CMLM.

For WD, we detected four association signals in the whole population and two in the ALP cluster (Additional file 1: Table S9). Two association signals (MA_10432695_11851 and MA_93306_19178) were detected by both BLINK and CMLM in the whole population. Two independent SNPs in the ALP cluster were only detected by BLINK. For MFA, we only identified two independent associations in cluster NFE from BLINK. For WS, we identified four independent associations from BLINK and three from CMLM (Additional file 1: Table S9) in two clusters (CEU, *n*=1; Rus_Bal, *n*=3). Only SNP MA_93306_19178 is unique to BLINK. Four SNPs detected from CMLM completely overlapped with those detected from BLINK, resulting in four associations in total.

### SNPs associated with growth traits

For height, we only detected three associations with BLINK in the whole population (Additional file 1: Table S9). For DBH, we identified five SNPs in the whole population, with two and three SNPs detected by CMLM and BLINK, respectively, with no overlap.

### SNPs jointly associated with multiple traits (BB, FD, and DBH)

Two independent SNPs (MA_12842_2389/2490 and MA_29357_4423) were found to be associated with three traits (BB, FD, and DBH) simultaneously (Additional file 1: Table S9) while one independent SNP (MA_63888_14494) was significantly associated with two traits (BB and FD).

In general, SNPs associated with phenology traits BB and FD explained a larger proportion of the total phenotypic variation (*R*^2^) in the whole population (Table 2 and Additional file 1: Table S10). The independent SNP MA_12842_2490 explained the largest variation of 6.7% for BB in the whole population (Additional file 1: Table S6). Similarly, the independent SNP MA_12842_2490 explained 5.2% and 1.5% variation for FD and DBH in the whole population. We also observed that SNP effect size were the largest for SNPs with MAF from 4 to 25% for BB (Additional file 2: Figure S8). For height, BLINK detected three significant SNPs in the whole population only with PVE ranging from 0.3 to 0.5%. For BB, all significant SNPs accounted for a total of 15.7% phenotypic variation, while for FD, DBH, and height, all significant SNPs accounted for a total of 4.9%, 2.6%, and 1.5% additive genetic variation in the whole population, respectively (Table 2).

**Table 2:** Summary of the number of independent SNPs/number of SNPs associated with the seven traits

Method	Genetic cluster	Trait						
		BB	Height	DBH	FD	WD	MFA	WS
*CMLM*	*Whole pop.*	*10/16 (0.68–6.70)*	*0*	*2/3 (1.15–1.51)*	*3/6 (2.22–5.19)*	*3/3 (0.68–0.82)*	*0*	*0*
	*ALP*	*7/12*	*0*	*0*	*NA*	*0*	*0*	*0*
	*CEU*	*2/5*	*0*	*0*	*NA*	*0*	*0*	*1/2*
	*CSE*	*2/4*	*0*	*0*	*NA*	*0*	*0*	*0*
	*NPL*	*2/4*	*0*	*0*	*NA*	0	0	*0*
	*Rus_Bal*	*0*	*0*	*0*	*NA*	0	0	*2/2*
	*ROM*	*NA*	*NA*	*NA*	*NA*	*NA*	*NA*	*NA*
	*NFE*	*0*	*0*	NA	*NA*	0	0	0
*BLINK*	*Whole pop.*	*24/24 (0.13–6.70)*	3/3 *(0.33–0.48)*	*2/2 (0.57–0.62)*	*1/1 (5.19)*	*3/3 (0.07–0.82)*	*0*	*0*
	*ALP*	*1/1*	0	*0*	*NA*	*2/2*	*0*	*0*
	*CEU*	*2/2*	0	*0*	*NA*	*0*	*0*	*1/2*
	*CSE*	*2/2*	0	*0*	*NA*	*0*	*0*	*0*
	*NPL*	*1/1*	0	*0*	*NA*	*0*	*0*	*0*
	*Rus_Bal*	*0*	0	*0*	*NA*	*0*	*0*	*3/3*
	*ROM*	*NA*	NA	*NA*	*NA*	*NA*	*NA*	*NA*
	*NFE*	*6/6*	0	*NA*	*NA*	*0*	*1/2*	*0*
	*Total SNPs*	*40/47 (0.13–6.70)*	3/3 (0.33*–*0.48)	*4/5 (0.57–1.51)*	*3/6 (2.22–5.19)*	*6/6 (0.07–0.82)*	*1/2 (0.14)*	*4/5 (0.06–0.21)*
	*R*^2^*	*15.7*	*1.5*	*2.6*	*4.9*	*2.4*	*0.1*	*0.5*
	*R*^2^**	*4.7*	*1.7*	*NA*	*NA*	*0*	*0*	*0*

In this study, we performed GWAS in the whole elite tree population and seven genetic clusters by two methods (CMLM and BLINK), phenotypic variance explained (PVE^2^) by a single SNP shown in parenthesis, and cumulative phenotypic variance explained by all associated SNPs (*R*^2*^) for each trait in the whole elite tree population and the validation population (*R*^2^**) in the two full-sib progeny field plantations in Northern Sweden. *BB* budburst stage, *DBH* diameter at breast height, *FD* frost damage, *WD* wood density, *MFA* microfibril angle, *WS* wood stiffness. NA: represents that we did not perform CMLM for each genetic cluster because of small cluster size in several clusters, such as NFE and ROM in Table 1

### Replication of associated SNPs using data from several full-sib progeny plantations

All SNPs associated with five of the seven traits (except for FD and DBH, due to lack of additional phenotypic values) were evaluated using two full-sib progeny plantations established in northern Sweden (Additional file 1: Table S6). Out of the 55 reported association signals, 52 signals could be tested, of which ten (seven for BB, two for height, and one for WD) were replicated at nominal significance (*P* value <0.05) (Additional file 1: Table S8). Interestingly, two independent SNPs (MA_12842_2389 (_2490) and MA_29357_4423) that were associated with BB, FD, and DBH and one independent SNP (MA_63888_14494 (_14499)) that was associated with BB and FD were also found associated with BB in the validated population (Additional file 1: Table S9).

Using our recently developed 50K SNP array developed from the whole genome sequencing data [20], we found that two SNPs in contig MA_12842 were in the SNP array. The novel SNP MA_12842_2774 was associated with BB (*P* value, 1.15E−06), five (33%) of the total 12 SNPs associated were located outside of the gene regions (Table S11), comparable with 49% SNPs selected outside of the gene regions in the 50K SNPs array.

### Identification of candidate genes underlying the associated SNPs

In total, there were 43 annotated genes located on 37 contigs underlying SNPs significantly associated with 40 independent SNPs for BB (Additional file 1: Table S12). Among these genes, 18, 24, and 21 candidate genes showed differential expression between early and late burst buds, among different sampling times, and different temperatures at sampling time, respectively (Additional file 1: Table S13 and Additional file 2: Figure S9).

A total of five candidate genes were identified on the contigs holding SNPs associated with FD (Additional file 1: Table S12). The four putative MAP3K15 genes (MA_12842g0010, MA_12842g0020, MA_12842g0030, and MA_29357g0010) detected for FD were also detected as candidate genes for BB and DBH. However, only the candidate gene MA_12842g0030 showed an expression difference with temperature in five sampling times (Table S13).

We identified 12 SNPs, located in 10 contigs harboring ten genes, associated with the wood quality traits including WD, MFA, and WS (Additional file 1: Table S7 and S12). Those genes are expressed in different tissues (Additional file 1: Table S14). A SNP, MA_10435921_15994, located in the intron of gene MA_10435921g0010 was associated with MFA in the cluster NFE. MA_10435921g0010 was annotated to code an ENTH/ANTH/VHS superfamily protein associated with phospholipid binding in *Arabidopsis thaliana*. A SNP located in the intron of gene MA_40805g0010 (a homolog of gene SIK 1 in *A. thaliana*) detected in cluster ALP for WD encodes serine/threonine kinase and is involved in the regulation of cell population proliferation and cell expansion to maintain organ growth [24].

For height, SNPs MA_10432234_28084 and MA_222324_1075 were located in an intron and upstream of the genes and the third SNP, MA_40506_4499, was located in an intergenic region (Additional file 1: Table S6 and S9). The two genes (MA_10432234g0010 and MA_222324g0010) are expressed in different tissues (Additional file 1: Table S14). The SNP MA_10432234_28084 located in the intron of the candidate gene MA_10432234 is annotated as a homolog of *A. thaliana* Golgi Nucleotide sugar transporter 3 (GONST3) and explained 0.56% of the total genetic variance (Additional file 1: Table S6 and S12). For DBH, GWAS identified five SNPs located within four contigs, harboring six genes (Additional file 1: Table S8 and S12). The SNP MA_10428754_23026 located in the splice region of the candidate gene MA_10428754g0010, annotated as a homolog of a lipid transporter in *A. thaliana*, explained 0.62% of total genetic variance.

**Fig. 4:**
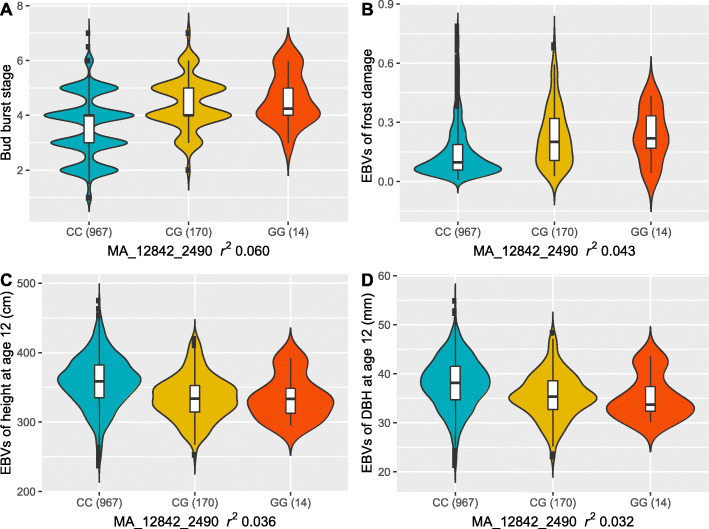
Violin plots of pleiotropic locus effects. Effect of pleiotropic QTL (MA_12842_2490) of budburst stage and frost damage on **A** adjusted phenotypic budburst stage, in which higher values means budburst earlier, **B** estimated breeding values (EBVs) of frost damage at age 7, in which higher value means that bud was damaged more severely, **C** EBVs of tree height at age 12, and **D** EBVs of tree diameter at age 12 in the southern Swedish field plantation (F1150). CC, CG, and GG are genotypes of SNP MA_12842_2490. The value in parenthesis is the number of individuals for that genotype

The two tightly linked SNPs (MA_12842_2389 and MA_12842_2490), located on the same contig MA_12842, were simultaneously associated with BB, FD, and DBH forming a single independent signal (Additional file 1: Table S6, S7, and S8), indicating a potentially shared genetic component for the three traits. This signal alone (6.7% of PVE for BB) in the whole population also accounted for 5.2% of PVE for FD at the progeny population (F1150) suggests a major gene for BB and FD (Fig. 4). The genotype GG has a mean FD of 0.23 and an increase of 31.9% compared with homozygosity of CC (0.15). In contrast, height and DBH at age 12 for genotype GG decreased by 6.3% and 7.4%, respectively.

**Fig. 5:**
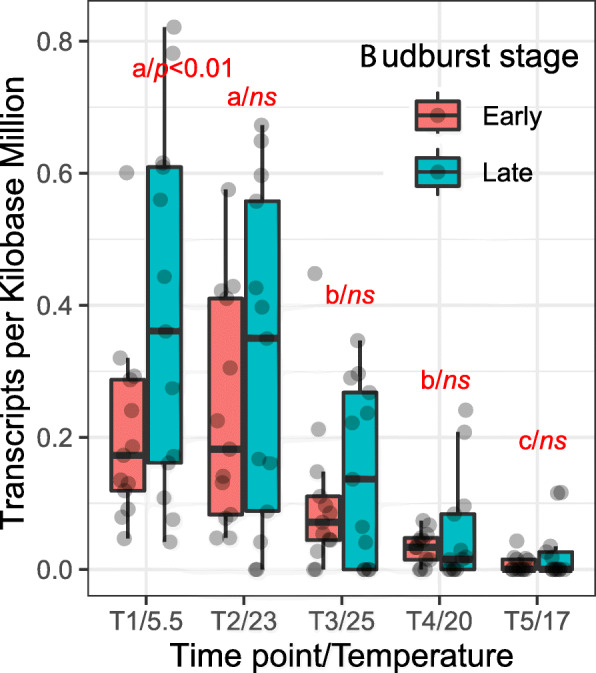
Difference in gene expression during budburst stages. The expression of RNA-Seq in transcripts per kilobase million (TPM) for gene MA_12842g0030 (MAP3K) from six early bud burst and six late bud burst clones, sampled five times in May 2018. The x-axis is the five sampling time points (T1–T5) and their corresponding temperature of sampling time (°C). Ns represents non-significance between early and late budburst clones at the same sampling time, and different letters (a, b, and c) represent the significant difference between different sampling times

The contig MA_12842 with a missense SNP MA_12842_2490 harbored three candidate genes (MA_12842g0010, MA_12842g0020, and MA_12842g0030) (Additional file 1: Table S12). We also found that only the candidate gene MA_12842g0030 showed significantly different expression between early and late budburst trees at the first sampling time with the lowest recording temperature (5.5°C) during budburst in spring (Fig. 5). The expression level of candidate MA_12842g0030 decreased from the first two sampling times (May 3, 2018) to the last (May 27, 2018) for both early and late budburst samples (Fig. 5). The developmental response and differential expression between early and late BB samples highlighted MA_12842g0030, annotated as putative mitogen-activated protein kinase kinase kinase 15 (MAPKKK15/MAP3K15), to be a promising candidate gene for BB and FD.

## Discussion

As genomic data are today much easier to obtain, the main limitation of GWAS has become the availability of accurate phenotypic data. Breeding programs are one of the main sources of phenotypic data even though those tend to be limited to traits related to production, have generally be accumulated over long periods from numerous experimental plantations. However, such extensive data is not without its challenges, such as its high heterogeneity. In the present study, we have attempted to use data originated from the Swedish breeding program for Norway spruce to carry out one of the largest scales GWAS for growth, phenology, and wood quality traits in woody plants. Below, we discuss the potential and limitations of this approach and what can be done to remedy the latter as well as some of the specific results of the present GWAS. It is important to note that in the present study our main aim was to identify highly significant and reproducible QTLs with large effect. This remains one of the main goals of GWAS, especially in applied fields, although, given the highly polymorphic nature of quantitative traits, it may also be reasonable to be more liberal and focus instead of the distribution of effects, independently of the size of those (e.g., in forest trees [14, 25])

### Factors affecting the power of GWAS

Several factors affect the detection of loci controlling trait variation: sample size, minor allele frequency, size of allelic effect, markers density, heritability, LD structure, and population structure [26, 27]. The sample size required for detecting effective loci is affected by the number and effect size of underlying QTLs, heritability, statistical power, and significance level used. In typical forest tree species, assuming an ideal population without population structure where all causal variants have been genotyped, detecting QTLs with 0.5 allele frequency that account for 12.0%, 6.6%, and 4.3% of the variation of the trait would require a population size of approximately 300, 550, and 850 trees, respectively. Such sample sizes were derived with further assumption of an 80% power and a significance level (type I error rate) of 1×10^-7^ (Additional file 2: Figure S10, [26]). Of course, no such population exists and the required population size will typically be much larger.

#### Sample size

Here, we used more than 4000 genotypes (except for height of 3838, DBH of 2674, and FD of 1428 trees, Table 1) to detect association for the seven quantitative traits and 1370–1981 genotypes for verification of these traits. Despite this large sample size, the statistical power was curtailed by population structure and the heterogeneity of the data and this apparently large sample size is still not sufficient to detect loci that explained less than 0.9% of the variation, as suggested by the simulations in Hall et al. [26]. Our data, however, show that the statistical approach used could make a difference. For example, using the CMLM method, we did not detect any loci with PEV < 0.68% (Additional file 1: Table S10) in each specific genetic cluster. The skewness in allele frequency, low genotyping density, and cost in correcting for confounding by population structure likely mean that an even larger sample size would be required to detect additional loci with lower effect size. However, using the recently developed multi-loci BLINK method, one could detect loci explaining less than 0.9% of the total variation when more than 2674 genotypes were available (e.g., DBH). The fact that the number of SNPs detected for most traits including growth traits, BB, and FD in the whole population was much more than the number of SNPs associated in genetic clusters clearly indicate that sample size matters and is probably the most important factor in detecting QTLs (Table S10). For example, 32 and 0–8 SNPs were detected for BB in the whole population and seven clusters, respectively. Our results are similar to those obtained in human GWAS where the number of discovered variants is strongly correlated with experimental sample size [3]. In human, sample sizes of over 1 million individuals are now becoming a reality for some traits, but in trees, genotyping and phenotyping of more than 10,000 trees are still a challenging task due to the costs and availability of reliable phenotypic data. Advances in statistical methods, e.g., multi-locus or multivariate models would be one possible option to improve the statistical power without increasing the sample size too much [28]. Reducing the stringency of statistical thresholds and using complementary approaches such as RNA-Seq or qPCR would be another option [29]. However, as illustrated by GWAS in humans, limits to statistical power are likely rooted in the nature and architecture of quantitative traits and there will be inherent limits to the detection of SNPs associated with quantitative traits.

#### Minor allele frequency

In GWAS, the detection of true low-frequency alleles associated with complex traits is also challenging as it also implies that large and genetically diverse populations are required [26]. Most of GWAS in tree species (particularly conifers) have employed only a few hundred individuals to identify loci potentially linked to phenotypic traits [13, 26, 28, 30–32]. In the present study with a relatively large GWAS population and high genotype quality, we used variants with minor allele frequencies as low as 0.03, instead of 0.05 used in most of the GWAS in plant species. Since rare alleles play an important role in both genetic regulations of traits in plant species [13, 33], this matters. One of the hypotheses attributed to the missing heritability is the substantial amount of quantitative variation linked to the cumulative effect of rare alleles that may not be detected in GWAS using a small sample size [34]. The predominance of alleles of small effects could simply be due to the fact that mutations with large effects are purged or kept at low frequency due to their deleterious effects [13, 35].

#### Number and nature of markers and heritability

To the best of our knowledge, we used the largest population in tree species for GWAS, but only a few SNPs were detected for all traits, except for BB. There could be many reasons for this. Firstly, exome captures only cover a small fraction of the coding region (0.4% of the whole genome). In annual plants, however, a clear pattern has emerged indicating that the association signal of common variants in large sample sizes, although spread across the entire genome, is heavily concentrated in regulatory DNA in open chromatin marked by deoxyribonuclease hypersensitive sites [36]. This could also explain the missing heritability. By analyzing pedigree-based and SNP-based heritability, both heritabilities are comparable for BB, which indicates most of the causal variation for BB may be located in the exome region, but not for wood quality traits, in which SNP-based relationships only explained 10 to 12% of phenotypic variance. For growth traits, we used dEBVs instead of phenotypic values for GWAS. The model using GRM only explained 11 to 23% of dEBVs and only a few SNPs were detected, indicating growth traits are more polygenic, in line with previous results [14]. Wood quality traits in Norway spruce are also considered to be highly polygenic traits [37]. SNP heritability only explained a small proportion of pedigree heritability, and therefore, only a few SNPs or even no SNPs were detected in our GWAS even though we used more than 4000 samples. As for other traits, many causal variants for wood quality traits may be located outside the exome region. This will be consistent with an omnigenic model of inheritance of quantitative traits [5, 12].

Genotyping in the two most commercially important angiosperm tree species (poplar and Eucalypts) is relatively well developed due to their small and high-quality reference genomes (400–600 Mb) [38–41]. However, only draft versions of the genome are available in a few important conifer species [19, 42, 43]. To obtain markers covering the whole genome, one solution is to develop a genome-wide high-density SNP array [44, 45]. Recently, a large consortium organized construction of six 50K SNP arrays for Norway spruce, loblolly pine (*Pinus taeda* L.) and radiata pine (*Pinus radiata* D. Don.) [20, 46]. This will facilitate GWAS studies in the near future but is, however, unlikely to be a game-changer since SNP-arrays usually do not capture well rare variants even using imputation based on reference genomes [47]. Another alternative solution to increase the marker density would be to perform whole-genome re-sequencing using a low coverage and impute the missing genotypes to re-sequencing density, but this would require better genome assembly and reference haplotype panel than those currently available.

#### Population genetic structure and structure of the phenotypic data

Two aspects that affect the power of a GWAS are the overall genetic structure of the studied population and the genetic relationships among the tests from which the phenotypic data originate. Ideally, GWAS are conducted on a set of unrelated individuals that have been accurately phenotyped. In this respect, progeny field plantations in tree species, particularly in conifers, are suitable because progenies are at the same age and environmental variation is minimized through a proper field experimental design [48]. Additionally, accurate phenotypic data have often been collected over several years in the existing breeding programs. However, progeny plantations usually consist of only 100 to 400, rarely more than 1000, half-sib or full-sib families [49, 50]. The result is that access to a large number of unrelated trees in the same environment, associated with an existing breeding program, is difficult. One can, of course, gather data from many progeny tests, but then, as in the present study, will be faced with the fact that trees are grown in different environments and are not planted in the same year. In addition, different sets of genetic material are planted at different sites with minimal or no genetic linkage between sites. This creates a considerable challenge to link the different progeny tests. Provenance plantations (common gardens) may provide a larger number of unrelated trees, but samples from provenance plantations are often structured. Clonal common gardens of elite trees are also an option. However, they have their limitations since trees are often grafted and possible interaction between the rootstock and the scion can make this type of plantation unsuitable, especially for low heritability traits such as growth. Scion-rootstock interactions may be less influential for highly heritable traits such as wood quality and phenology traits [51]. Clonal common gardens, however, also offer advantages, the trees are usually unrelated, are represented by several ramets (i.e., copies of the same genotype), and numbers are in the range from several hundred to thousands of trees. Another advantage of clonal common gardens is that their phenotypes are often available as ranks (estimated breeding values, EBVs) predicted from progeny plantations through multiple generations, ages, and sites. Recently advances in the development of factor-analytic and spatial analyses offer the possibility to obtain accurate breeding estimates in such meta-data analyses [52]. We were able to remove the site, age, and spatial environmental variation and to overcome the minimum genetic connection issue among many sites for the 120 field plantations in this project. By implementing the factor-analytic and spatial analyses, more accurate phenotypic values from such large breeding program were derived for the GWAS with significant genes detected for phenology and wood traits. However, to further improve the accuracy of phenotype for GWAS, future plantings should include greater genetic linkage, particularly between southern and northern Swedish plantations (Additional file 1: Table S15). The disconnection between the southern and northern plantations also motivated us to perform GWAS within each cluster. Thus, we would recommend establishing several large plantations in southern, central, and northern Sweden with a sufficient number of common parents/elite trees, i.e., at least 10–20% common parents [49].

#### Population structure

The present analysis of population structure showed a strong systematic stratification (Fig. 1), which is in agreement with previous studies using a subset of the data. BB showed a strong cline with longitude and latitude [14]. As expected, we did not find that wood quality trait showed a cline. Growth traits only showed a moderate cline with latitude as reported in Milesi et al. (2019). This difference in the structure of the traits has important implications. BB, in contrast to growth traits, does follow both longitudinal and latitudinal gradients means that the GWAS for this trait is more affected by the correction for population structure. Applying BLINK without fitting the PC as a covariate, we found the inflation factors for all the populations and traits in this study are within 1.0 ± 0.05, except for the genetic cluster ROM with small population size (*n*=30) and the trait BB for the whole population (IF=1.09). This means that BLINK which uses putative QTNs as covariates in the model already control well for population structure. In the CMLM method, 0–3 PCs were used to correct population structure. Based on the QQ plot and IF (Additional file 2: Figure S6), we find that all models with 0–3 PCs could control population structure for the GWAS, except for the cluster ROM. We also observed that including the genetic clusters as a covariate could improve the conventional genetic model and estimations of variance components (Additional file 1: Table S16).

### Replication is possible in different genetic clusters

Replication will increase the confidence of GWAS association signals and is commonly used in human genetics studies [53]. Replication rate is often low [54], especially for GWAS in tree species based on relatively small sample size. For example, [28] reported that none of the 22 SNPs detected using a large population of ca. 800 were replicated in McKown et al. studies [55, 56]. In this study, based on a significantly larger sample, 21% of the significant SNPs (12/55) with large effect size could be verified or replicated in different genetic groups (Additional file 1: Table S8 and S10). Our most repeatable SNPs are those associated with BB and FD and with a large PVE. This further confirms that QTL detection for small effect of alleles is dependent on specific populations, structure, and the size, but for major alleles with large effect, they are more repeatable. This may explain why we did not find the same SNPs for wood quality traits which were observed in a previous study of smaller population size (517 genotypes) in Norway spruce [37]. However, in contrast to these results for wood quality traits, we successfully detected all independent SNPs (*n*=7) found in a previous study using a subset of data (n=808) [14] and the same method (CMLM) for BB, except one filtered out by variation in quality control in this study, but none for height and DBH. One major difference with growth traits is that BB varies mainly along the longitude and also latitude whereas the growth traits vary along the latitude and are thereby strongly affected by the correction for population structure [14]. It may also be that the genetic architecture of BB differs from that of other traits, although further studies will be needed to unambiguously demonstrate it.

### Only a few genes with large effects are indirectly validated using several datasets

A very important step in GWAS is to validate the candidate genes by the genetic transformation if the studied species can be transformed. Unfortunately, in conifer, a genetic transformation system is so far not developed and transformation in spruce itself has seldom been used (but see Karlgren et al. [57]) and remains a major endeavor. So most studies have simply attempted to replicate the identified QTLs in different datasets and many failed [28, 55, 58]. In this study, in order to ascertain, our results as well as possible, we indirectly validated the identified candidate genes using three independent datasets: (1) a full-sib progeny population located in two northern Swedish field plantations with 1370 individuals, (2) a full-sib clonal population located in three southern Swedish field plantations with 914 genotypes, and (3) RNA-seq data from early and later burst buds. A few candidate genes with large effects were validated in the field plantations. In particular, MAP3K was validated in several field plantations using both exome capture and SNP array data. In addition, differential expression of MAP3K was also shown in early and late buds. While this may not amount to a full validation, we nonetheless feel that it lends significant support to our findings and indicates that transformation of these candidates genes should be a priority.

### MAP3K gene regulates the budburst stage and frost damage and enhances growth

To tolerate cold temperature, plants must be able to perceive a cold signal and transduce it into downstream components that could induce appropriate defence mechanisms [59]. In this study, we found that several candidate genes were associated with FD and also showed a significant difference in gene expression among temperatures. Contig MA_12842 harbors three putative frost tolerance genes MA_12842g0010, MA_12842g0020, and MA_12842g0030 belonging to MAP3K cascades which respond to cold stress [59, 60]. A typical MAP3K cascade contains three protein kinases: MAP kinase kinase kinase (MAP3K), MAP kinase kinase (MAP2K), and MAP kinase (MAPK). In our study, the contig MA_12842 may contain three putative MAP3K according to the current assembly, but it should be verified in the improved Norway spruce reference genome under development. However, all the three associated SNPs (MA_12842_2476, MA_12842_2389, and MA_12842_2490) were located within the same candidate gene MA_12842g0020. Based on strict filtering by 90% of the missing rate per locus in the whole population, we found that all the SNPs in gene MA_12842g00030 were filtered out. Here, to check if the existing of a strong LD between SNPs within two genes MA_12842g00020 and MA_12842g00030, we therefore calculated LD values based on a subset of data (ca.1000 genotypes) with a few missing genotypes for those two genes, and discovered a strong LD between QTL MA_12842_2479 and some of the SNPs in MA_12842g0030 (Additional file 2: Figure S11). Therefore, we putatively considered that the two genes are in strong LD. In *A. thaliana*, MAP3Ks are induced by various abiotic stresses, including cold and drought. In our study, the late budburst and less FD trees all had a high expression level of the MA_12842g0030 in RNA-Seq data. We observed that trees with less expression of MAP3K showed a decrease of more than 6% for height and DBH in the long-term field plantation. Unfortunately, there is no matured conifer transformation system so far to further verify the role of MAP3K in controlling budburst.

Based on predictions from climate modeling, increased growing season of plants in the Northern Hemisphere from global warming may induce earlier budburst with more frequent FD [61, 62]. Considered that with the increased temperature in Sweden, the start of de-hardening and budburst was predicted to be earlier all over the country. Therefore, improving the cold tolerance for Norway spruce and other Northern species is becoming more important. In this study, the detection of SNPs and associated genes underlying the variation of FD and late budburst may greatly improve genomic prediction and genome-wide marker-assisted selection in future Norway spruce breeding.

## Conclusions

In the present study, we used a large data set from 120 progeny tests and newly collected data from clonal archives to carry out the largest association study in Norway spruce. Through factor-analytic and spatial analyses, we made the combination of a diverse and heterogeneous dataset of different ages, sites, and pedigrees with a minimum genetic connection possible to improve the accuracy of phenotypic value for GWAS. While the total number of significant SNPs identified for growth and wood quality traits was limited, we were able to detect more SNPs for phenology traits. It also shows the value of ascertaining the identified SNPs through both replication and verification steps. Indeed, our GWAS study succeeded in identifying, replicating, and verifying through a gene expression study the association between the cold tolerance genes MAP3K and bud burst. Since the SNPs associated with cold tolerance are also associated with tree growth and DBH, by improving cold tolerance, growth, and productivities of forests could be simultaneously increased.

## Methods

### Plant material

We used 483,424 progeny trees and their 5056 unrelated parents in the Swedish Norway spruce breeding program to dissect the genetic basis of phenology, growth, and wood quality traits. These plantations belong to the breeding program managed by the Forestry Research Institute of Sweden (Skogforsk) over the past 50 years which started with elite tree selection and continued with the establishment of progeny tests. The 5056 parents were genotyped using exome capture and either phenotyped directly for some traits or, for other traits, phenotypically characterized through their predicted breeding values obtained from 482,054, predominantly open-pollinated progeny trees, at 120 progeny tests across Sweden (Additional file 2: Figure S12 and Additional file 1: Table S17). Finally, 1370 full-sib progeny trees from 55 parents (51 genotyped and overlapped with the 5056 parents) planted at two different field plantations were genotyped by a standard exome capture and phenotyped to validate the QTLs and 914 full-sib progeny trees from 49 parents (36 genotyped and overlapped with the 5056 parents) were genotyped by a recently developed 50K SNP array [20] and phenotyped to validate the candidate genes associated with BB detected by GWAS, by checking whether any SNPs were detected in the same gene region (Fig. 1). A detailed description of the studied material is given below.

### Elite trees from the Swedish breeding program

The GWAS was based on 5056 unrelated Norway spruce elite trees, which are representative of the whole species geographical range (Fig. 1a). These elite trees were grafted in the 1980s in three clonally replicated common gardens located in southern, central, and northern Sweden. The southernmost common garden is located at Ekebo (55.56 °N, 13.06 °E) with ca. 3386 elite trees, the central common garden in Brunsberg (59. 37 °N, 12.57 °E) and includes ca. 484 elite trees, and the northernmost common garden at Sävar (63.89°N, 20.54°E) comprises ca. 1186 elite trees.

### Progenies used to evaluate the elite trees performance across Sweden

The performance of groups of selected elite trees was evaluated in an extensive network of half-sib, full-sib, and clonal field plantations across the different Swedish breeding zones. Adjacent breeding zones share some materials and can therefore be connected, but more distant ones are genetically disconnected. Here, we selected 483,424 progenies from 122 field plantations with well-documented phenotypes throughout 6–28 years’ field survey (Additional file 1: Table S5). These field plantations were developed ~20–47 years ago and include different number of progenies [49]. The smallest plantation includes only 816 progenies obtained from 25 parental elite trees while the largest has 16,160 trees from 1359 parental elite trees. The selected progenies were sub-divided into three groups: the first group includes 482,054 half/full-sib progenies evaluated at 120 field plantations from which breeding values (EBVs) of parental elite trees were estimated. A second group with 1370 full-sib progenies was planted in two northern Swedish field plantations. This group was sequenced previously [63] and was used here to validate the association signals detected from the first group. The third group with 914 full-sib progenies was planted in three southern Swedish field plantations. This group was used to validate the associated signal for BB detected from the first group.

### Sampling, DNA, and RNA extraction

In total, 6426 (5056+1370) and 914 trees were genotyped by exome capture and a recently developed SNP array, respectively (Table 1). Buds and needles were collected from the selected trees; thereafter, total genomic DNA was extracted using the Qiagen Plant DNA extraction protocol with DNA quantification performed using the Qubit® ds DNA Broad Range Assay Kit (Qiagen, Oregon, USA). To validate the candidate genes for the association signals underlying BB, differential expression analysis was performed using RNA-Seq data from early and late budburst elite trees during five budburst stages in the northern Sävar common garden. In total, we selected 12 genotypes for early and 12 for late budburst and a second tree (ramet) from a single genotype of the early and late budburst categories as a biological replicate (i.e., 13 trees from 12 genotypes for each category). Buds of those genotypes were sampled from the top of trees at 4 days intervals (T1 to T5) from May 4 to May 24, 2018. Sampling time was between 11:00 and 15:00, and ambient temperature was recorded. The buds were kept in liquid nitrogen in the field and stored in −80°C before RNA extraction. Total RNA was extracted using the miRNeasy Micro Kit (Qiagen) according to the manufacturer’s instructions. RNA quantity and purity were assessed by NanoDrop 2000 spectrophotometer (NanoDrop Technologies, Wilmington, DE, USA), and RNA concentration was measured using the Qubit RNA Assay kit Qubit 2.0 Fluorometer (Life Technologies, Carlsbad, CA, USA). RNA integrity was analyzed on an Agilent 2100 Bioanalyzer with Pico chips (Agilent Technologies, Waldbronn, Germany).

### Genotyping by exome sequencing and SNP array

Sequence capture was performed using 40,018 probes previously designed [18], and samples were sequenced to an average depth of 15x on an Illumina HiSeq 2500 platform by RAPiD Genomics (Gainesville, FL, USA). Raw reads were mapped to the *P. abies* reference genome v1.0 using BWA-mem [64, 65]. SAMTools [66] and Picard [67] were used for sorting and removal of PCR duplicates, and the resulting BAM files were subsequently reduced to only include probe bearing scaffolds (24,919) before variant calling. Variant calling was performed using the Genome Analysis Toolkit (GATK) HaplotypeCaller [67] in Genome Variant Call Format (gVCF) output format. Samples were then merged into batches of ~200 before joint variant calling.

The 914 genotypes that were used to evaluate the associated signals with BB were generated using the Norway spruce 50K SNP chip [20].

### Quality control of called SNPs

To improve the quality of called SNPs, several filtering steps were performed: (1) removing indels, (2) keeping only bi-allelic sites, (3) marking sites with a genotype quality (GQ) < 10 as missing, (4) removing sites with call rate (“missingness”) < 70%, (5) removing sites with minor allele frequency (MAF) < 0.005, (6) removing SNPs with an excess of heterozygotes and deviation from Hardy-Weinberg equilibrium test (HWE-test) for the whole elite tree population (*P* value < 1.4e−7), and (7) removing individuals with call rate < 50%. After these filtering steps, a total of ~300K SNPs with MAF > 0.005 were left for population structure analysis. In our previous study [63], with filtering criteria of GQ < 6 and DP < 2 as missing, the averaged discordance estimated from 148 technical replicates was less than 1%. Thus, we considered that such filtering criteria were sufficient for downstream analysis.

Genotype calling of the 50K Axiom array was performed using the Axiom analysis suite (V4.0), following best practice with default parameters (a sample call using a Dish-QC threshold [axiom_dishqc_DQC] ≥ 0.82 and an average SNP call rate cutoff per sample [cr-cutoff] ≥ 0.97). After filtering the SNPs with MAF <0.05, 37819 SNPs were kept for downstream analysis.

### Genotype imputation and estimation of the imputation accuracy for exome sequencing data

We used Beagle v4.1 to impute the missing genotypes [68]. To estimate the imputation accuracy, 216 trees were randomly selected and genotyped with a 450k pilot Axiom SNP array [20]. After removing probes with poor quality, 15,601 SNPs with high confidence calls were retained and further used as a basis to evaluate the imputation quality. Accuracy score is calculated as the ratio of SNPs with the same call derived from array genotypes and genotypes imputed from exome sequencing data. For SNP array, missing loci were imputed by random imputation of the codeGeno function in the synbreed package in R [69].

### Population structure and origin assignation

To infer population structure among the sampled individuals, we used linkage disequilibrium (LD)-trimmed SNPs by removing randomly one SNP from each pair of SNPs when the squared pairwise correlation coefficients (*r*^2^) exceed 0.2 in blocks of 20 SNPs using PLINK 2.0 [70]. This step yielded 200K independent SNPs for downstream analyses of population structure. EIGENSOFT v7.2.1 [71] was used to perform principal component analysis (PCA) on the reduced set of independent SNPs. In this study, the geographic origins of 2277 elite trees were unknown. We therefore used a random forest classification model from missForest function [72] in R package missForest to infer their geographic origins based on genotype similarity on the first five principal components of the PCA. The 2779 elite trees with known geographical origins were used as a training set (Additional file 1: Table S1 and S2). Pairwise *F*_st_ index estimates among subpopulations/genetic clusters defined by PCA and missForest function were estimated using VCFtools [73].

### Phenotyping

In total, we performed GWAS for seven traits, including two growth traits (height and DBH), two phenology traits (BB and FD), and three wood quality traits, wood density (WD), microfibril angle (MFA), and wood stiffness (WS). The three wood quality traits and BB were measured directly on the elite trees in the three common gardens, and GWAS were performed on adjusted phenotypic measurements after removing environmental effects (see below). For the three remaining traits, height, DBH, and FD, estimated breeding values (EBVs) of the elite trees were predicted from 120 progeny tests across Sweden. Importantly, these progeny tests were unevenly distributed, depending on the trait (see below). Furthermore, not all seven traits’ measurements were available for the 5056 elite trees and the validation progeny population of 1370 trees. Table 1 gives a detailed description of the samples used for each trait. In summary, the GWAS included a variable number of samples for each trait ranging from 1428 to 4138 trees and the validation progeny population sample size ranged from 1067 to 1370 trees.

### Phenotyping height, DBH, and FD on 482,054 progenies to estimate breeding values for GWAS on elite trees

For height, 330,966 progeny trees generated from 3838 parental elite trees and grown in 115 progeny plantations were phenotyped at ages varying from 6 to 16 years. DBH was measured on 213,931 progeny trees at age of 10 to 28 years. These progenies descended from 2674 parents and were planted in 75 progeny tests. The 115 plantations for height measurement were distributed across Sweden while 75 plantations (70 overlapped with height measurement) for DBH measurement were distributed in southern and central Sweden (Additional file 1: Table S17). FD was scored as 0/1 in two half-sib progeny tests (F1150 and F1215, Fig. 1a and Additional file 1: Table S17), in which the progenies were from 1428 parental elite trees. Here, 1 represents susceptibility to frost and 0 indicates resistance.

### Phenotyping wood quality traits and BB on the elite trees for GWAS

Three wood quality traits and BB were measured in the three common gardens (Ekebo, Brunsberg, and Sävar, Table 3). WD and MFA traits were measured *directly* on the elite trees using the Pilodyn 6J Forest instrument (PROCEQ, Zurich, Switzerland) in 4127 elite trees with ca. three ramets/clone (clonal copy of the same genotype) each and Hitman ST300 instrument (Fiber-gen, Christchurch, New Zealand) in 4122 elite trees with ca. three ramets each, respectively, in 2016. The WD was measured using depth of needle penetration of the Pilodyn with a 2.0-mm diameter pin, without removing the bark; therefore, WD values represented by penetration depth would be lower when wood density is higher. The MFA was determined by acoustic velocity from the Hitman ST300 measurement. The third trait of WS, also called the modulus of elasticity, was estimated using the equation by combining the Pilodyn penetration and acoustic velocity data [74]. The BB was directly scored following criteria defined by Krutzsch [75] for 4138 elite trees with ca. 3 ramets each in the three common gardens (Tables 1, 3 and Fig. 1a).

**Table 3:** List of the phenotypes, their abbreviations, age of measurement, number of genotypes used in GWAS, and measurement unit

Traits (abbreviation)	Age	No. genotypes	Unit
Budburst stage (BB)	7–25	4138	Category
Tree height (height)	6–16	3838	cm
Diameter at breast height (DBH)	10–28	2674	mm
Frost damage (FD)	6–7	1428	Category
Wood density (WD)	23–31	4127	kg m^-3^
Microfibril angle (MFA)	23–31	4112	(km/S)
Wood stiffness (WS)	23–31	4121	(Gpa)

### Phenotyping height, three wood quality traits, and BB on 1370 progenies to replicate the association signals

To validate the detected association signals from the parental population, we measured five traits including height, three wood quality traits, and BB in 1370 progenies from two northern Swedish full-sib field plantations (Fig. 1a).

### Phenotyping BB on 914 genotypes to replicate the association signals

To validate the detected association signals from the parental population, we measured BB in 914 genotypes, each with ca. three replicates (ramets) at each of three clonal plantations (Table S17).

### Preparing the response variables of GWAS for height, DBH, and FD

To improve the accuracy of response variables used for GWAS, we did three steps of preparation for height, DBH, and FD. The three steps are as follows:

### Adjusting environmental effects for height, DBH, and FD

We used spatial analysis to correct for environmental effects for height, DBH, and FD traits [76]. The details of the spatial models are in Additional file 3: Supplementary methods.

### Estimating breeding values of the adjusted height, DBH, and FD

To predict the estimated breeding values (EBVs) [77] and adjust for plantation and age effects, we employed a reduced parental/animal linear mixed model combined with factor analytic (FA) analysis for multi-environment analysis:

1 $$ y= X\tau +\frac{1}{2}{F}_p{u}_{a_p}+e $$

where *y* is a vector of the spatially adjusted observations, *τ* is a vector of fixed effect, including the grand mean, plantation, and age, ${u}_{a_p}$is a vector of random additive genetic effects for parents, *e* is a vector of random residual terms. *X* and *F*_*p*_ are known incidence matrices for fixed effects and parents, respectively. The random effects in the model are assumed to follow a multivariate normal distribution with mean and variance defined by:

$$ {u}_{a_p}\sim N\Big(0,{\sigma}_a^2{A}_{pp} $$) and $$ e\sim N\left(0,{\sigma}_e^2I\right) $$

where 0 is a null vector; *A*_*pp*_ is the numerator relationship matrix of parents; *I* is the identity matrix, with order equal to the number of trees; and ${\sigma}_a^2 and\ {\sigma}_e^2$ are the additive and residual variances, respectively. To provide a good parsimonious approximation to the unstructured genotype-by-environment covariance matrix, the additive genetic effect *i* at plantation *j* can be expressed as follows:

2 $$ {u}_{a_{p_{ij}}}={\lambda}_{a_{1j}}{f}_{a_{1i}}+{\lambda}_{a_{2j}}{f}_{a_{2i}}+\dots +{\lambda}_{a_{kj}}{f}_{a_{ki}}+{\delta}_{a_{ij}} $$

which includes a sum of *k* multiplicative terms. Each term is the product of an additive genetic effect (${f}_{a_{ri}}\Big)$, which is known as a factor score, and an environment effect (${\lambda}_{a_{rj}}\Big)$, which is known as loading. The *k* of the FA models is the number of factors (multiplicative terms), and we denote an FA model with *k* factors as a FA*k* model. To obtain main additive genetic effects for all parents and also additive genotype by environment effects, an additive effect ${u}_{a_{p_{ij}}}$ could be described as *u*_*a*_ = *m* + *me*, where *m* is the additive main additive genetic effect, and *me* is the additive genetic-by-environment effect. *var*(*u*_*a*_) =  *var* (*m*) + *ɅɅ*^*T*^ + *Ψ*, where Ʌ is a matrix of loadings and *Ψ* is a diagonal matrix with diagonal elements referred to as specific variances. The model is referred to as the FAMK model [78], which is equivalent to a factor analytic model with (*k*+1) factors, where the first set of loadings are constrained to be equal.

To predict EBVs of FD, a logit function was used to transform the binary variable, FD and then a generalized linear mixed model was employed in ASReml V4.1 [79]. The details of the model are shown in supplementary methods.

### De-regressing breeding values of height, DBH, and FD

Using EBVs as phenotypes in genomic prediction may introduce bias and heterogeneity [80]; thus, de-regressed estimated breeding values (dEBVs) for Height, DBH, and FD as pseudo-phenotypes were used to perform GWAS. The dEBVs for individual *i* was obtained as${\hat{u}}_i^{\ast }={\hat{u}}_i/{r}_i^2$, where ${\hat{u}}_i$ is the estimated EBV and ${r}_i^2$ is the reliability of EBVs [80]. The resulting dEBVs were then weighted according to ${w}_i=\left(1-{h}_i^2\right)/\left[c+\left(1-{r}_i^2\right)/{r}_i^2\right)\ {h}_i^2\Big]$, where ${h}_i^2$ is the heritability of the trait estimated as the mean of heritability of field plantations and *c* is the proportion of variance not accounted for by the markers (here assumed to be 50%) [80].

### Preparing the response variables of GWAS for BB and three wood quality traits

Two steps of preparation were used for BB and the three wood quality traits before performing a GWAS. First, we performed a linear mixed model including blocks as fixed effects to adjust for environment effects in each common garden. The model was as follows:

$$ y= X\beta +\frac{1}{2} Zu+e $$

where *y* is the vector of observations, *X* and *Z* are the incidence matrices related to fixed effects (the grand mean and block effects) in vector *β* and random effects in vector *u* (genotype effects) assuming $u\sim N\left(0,{\sigma}_u^2I\right)$, *e* is the vector of residual effects assuming $e\sim N\left(0,{\sigma}_e^2I\right)$. Second, after adjusting for block effects, we used a simple linear model to adjust for common garden effects.

### Estimates of genetic parameters for all seven traits

Genetic parameters were estimated using ASReml V4.1 [79]. Details are given in supplementary methods. The repeatability of the three wood quality traits was estimated in each of the three common gardens. Repeatability of BB was only measured in the Northern Sävar common garden. For growth traits including height and DBH, the pedigree-based narrow-sense heritabilities were estimated [49]. In the present study, we only estimated narrow-sense heritabilities and genetic correlations among growth and wood quality traits in the two full-sib field plantations which were used for SNPs validation and in two half-sib field plantations in which the dEBVs of FD were estimated. Pearson’s correlations and SNP-based genetic correlation among adjusted phenotypic BB, wood quality traits, and dEBVs of height and DBH were estimated by *cor* function in R 3.5.3 [81] and ASReml V4.1, respectively. To compare pedigree and SNP-based narrow-sense heritabilities for FD, height, and DBH, the two plantations with FD data were used to estimate both types of heritability. For BB and wood quality traits, pedigree-based narrow-sense heritability was estimated from the two field plantations used for validation and the SNP-based heritability was estimated from the whole population available using adjusted phenotypic values.

### Screening for SNP association with phenotypic traits

To identify significant SNPs associated with each trait, GWAS was carried out for each of the seven phenotypes using 134,605 SNPs (the MAF > 0.03 to account for the fairly large size of the population) with two methods (BLINK and CMLM) for the whole population and for the large genetic clusters defined by PCA.

One of the main aims of both BLINK and CMLM is to consider cryptic relationships among individuals and control for false-positive detection risk. Thus, here we only needed to consider the effect of population structure for GWAS. We tested both methods without additional corrections than the ones inherently included in the method, or by including 1 to 3 PCs to control for population structure confounding effect. To assess the efficiency of the methods used here in controlling for population structure, we used the genomic inflation factor (IF). The latter expresses the deviation of the observed *p* values compared with the distribution of the expected *p* values following a uniform distribution. We found that BLINK model with fitting one PC as a covariate or even without fitting PCs is effective in accounting population structure (IF, 0.95< lambda <1.05) [82] and CMLM without correction or 1 to 3 PCs also could control population structure based on the previous criteria. The QQ plots and lambda for all traits including 0 and 3 PCs for CMLM and 0 and 1 PC for BLINK are shown in Additional file 2: Figure S2. Manhattan plots were made using the QQMAN R package [83]. The *LDheatmap* function implemented in the R package LDheatmap [84] was used to illustrate the pairwise LD between all genotyped SNPs, using allelic squared correlation coefficient (*r*^2^). Independent SNPs were defined by pairwise SNPs in different contigs or with LD *r*^2^ < 0.2 in the same contig. Proportion of variance explained by a given SNP (PVE) and cumulative phenotypic variance (*R*^2^) explained by all associated SNPs was calculated according to the method described in [85] using a linear mixed model procedure with genomic relationship matrix to control the cryptic relationship among individuals within sub-populations.

### Replication of the GWAS signals using five progeny field plantations

The 1370 full-sib progenies from 55 parents/elite trees and established in two field plantations (Vindeln and Hädanberg) in northern Sweden that were genotyped in a previous study [63] were used for validation. Here, 51 parents (95%) belonged to the 1041 northern Swedish population (NFE) (Table 1). Genomic-based relationships between 51 parents and the remaining of NSE were shown in Additional file 2: Figure S13. Approximate zero relationships were found between validation parents and other parents in NSE, except for 16 parents. We employed a MLM method [85] with a relationship matrix to test whether SNP associated with BB, height, and the three wood quality traits were also detectable in these two field plantations.

The 914 full-sib progenies from 32 control-pollinated families with 49 parents/elite trees and established in three field plantations in southern Sweden that were genotyped by the recently developed SNP array [20] were used for validation and check whether the identified gene model using exome can be verified and how much percentage of associated signals were located outside of gene model. Thus, we employed the BLINK method to test if SNPs associated with BB. After spatial analysis, we obtained the adjusted phenotypic values for each tree. The mean values of 914 genotypes were used as phenotypic values in BLINK.

### Annotation for significantly associated effects

Significantly associated SNPs at 5% false discovery rate (FDR) were annotated using SnpEff 4.3 with default parameters [86]. The general transfer format (GTF) from Ensembl was utilized to build the *P. abies* SnpEff database. To assess the variant effect of the associated SNPs, annotation of the putative genes and their associated orthologs was performed using the *P. abies* v1.0 genome on ConGenIE database (http://congenie.org/). Candidate gene with an associated SNP inside was extracted from ConGenIE. Due to the fast LD decay with averaged *r*^2^ values dropping to 0.2 at 37 base pairs (bps) within all contigs and 67 bps within all contigs harboring associated SNPs (Additional file 2: Figure S3), and a possible exception up to ca. 1.5 kb for some contigs (Baison et al. 2019), candidate genes were selected from annotated genes that were associated with SNPs inside the gene body only. Besides, the candidate genes located on the same contig harboring a SNP associated with BB and FD, which showed an expression difference in the RNA-Seq experiment described later or had a similar functional annotation were also extracted. For *P. abies* contigs that harbored significant SNPs without any annotated genes, nucleotide BLAST (BLASTN) searches, using the option for only highly similar sequences (MEGABLAST) in the National Center for Biotechnology Information (NCBI) nucleotide collection database (https://blast.ncbi.nlm.nih.gov/Blast.cgi), were performed to manually evaluate possible candidate genes.

### Identifying candidate genes using differential expression analysis on RNA-seq data

The pooled libraries were sequenced on the Illumina NovaSeq 6000 System (2*150 bp) using the paired-end module. The raw reads were processed using fastp [87]. The average insert size for the paired-end libraries was 200–300 bps. The transcript abundances were estimated using kallisto software [88]. Read counts using TPM (transcripts per kilobase million) were normalized and variance stabilized transformed (VST) to obtain gene expression values using the R package “sleuth” [89].

## Supplementary Information

**Additional file 1: Supplementary Table S1-S17.**

**Additional file 2: Supplementary Figure S1-S13.**

**Additional file 3: Supplementary Methods.**

**Additional file 4.** Review history.

## Data Availability

The exome capture raw reads and the RNA-seq data have been deposited in NCBI’s sequence read archive (SRA) under accession number (PRJNA731384) [90]. Background information, adjusted phenotypic data for all elite trees used in GWAS, and phenotypic budburst data and SNP array data for SNP validation are available from the Zendo [91]. Validation phenotypic data for norther two plantation. All scripts used for the analysis are available on GitHub under an MIT licence [92]. Raw phenotypic data are available from the data owner (Forestry Research Institute of Sweden) upon reasonable request.
