# Publisher Correction: Leveraging breeding programs and genomic data in Norway spruce (*Picea abies* L. Karst) for GWAS analysis

**DOI:** 10.1186/s13059-021-02421-z

**Published:** 2021-07-15

**Authors:** Zhi-Qiang Chen, Yanjun Zan, Pascal Milesi, Linghua Zhou, Jun Chen, Lili Li, BinBin Cui, Shihui Niu, Johan Westin, Bo Karlsson, Maria Rosario García-Gil, Martin Lascoux, Harry X. Wu

**Affiliations:** 1 Umeå Plant Science Centre, Department Forest Genetics and Plant Physiology, Swedish University of Agricultural Sciences, SE-90183 Umeå, Sweden; 2 Program in Plant Ecology and Evolution, Department of Ecology and Genetics, Evolutionary Biology Centre and SciLifeLab, Uppsala University, Uppsala, Sweden; 3 College of Life Sciences, Zhejiang University, Hangzhou, 310058 Zhejiang China; 4 College of Biochemistry and Environmental Engineering, Baoding University, Baoding, 071000 Hebei China; 5 Beijing Advanced Innovation Centre for Tree Breeding by Molecular Design, Beijing Forestry University, Beijing, China; 6 Skogforsk, Box 3, SE-91821 Sävar, Sweden; 7 Unit for Field-Based Forest Research, Swedish University of Agricultural Sciences, SE-90183 Umeå, Sweden; 8 Skogforsk, Ekebo 2250, SE-26890 Svalöv, Sweden; 9 CSIRO National Collection Research Australia, Black Mountain Laboratory, Canberra, ACT 2601 Australia

**Publisher Correction to: Genome Biol 22, 179 (2021)**

**https://doi.org/10.1186/s13059-021-02392-1**

Following publication of the original paper [1], it was reported that Tables 1–3 were published in the incorrect sequence. Tables 1, 2 and 3 were published as Tables 3, 2 and 1 respectively.

Furthermore, there was an error in Fig. 2 and the correct Fig. 2 is supplied below.

**Fig. 2:**
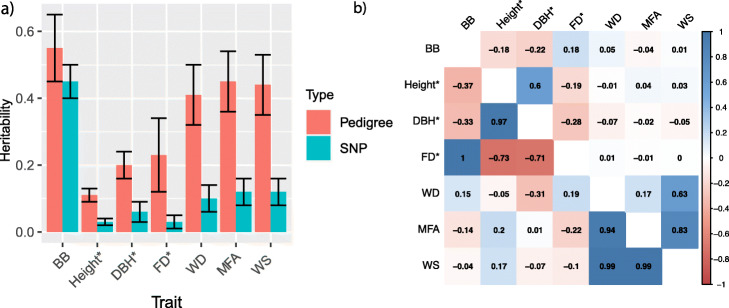
Heritabilities and genetic correlations among traits. **a** Pedigree-based and SNP-based narrow-sense heritability of the seven traits, with black line representing the ± standard errors. **b** Pair-wise Pearson product-moment correlations (upper diagonal) and genetic correlations (lower diagonal) among deregressed estimated breeding values (dEBVs) of tree height (height), diameter at breast height (DBH), and frost damage (FD), adjusted phenotypic values of budburst stage (BB), wood density (WD), microfibril angle (MFA, represented here by acoustic velocity), and wood stiffness (WS). The color spectrum, bright blue to bright red presents highly positive to highly negative correlations, and the number represents the correlation values. * The SNP heritability only explained the proportion of dEBVs variance explained by SNP-based genomic relationships, and dEBVs of the three traits were used to estimate the phenotypic and genetic correlations in b

The original article [1] has been corrected. The publishers apologize for the error.
