# Supplementary material for: Leveraging breeding programs and genomic data in Norway spruce (Picea abies L. Karst) for GWAS analysis
Source: Genome Biol. 2021 Jun 13;22:179. doi: 10.1186/s13059-021-02392-1 (PMC8201819; doi:10.1186/s13059-021-02392-1)
Supplement: Supplementary file 2 — Additional file 2: Supplementary Figure S1-S13. [file 13059_2021_2392_MOESM2_ESM.pdf]

Supplementary Figures

Figure S1 Histogram of agreements between genotypes estimated by SNP array genotyping and the imputed genotypes obtained from exome sequencing across **a**) all individuals by the marker and **b**) all markers by the individual.

Figure S2 Percentage of the variance of the first 10 principal components explained for marker matrix.

Figure S3 Distribution of seven traits. **a**) De-regressed breeding values (dEBVs) of tree height (Height) and **b**) diameter at breast height (DBH) and **d**) frost damage (FD) were predicted from the all progeny field plantations in Sweden, **c**) budburst stage (BB), **e**) wood density (WD), **f)** microfibril angle (MFA), and **g**) wood stiffness (WS) were adjusted phenotypic value from the three common gardens.

Figure S4 Influence of trees origin on de-regressed breeding values (dEBVs) or adjusted phenotypic values of seven traits. **a**) Tree height (Height), **b**) diameter at breast height (DBH), **c**) budburst stage (BB), **d**) frost damage (FD), **e**) wood density (WD), **f**) microfibril angle (MFA), and **g**) wood stiffness (WS) are represented for the different genetic clusters (Carpathian, ROM; Alpine, ALP; Central Europe, CEU; Northern Poland, NPL; Russia-Baltic, Rus-Bal; central and southern Sweden, CSE; Fennoscandian, NFE). The genetic clusters are ordered regarding latitude for Height, DBH, FD, WD, MFA, WS, and given longitude for BB. Letters represent the levels of significance.

Figure S5 The relationship between seven phenotypic traits and latitude or longitudinal variables for subset population with geographical information recorded. They are **a**) tree height (Height), **b**) diameter at breast height (DBH), **c**) frost damage, **d**) budburst stage, **e**) wood density, **f**) microfibril angle, and **g**) wood stiffness. A generalized linear model is fitted between the phenotypic trait and latitudinal or longitudinal variables. r^2 is the coefficient of determination of the model fitting. *p*-value represents the significance of the Pearson correlation coefficient.

Figure S6 Quantile-Quantile (QQ) plots and genomic inflation factors (IF) for all genome-wide associations (GWAs). The red line means inflation value. A) QQ plots using BLINK with one principal component as a covariate for all seven traits including budburst stage (BB) for **a**) the whole population (BB_Whole), **b**) central and southern Sweden (BB_CSE), **c**) Alpine (BB_ALP), **d**) Fennoscandian (BB_NFE), **e**) Central Europe (BB_CEU), **f**) Northern Poland

(BB_NPL), and **g**) Russia-Baltic (BB_Rus-Bal), **h–m**) diameter at breast height (DBH), **n**) frost damage (FD), **o–u**) tree height (Height), **v–ab**) wood stiffness (WS), **ac**–**ai**) wood density (WD), and **aj-ap**) microfibril angle (MFA) for different genetic clusters as for BB; B) QQ plots using BLINK with no principal component as a covariate for all the traits and genetics clusters as in A); C) QQ plots using CLMM method without two or three principal components as covariates for all the traits and genetics clusters as in A); D) QQ plots using CLMM method with no principal component as a covariate for all the traits and genetic clusters as in A).

Figure S7 Decay of linkage disequilibrium (LD) in exome capture genome: **a**) Decay of LD across all SNPs within all contigs. **b**) Decay of LD across SNPs within all associated contigs. The x-axis is the distance in nucleotides; y-axis the correlations coefficients (*r*2) between nucleotides sites.

Figure S8. The relationship between effect size and minor allele frequency for all SNPs associated with budburst stage (BB).

Figure S9 The expression difference in TPM (Transcripts Per Kilobase Million) of 43 genes related to budburst stage and frost damage. **a**) Expression difference of gene MA_10100176g0010, in buds with early budburst and late budburst in five sampling times (T1–T5) and also the temperatures from 5.5 to 25 ℃. **b–aq**) the expression difference of the rest of 42 genes in buds with early budburst and late budburst in five sampling times (T1–T5) and also the temperatures from 5.5 to 25 ℃.

Figure S10 Relationship between the underlying number of QTLs and both required tree number and threshold of the percentage of variance explained (PVE) detectable for individual QTLs under 80% power and a significance level of $1×10^{-7}$.

Figure S11 Linkage disequilibrium (LDs) between SNPs in the gene model MA_12842g100030 and the significant SNP MA_12842_2490 in contig MA_12842.

Figure S12 Left side of Figure: three sets of Norway spruce plant materials used in our GWAS: 1) 5506 elite trees drafted in three common gardens (dark red triangle), 2) 1370 full-sib progenies planted in two full-sib field plantations (green star), and 3) 120 progeny plantations (orange dots). Middle of the figure, 1) we showed the traits phenotyped in how many numbers of field plantations and common gardens (n). De-regressed breeding values (EBVs) estimated from 120 progeny plantations for Height, DBH, and frost damage (FD) were used as the adjusted phenotypic values

for GWA. The adjusted direct measurement of budburst stage (BB), wood density (WD), microfibril angle (MFA), and wood stiffness (WS) were used as adjusted phenotypic values for GWA. The right side of Figure: the traits were measured and adjusted phenotypic values for wood quality traits, and de-regressed EBVs of Height, were used for causal variants validation in two full-sib plantations.

Figure S13 Relationship values between parents and other elite trees in the Fennoscandia group (NFE) population.

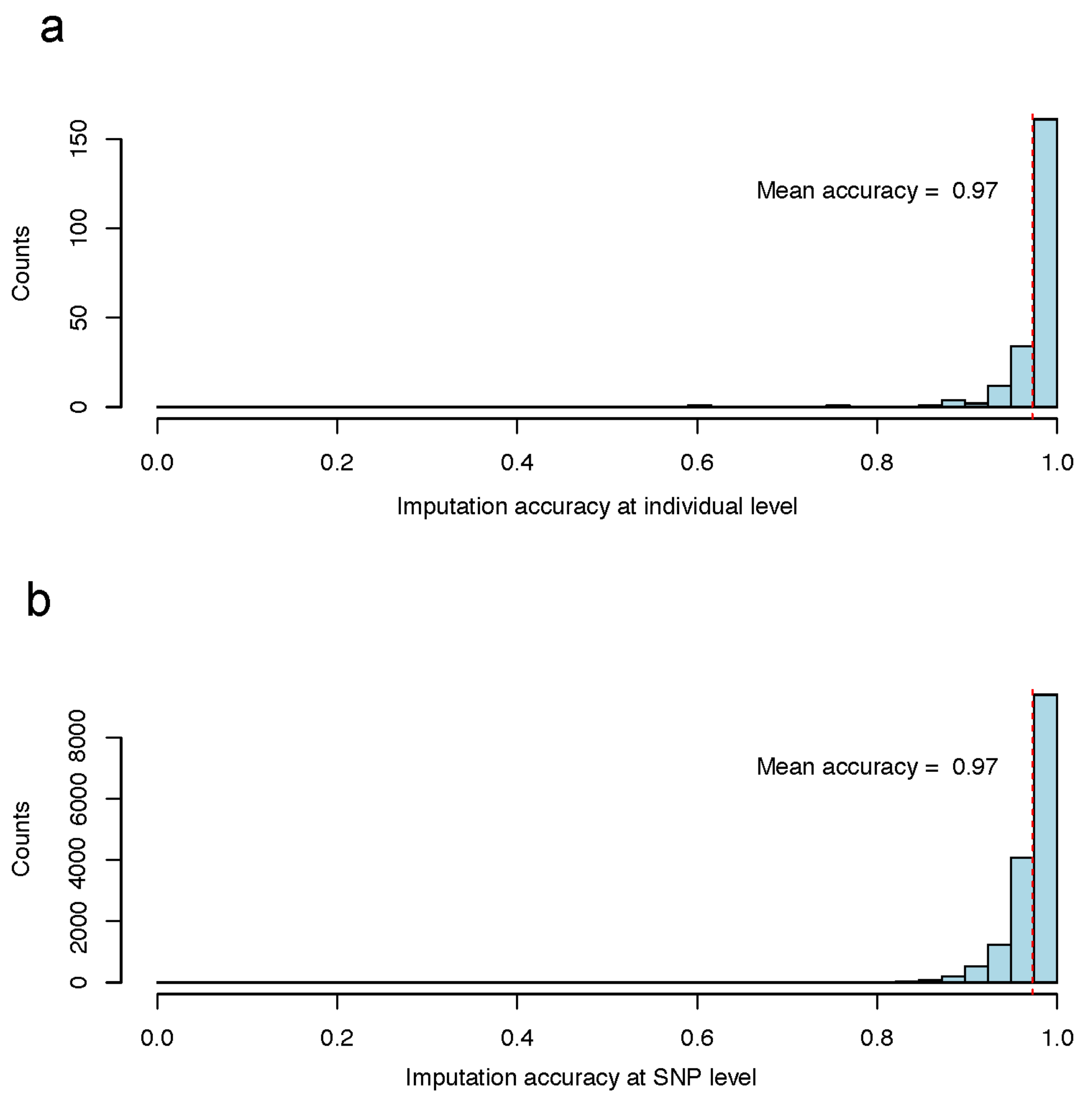

Figure S1 Histogram of agreements between genotypes estimated by SNP array genotyping and the imputed genotypes obtained from exome sequencing across a) all individuals by the marker and b) all markers by the individual.

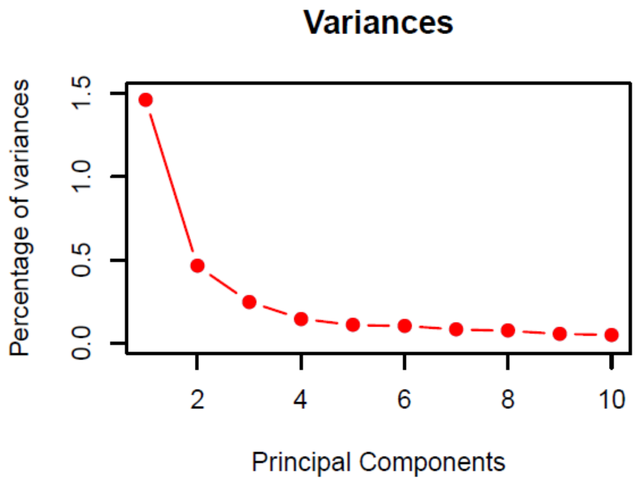

Figure S2 Percentage of the variance of the first 10 principal components explained for marker matrix.

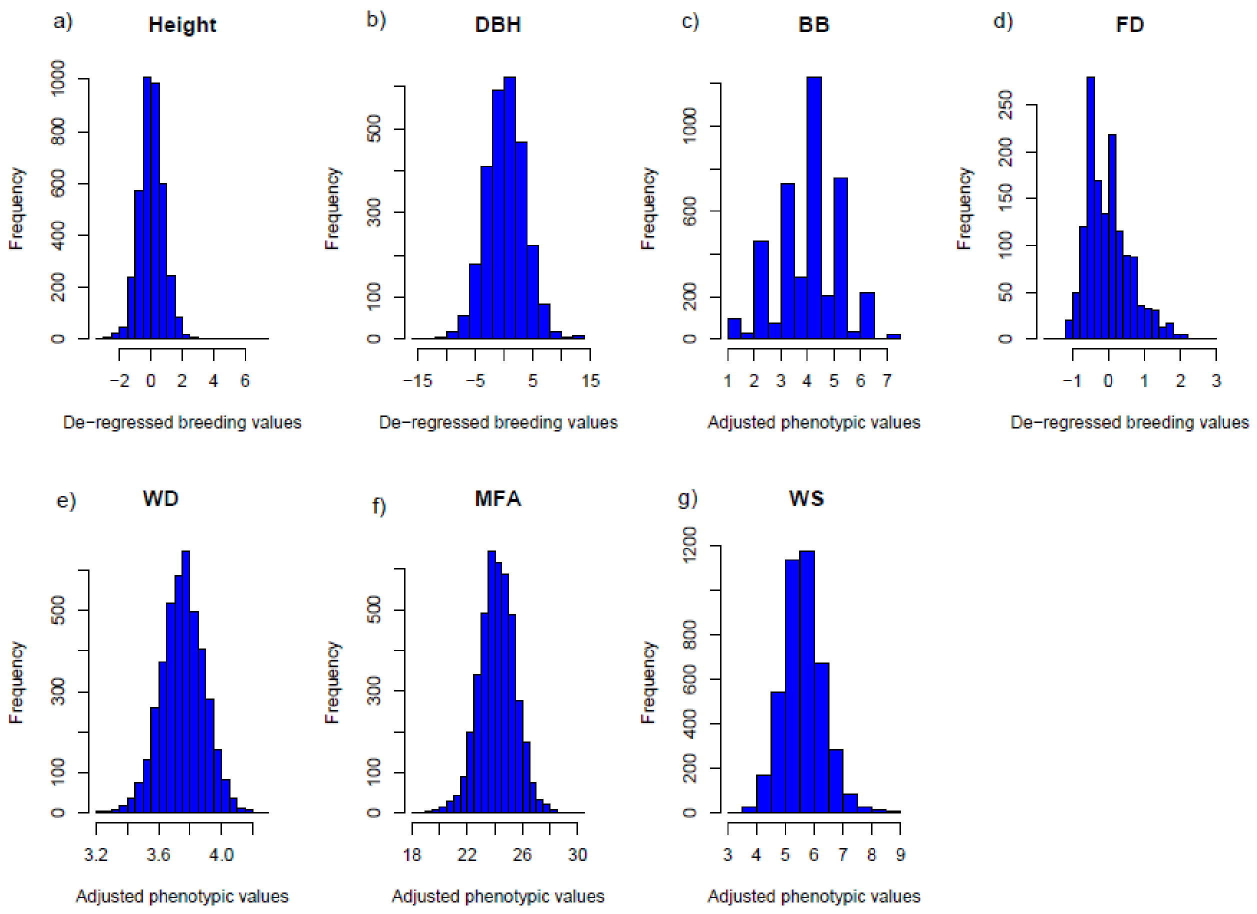

Figure S3 Distribution of seven traits. **a**) De-regressed breeding values (dEBVs) of tree height (Height) and **b**) diameter at breast height (DBH) and **d**) frost damage (FD) were predicted from the all progeny field plantations in Sweden, **c**) budburst stage (BB), **e**) wood density (WD), **f)** microfibril angle (MFA), and **g**) wood stiffness (WS) were adjusted phenotypic value from the three common gardens.

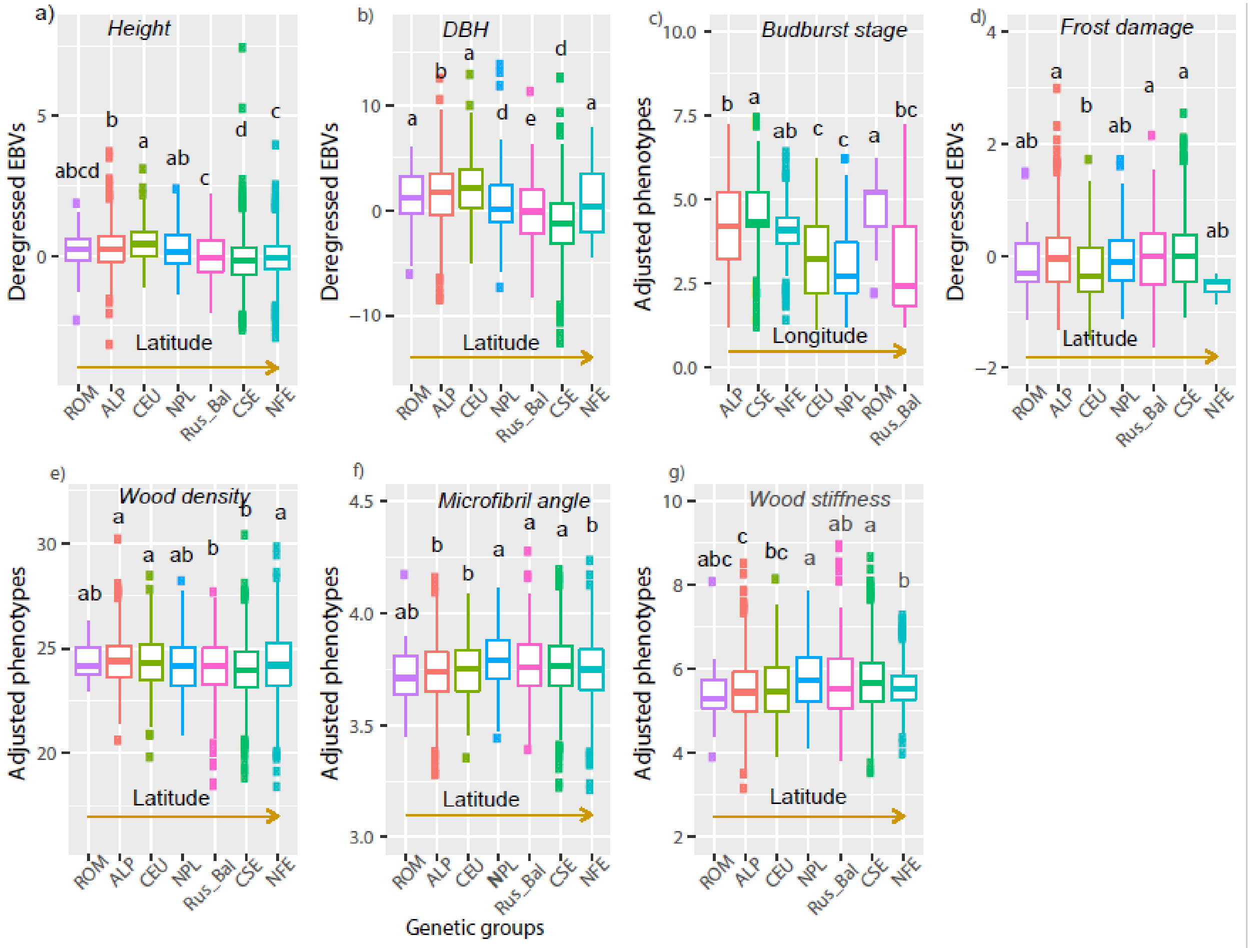

Figure S4 Influence of trees origin on de-regressed breeding values (dEBVs) or adjusted phenotypic values of seven traits. a) Tree height (Height), b) diameter at breast height (DBH), c) budburst stage (BB), d) frost damage (FD), e) wood density (WD), f) microfibril angle (MFA), and g) wood stiffness (WS) are represented for the different genetic clusters (Carpathian, ROM; Alpine, ALP; Central Europe, CEU; Northern Poland, NPL; Russia-Baltic, Rus-Bal; central and southern Sweden, CSE; Fennoscandian, NFE). The genetic clusters are ordered regarding latitude for Height, DBH, wood density (WD), microfibril angle (MFA), wood stiffness (WS), and frost damage (FD), and given longitude for budburst stage (BB). Letters represent the levels of significance.

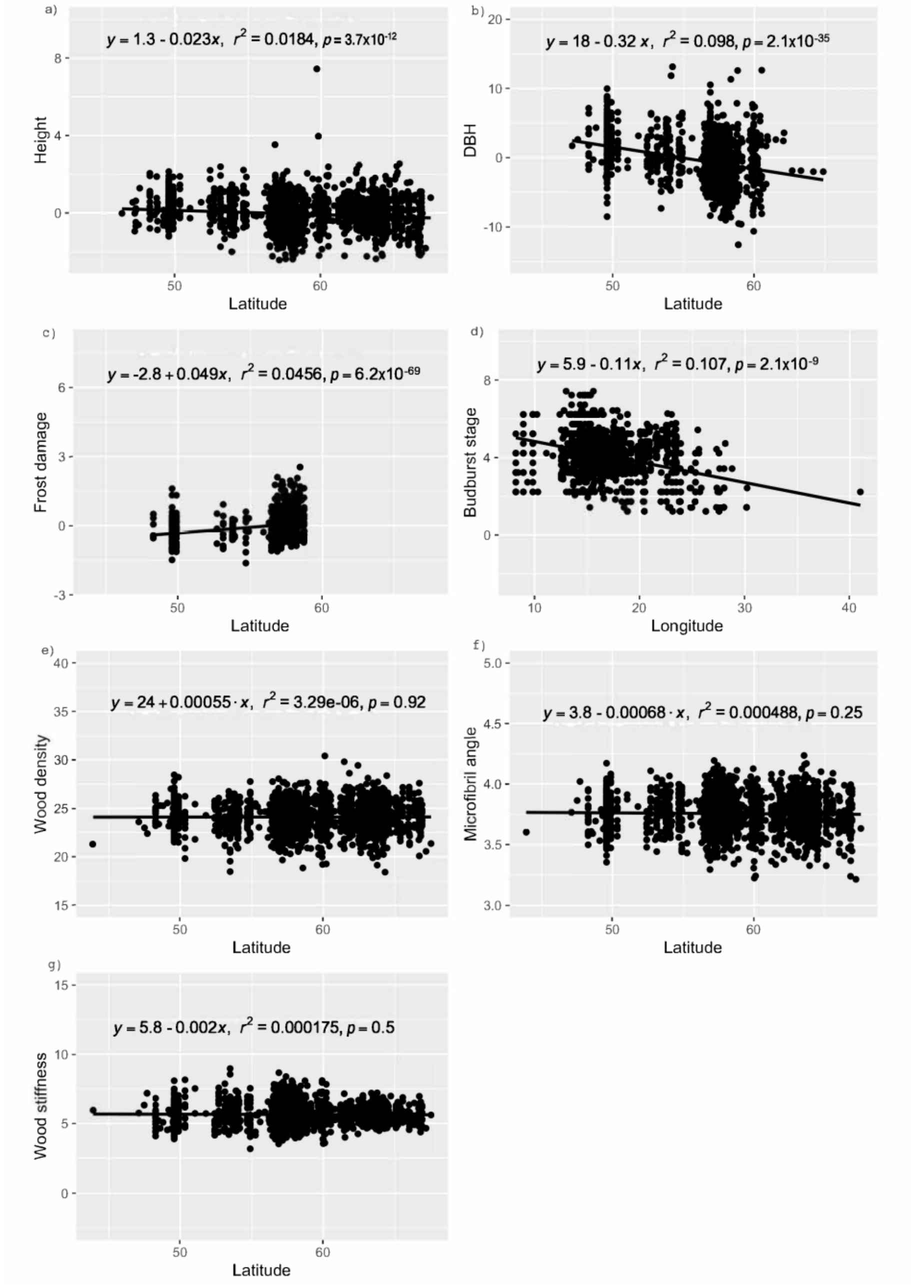

Figure S5 The relationship between seven phenotypic traits and latitude or longitudinal variables for subset population with geographical information recorded. They are **a**) tree height (Height), **b**) diameter at breast height (DBH), **c**) frost damage, **d**) budburst stage, **e**) wood density, **f**) microfibril angle, and **g**) wood stiffness. A generalized linear model is fitted between the phenotypic trait and latitudinal or longitudinal variables. r^2 is the coefficient of determination of the model fitting. *p*-value represents the significance of the Pearson correlation coefficient.

A) BLINK with one principal component as a covariate

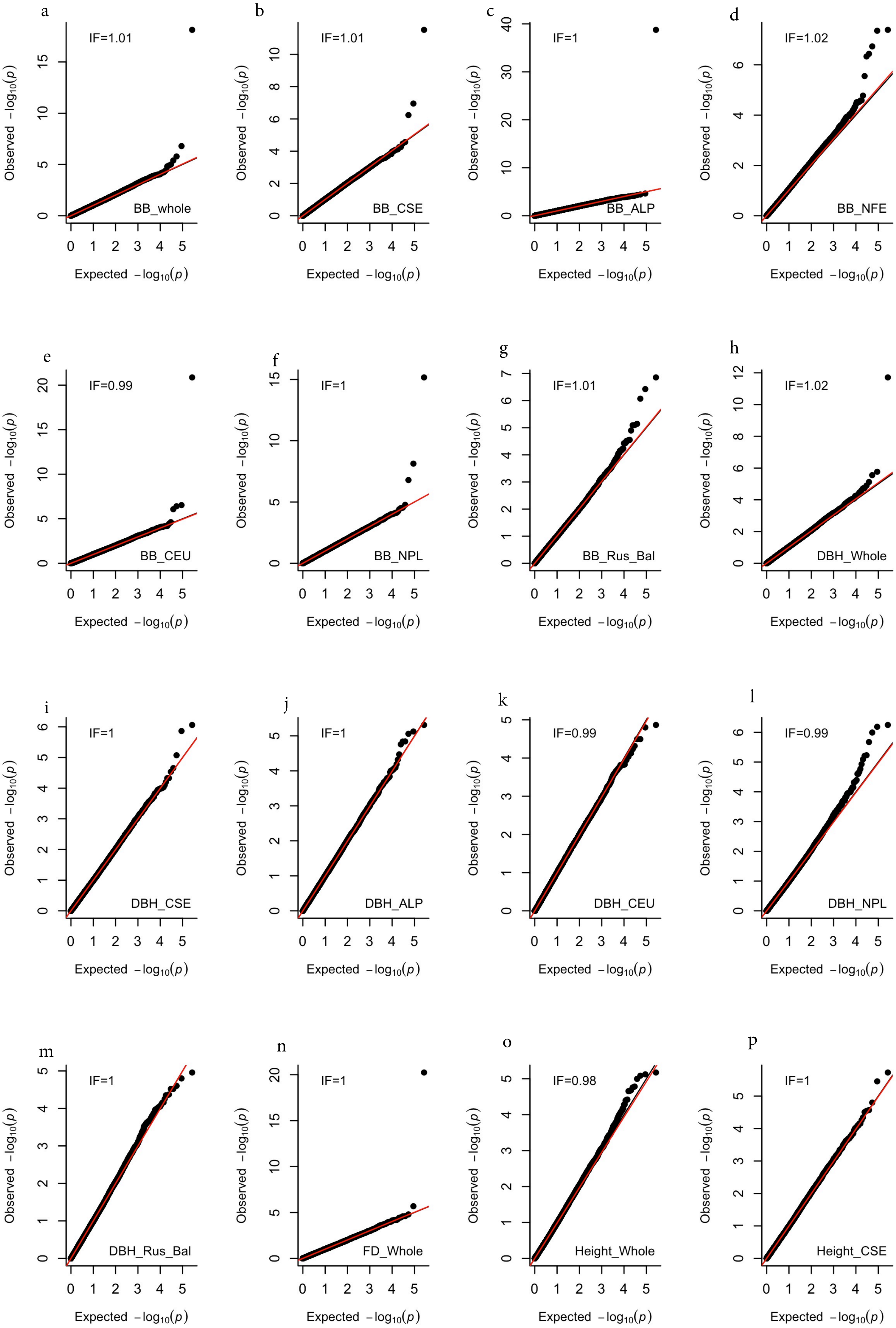

A) BLINK with one principal component as a covariate

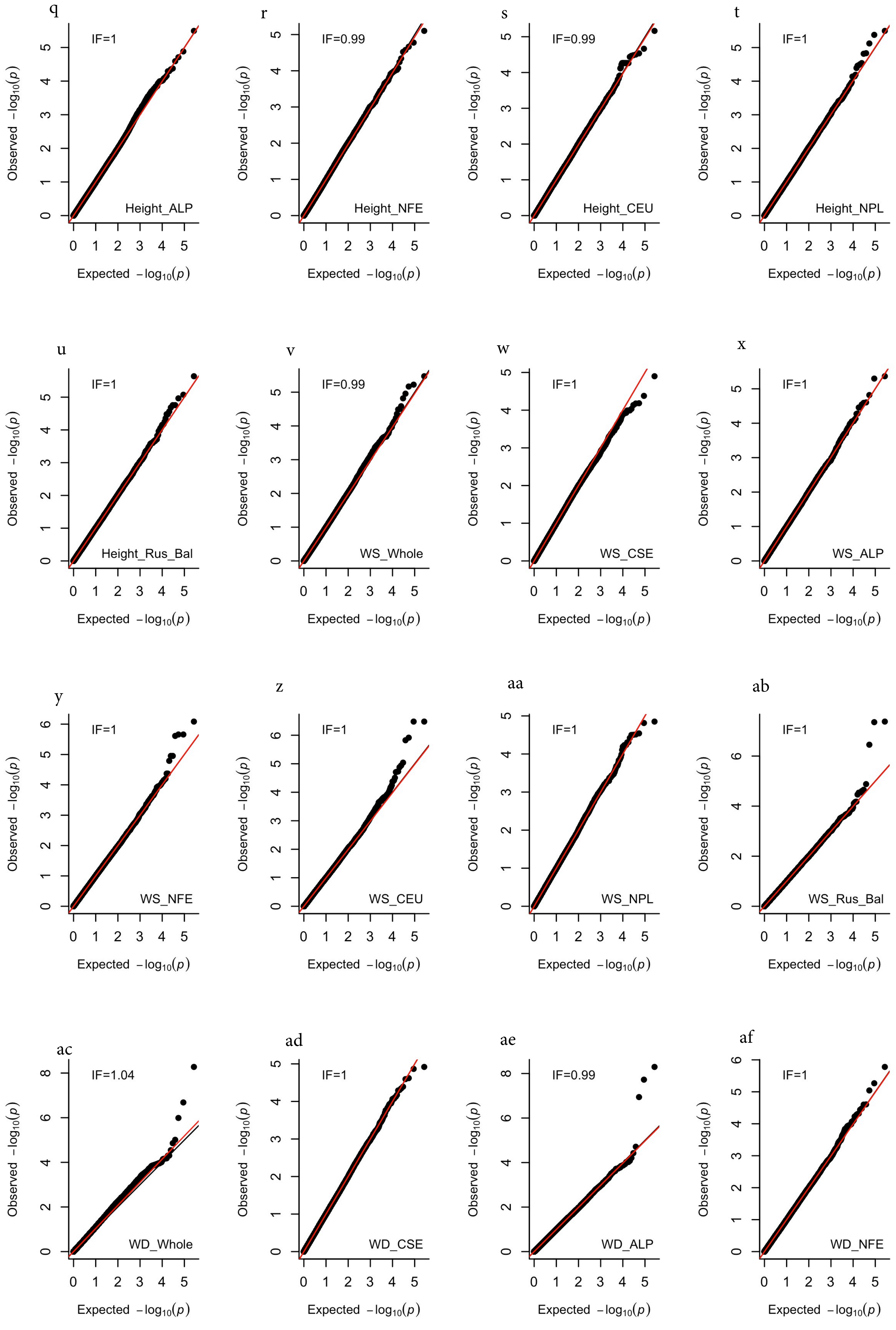

A) BLINK with one principal component as a covariate

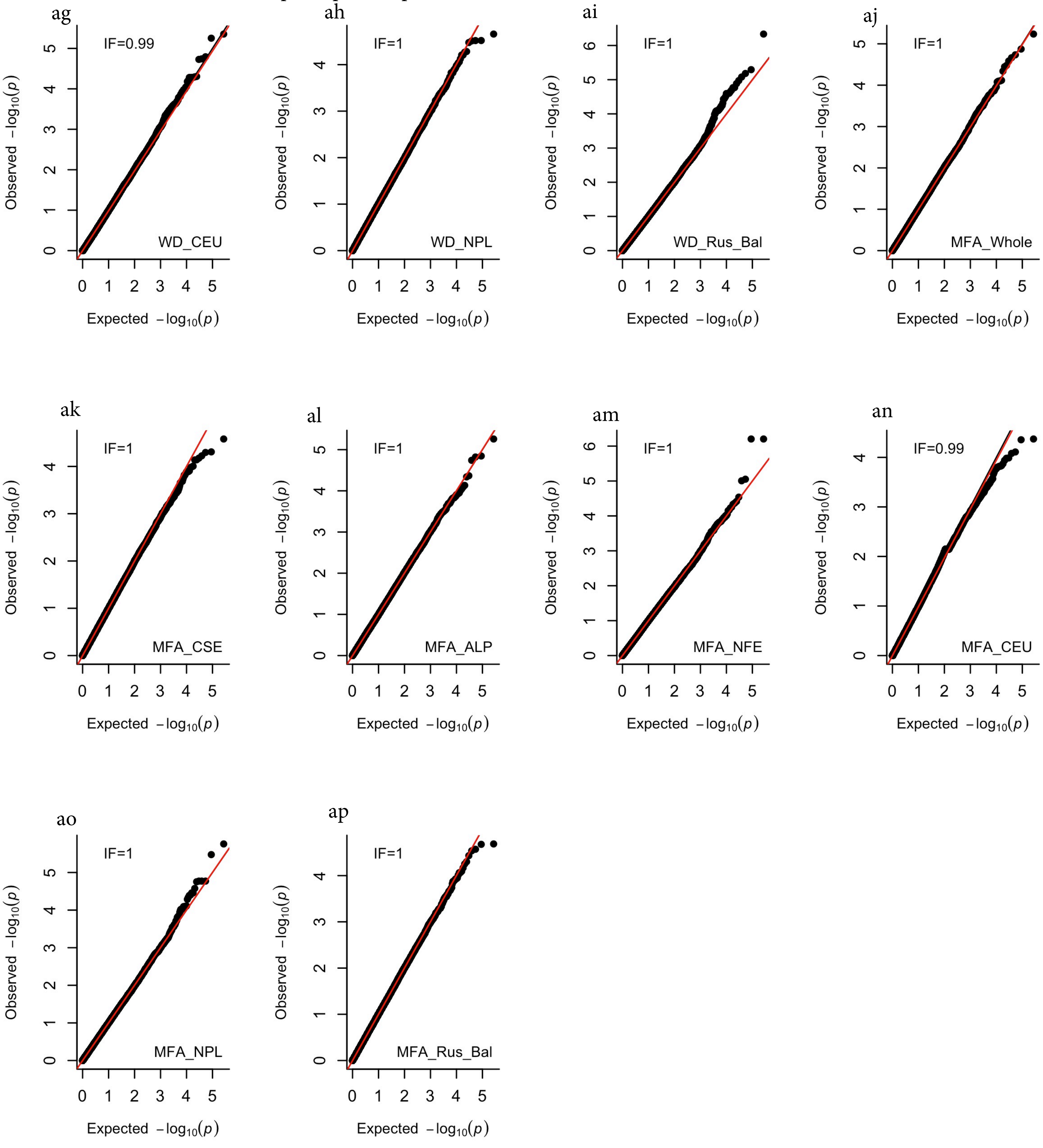

B) BLINK with no principal component as a covariate

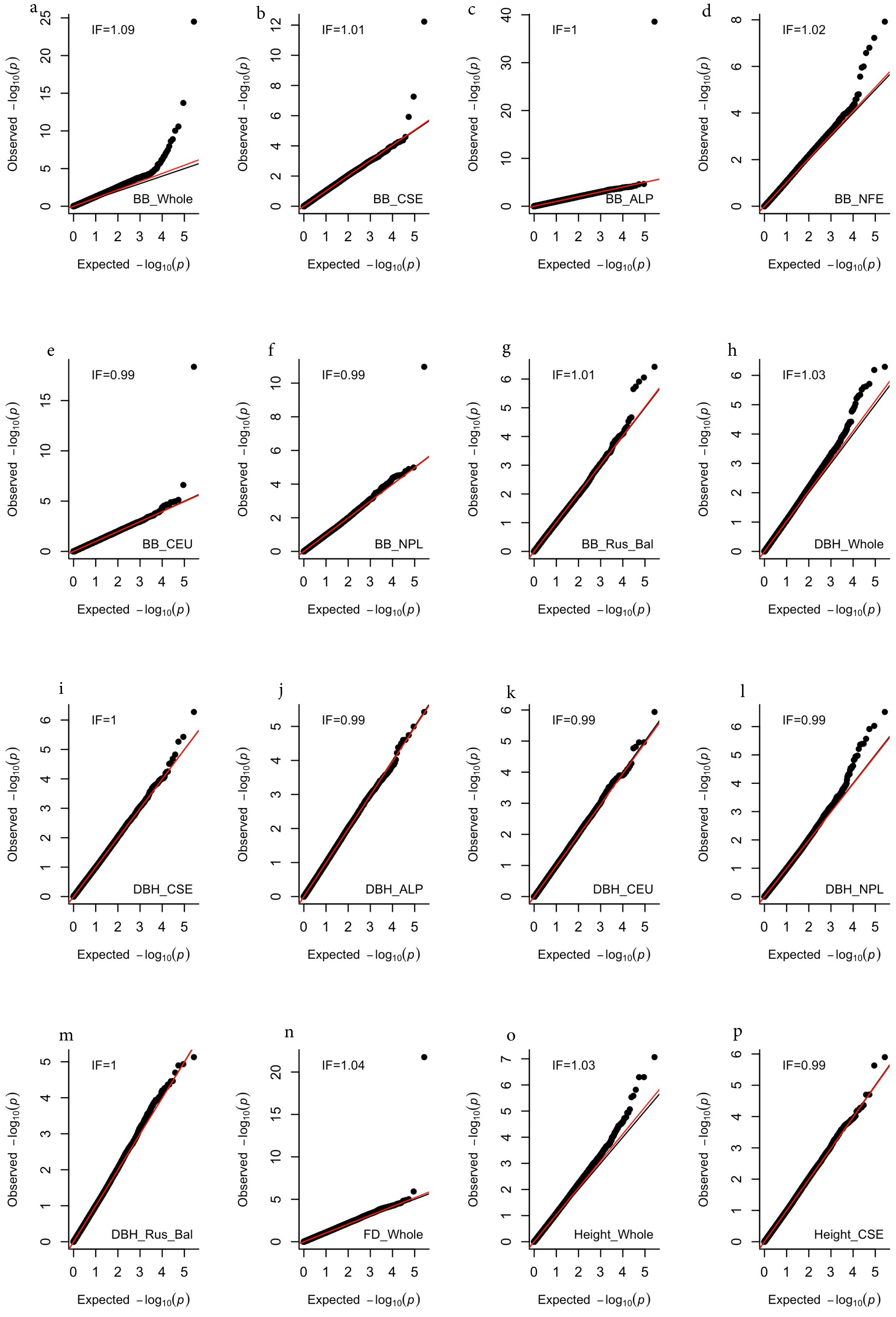

B) BLINK with no principal component as a covariate

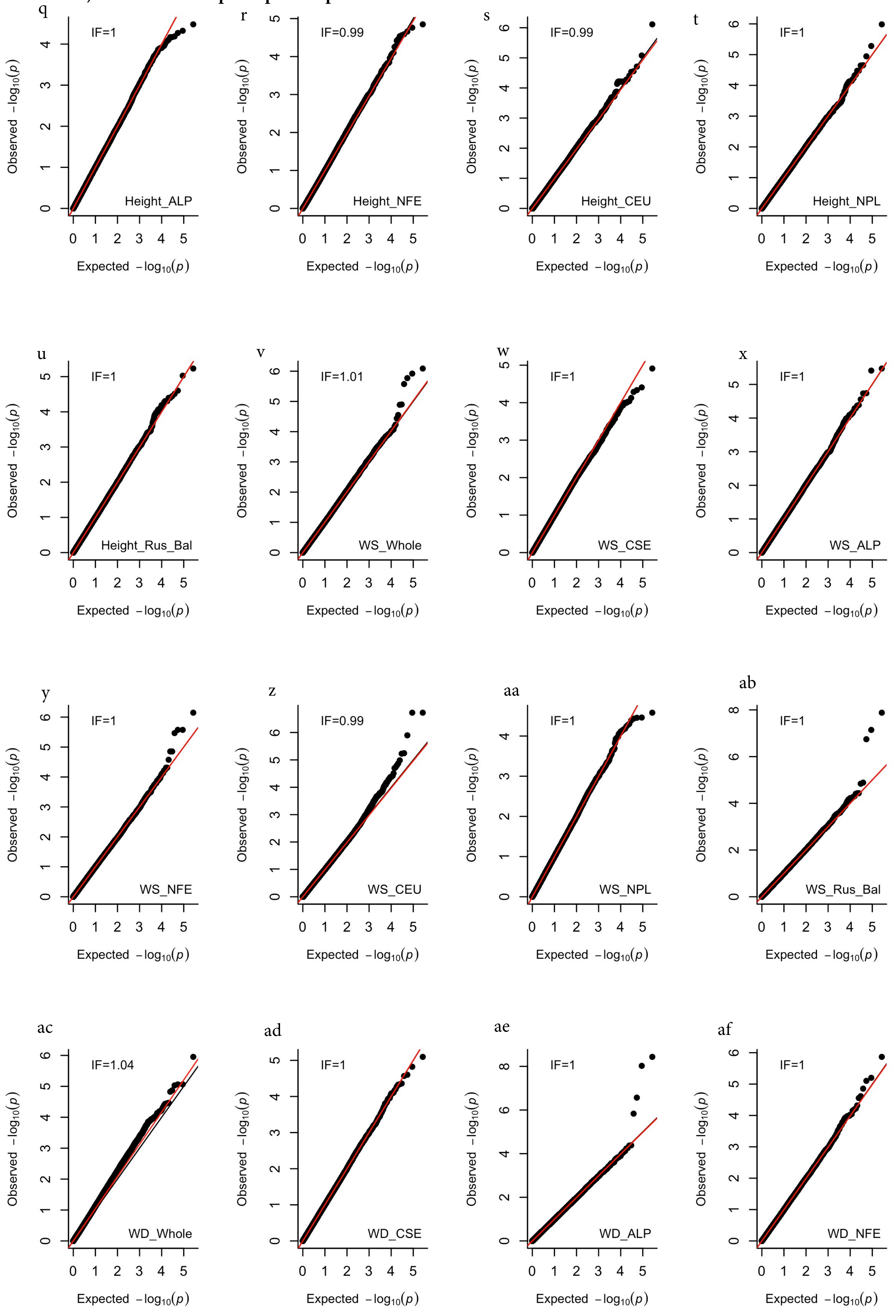

B) BLINK with no principal component as a covariate

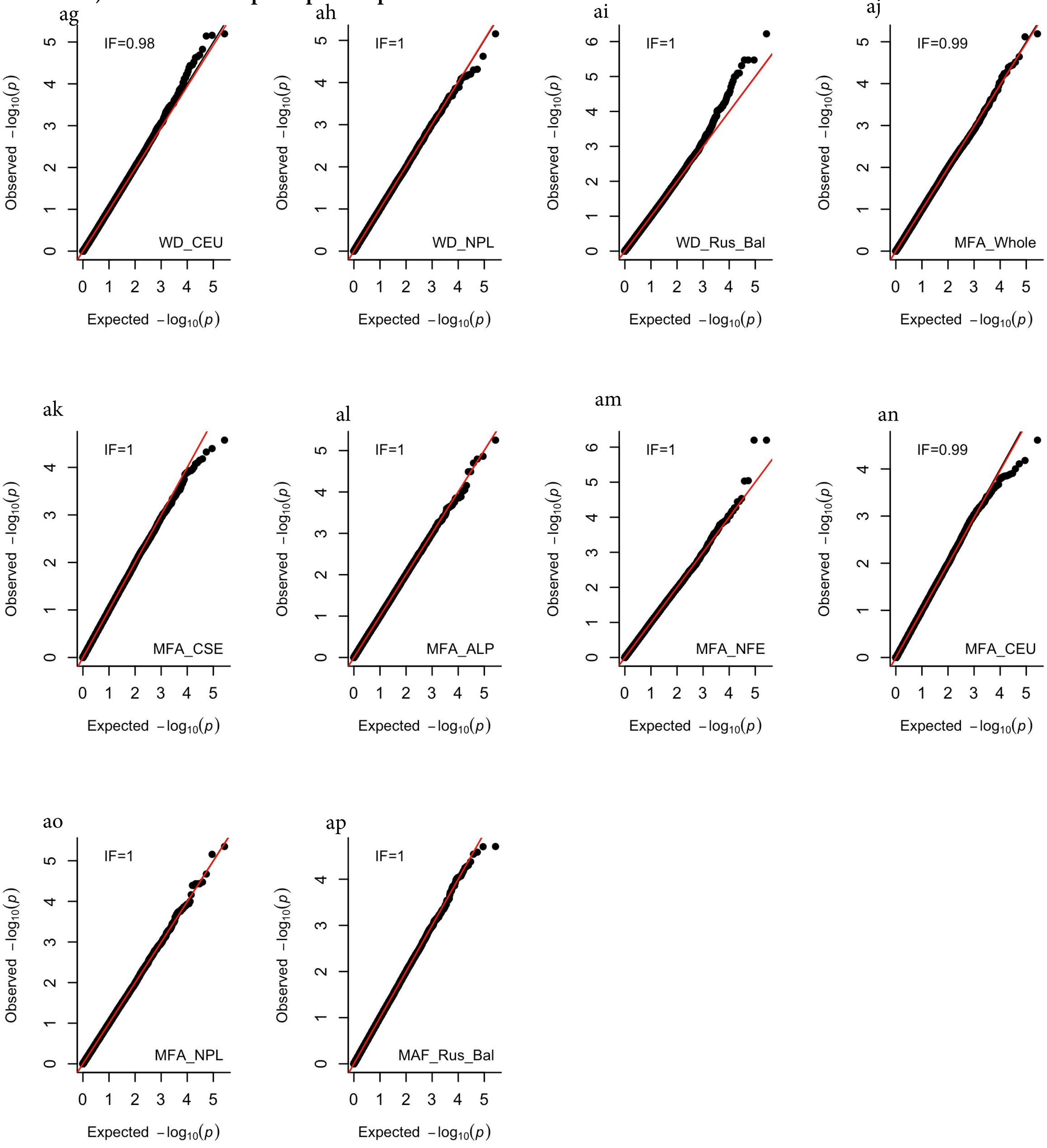

C) CMLM with two or three principal components as covariates

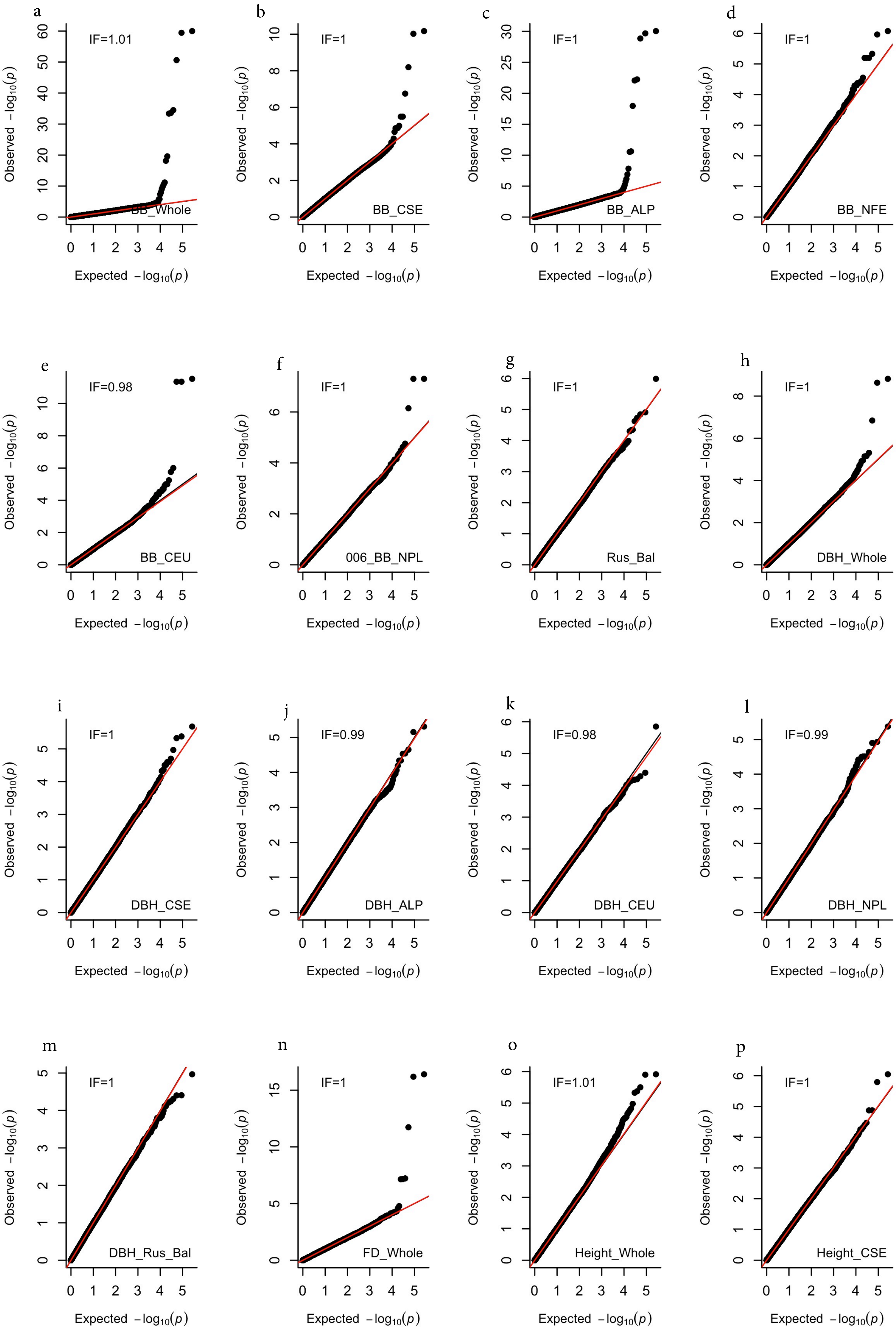

C) CMLM with two or three principal components as covariates

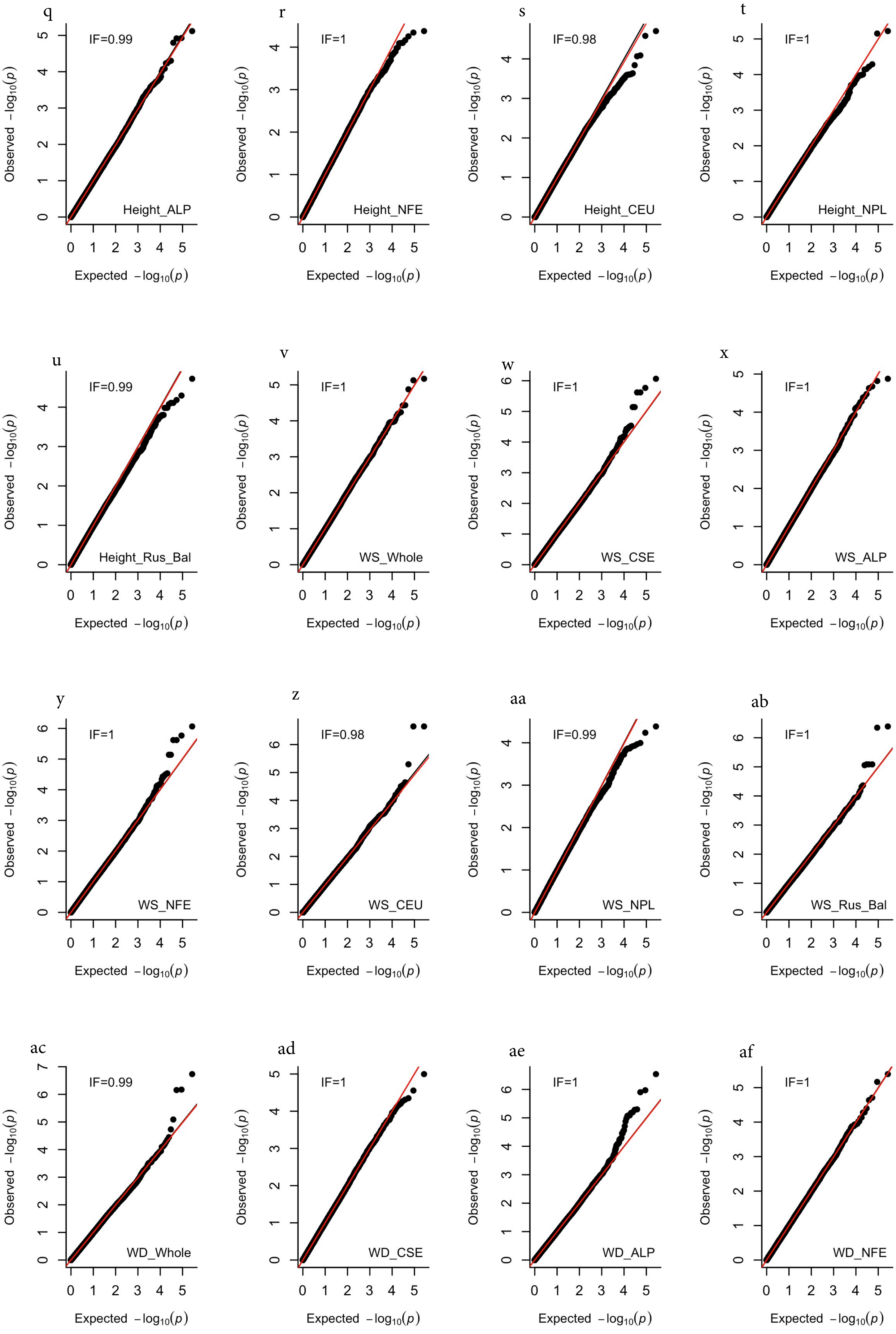

C) CMLM with two or three principal components as covariates

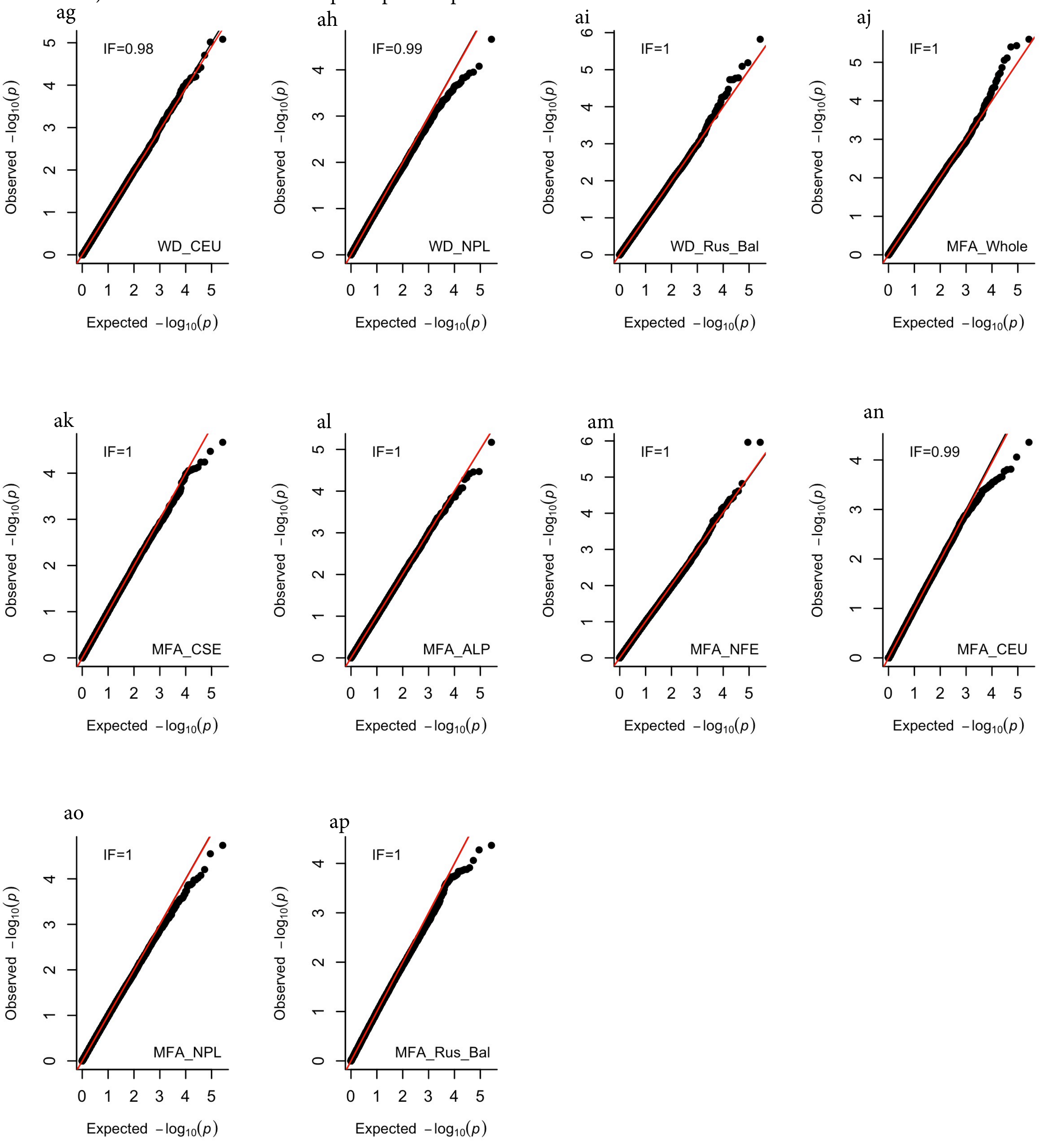

D) CMLM with no principal component as a covariate

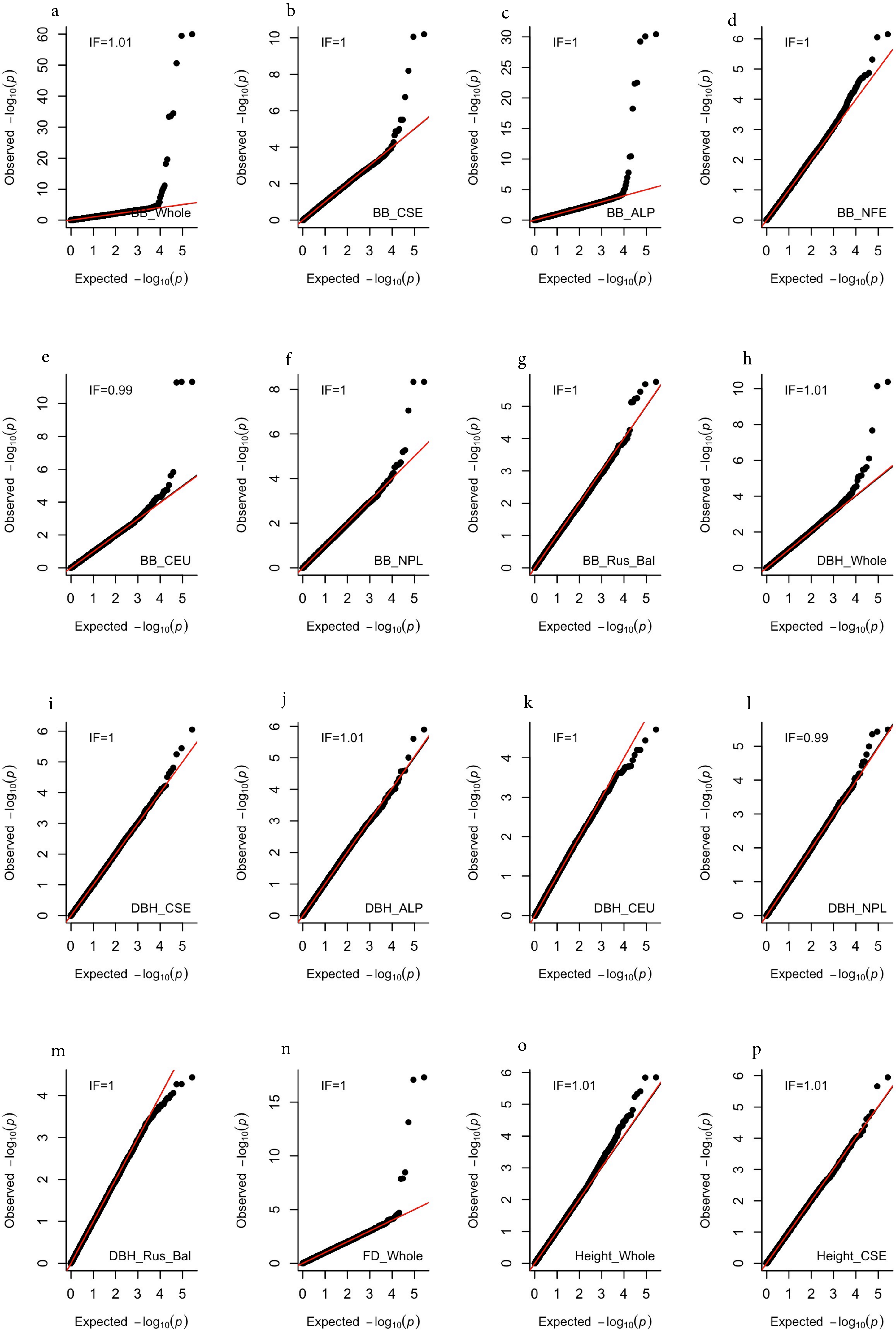

D) CMLM with no principal component as a covariate

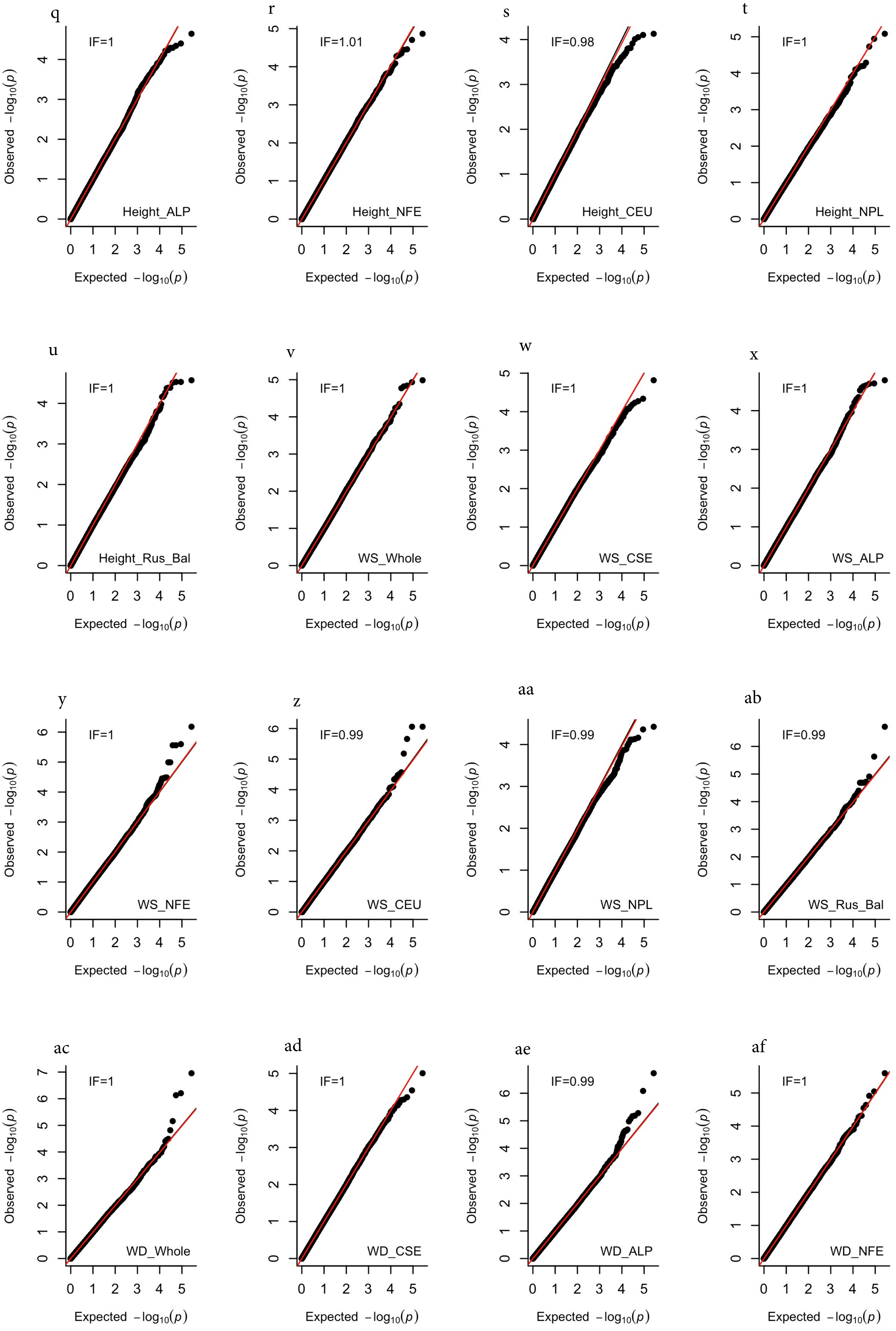

D) CMLM with no principal component as a covariate

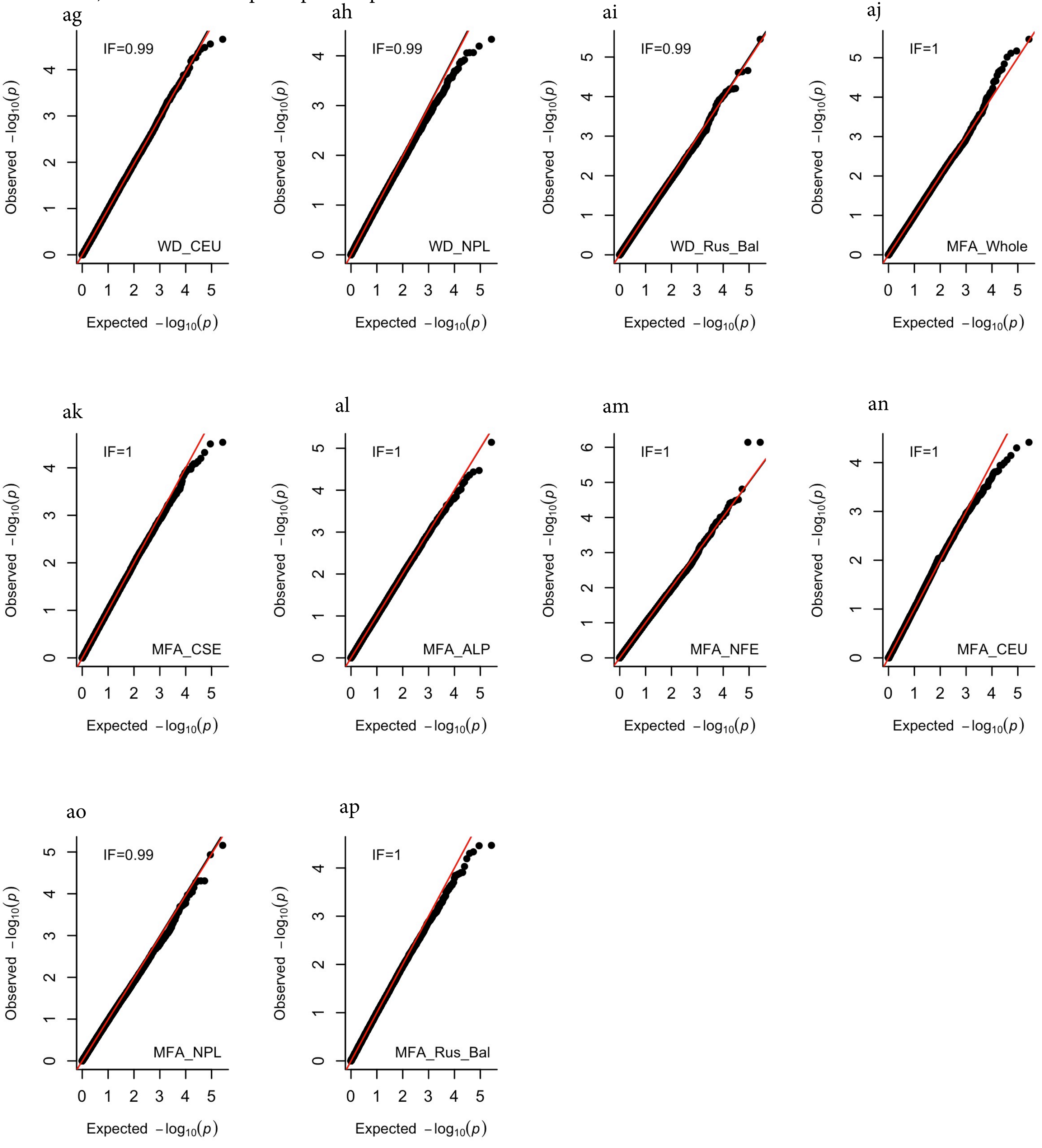

Figure S6 Quantile-Quantile (QQ) plots and genomic inflation factors (IF) for all genome-wide associations (GWAs). The red line means inflation value. A) QQ plots using BLINK with one principal component as a covariate for all seven traits including budburst stage (BB) for a) the whole population (BB_Whole), b) central and southern Sweden (BB_CSE), c) Alpine (BB_ALP), d) Fennoscandian (BB_NFE), e) Central Europe (BB_CEU), f) Northern Poland (BB_NPL), and g) Russia-Baltic (BB_Rus-Bal), h–m) diameter at breast height (DBH), n) frost damage (FD), o–u) tree height (Height), v–ab) wood stiffness (WS), ac–ai) wood density (WD), and aj-ap) microfibril angle (MFA) for different genetic clusters as for BB; B) QQ plots using BLINK with no principal component as a covariate for all the traits and genetics clusters as in A); C) QQ plots using CLMM method without two or three principal components as covariates for all the traits and genetics clusters as in A); D) QQ plots using CLMM method with no principal component as a covariate for all the traits and genetic clusters as in A).

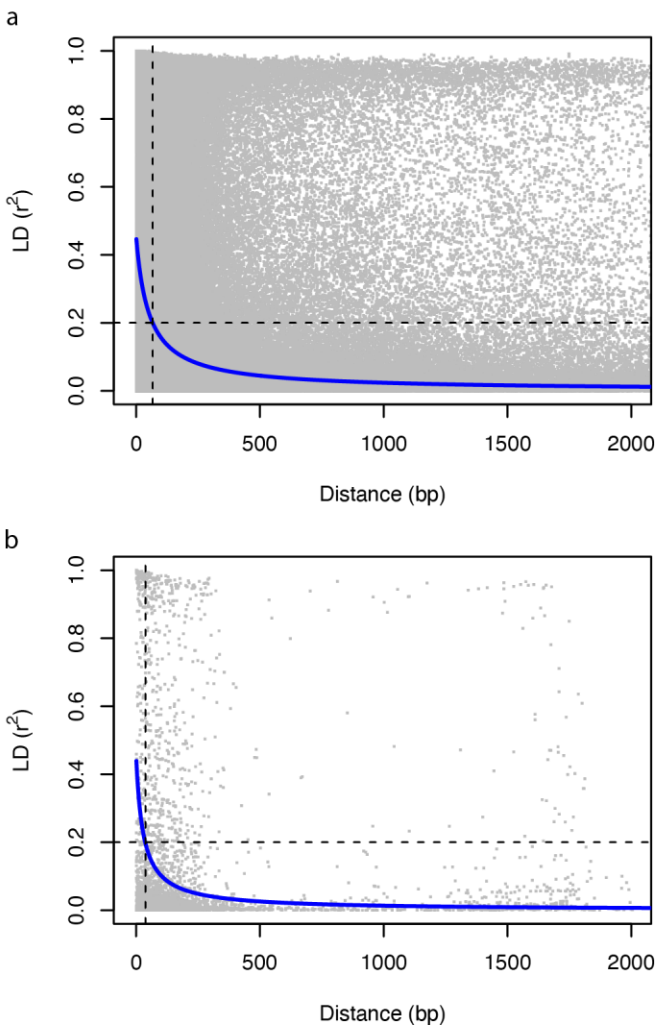

Figure S7 Decay of linkage disequilibrium in exome capture genome: a) Decay of linkage disequilibrium (LD) across all SNPs within all contigs. b) Decay of LD across SNPs within all associated contigs. The x-axis is the distance in nucleotides; y-axis the correlations coefficients (r^2) between nucleotides sites.

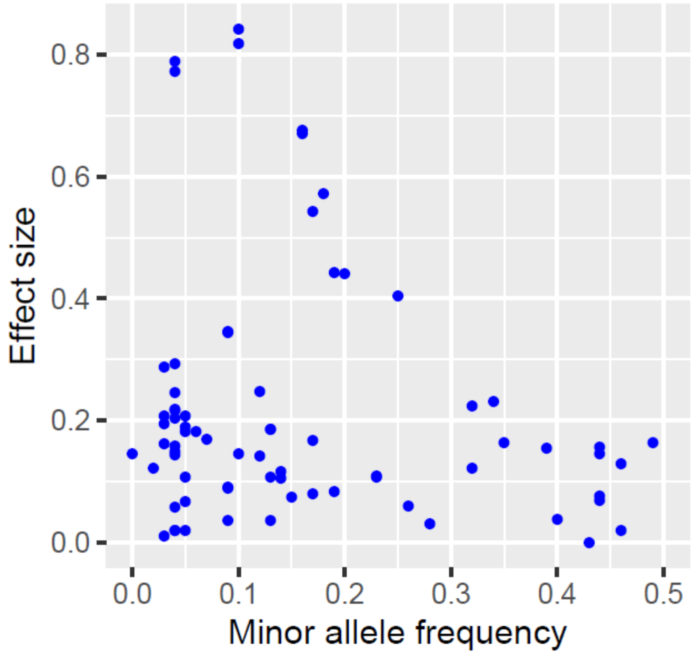

Figure S8 The relationship between effect size and minor allele frequency for all SNPs associated with budburst stage (BB).

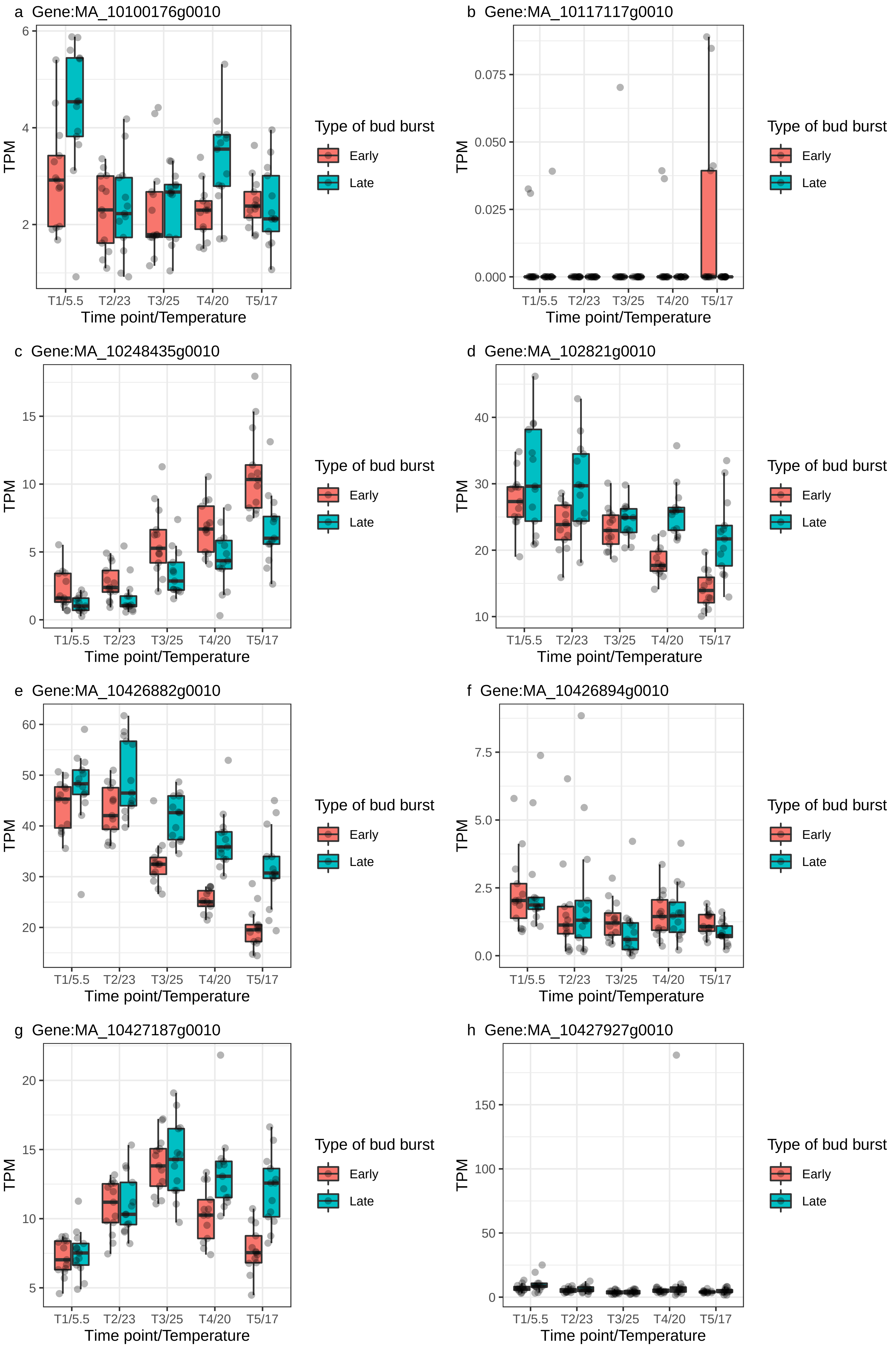
a Gene:MA_10100176g0010
b Gene:MA_10117117g0010
c Gene:MA_10248435g0010
d Gene:MA_102821g0010
e Gene:MA_10426882g0010
f Gene:MA_10426894g0010
g Gene:MA_10427187g0010
h Gene:MA_10427927g0010
TPM
Time point/Temperature
T1/5.5
T2/23
T3/25
T4/20
T5/17
Type of bud burst
Early
Late

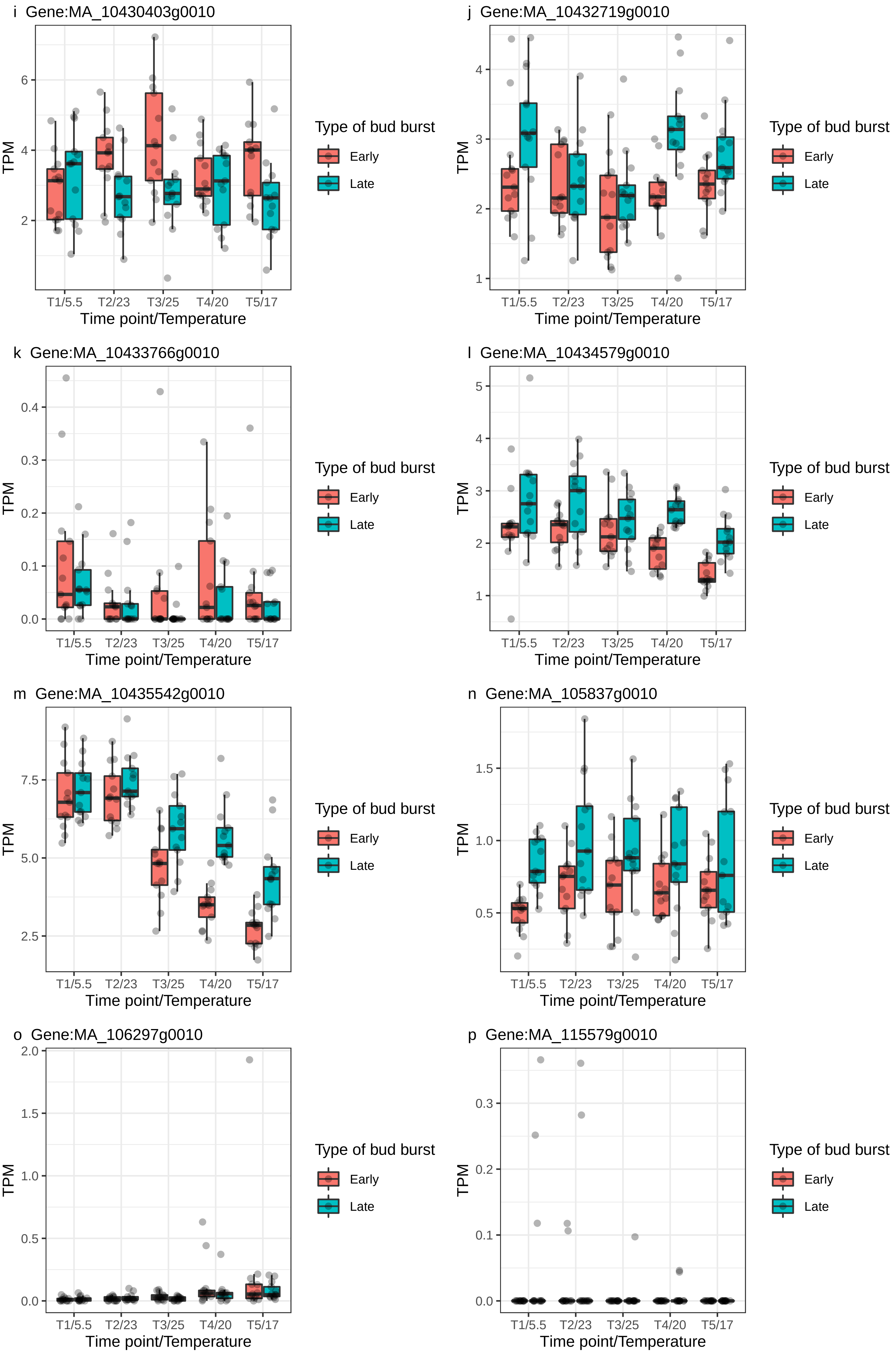
i Gene:MA_10430403g0010
j Gene:MA_10432719g0010
k Gene:MA_10433766g0010
l Gene:MA_10434579g0010
m Gene:MA_10435542g0010
n Gene:MA_105837g0010
o Gene:MA_106297g0010
p Gene:MA_115579g0010
TPM
Time point/Temperature
T1/5.5
T2/23
T3/25
T4/20
T5/17
Type of bud burst
Early
Late

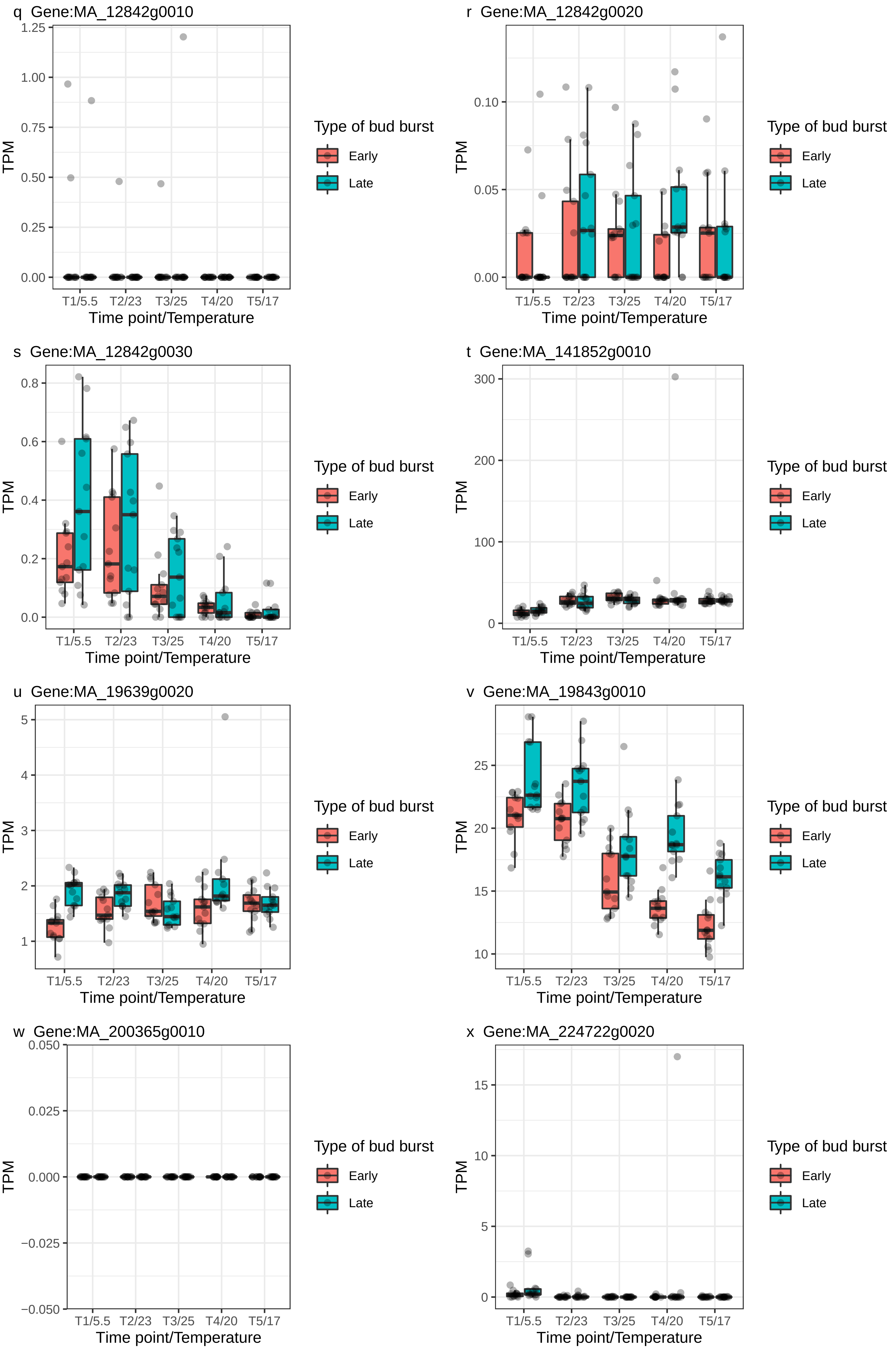
q Gene:MA_12842g0010
r Gene:MA_12842g0020
s Gene:MA_12842g0030
t Gene:MA_141852g0010
u Gene:MA_19639g0020
v Gene:MA_19843g0010
w Gene:MA_200365g0010
x Gene:MA_224722g0020
TPM
T1/5.5
T2/23
T3/25
T4/20
T5/17
Time point/Temperature
Type of bud burst
Early
Late

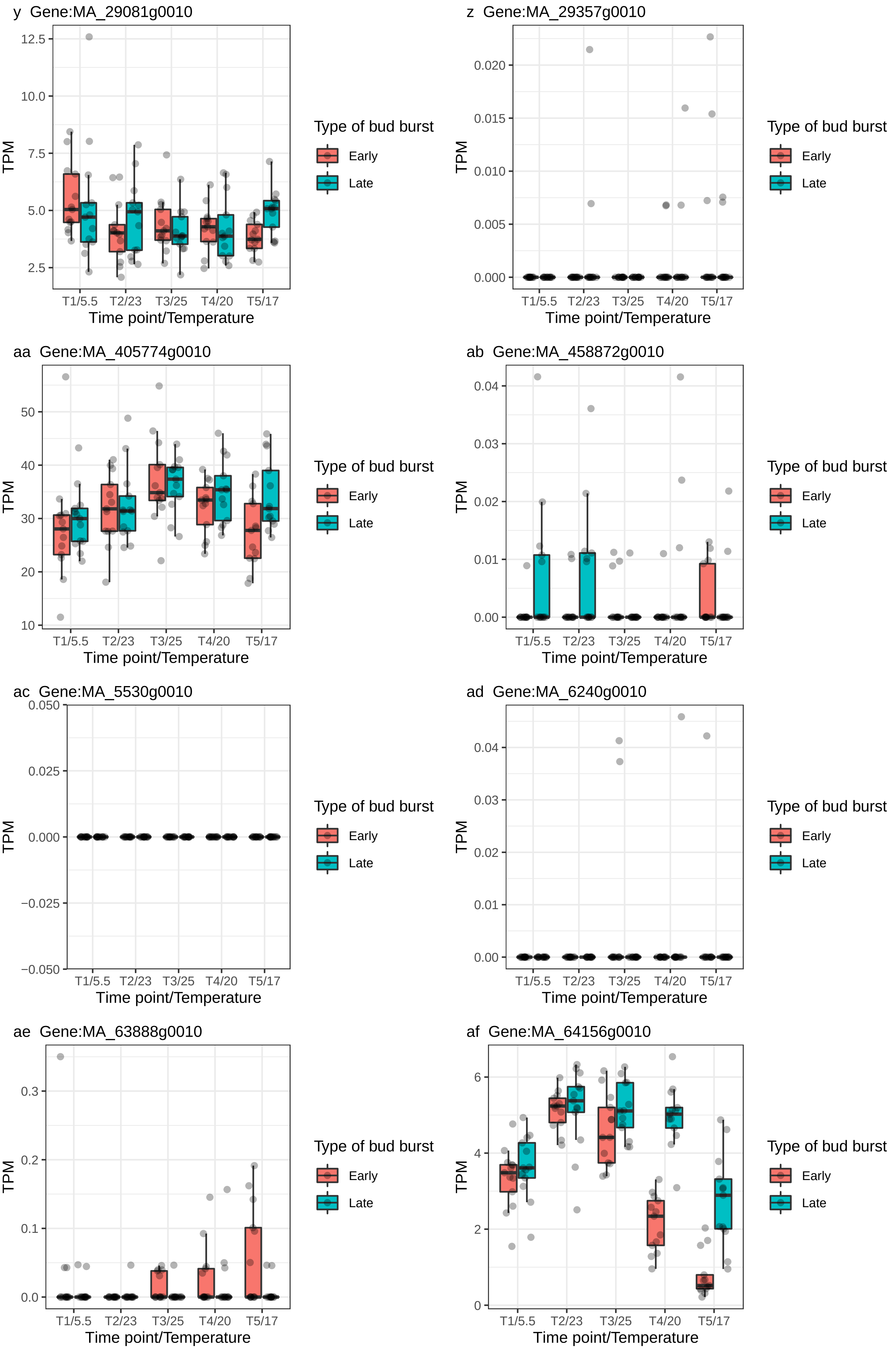
y Gene:MA_29081g0010
z Gene:MA_29357g0010
aa Gene:MA_405774g0010
ab Gene:MA_458872g0010
ac Gene:MA_5530g0010
ad Gene:MA_6240g0010
ae Gene:MA_63888g0010
af Gene:MA_64156g0010
TPM
Time point/Temperature
T1/5.5
T2/23
T3/25
T4/20
T5/17
Type of bud burst
Early
Late

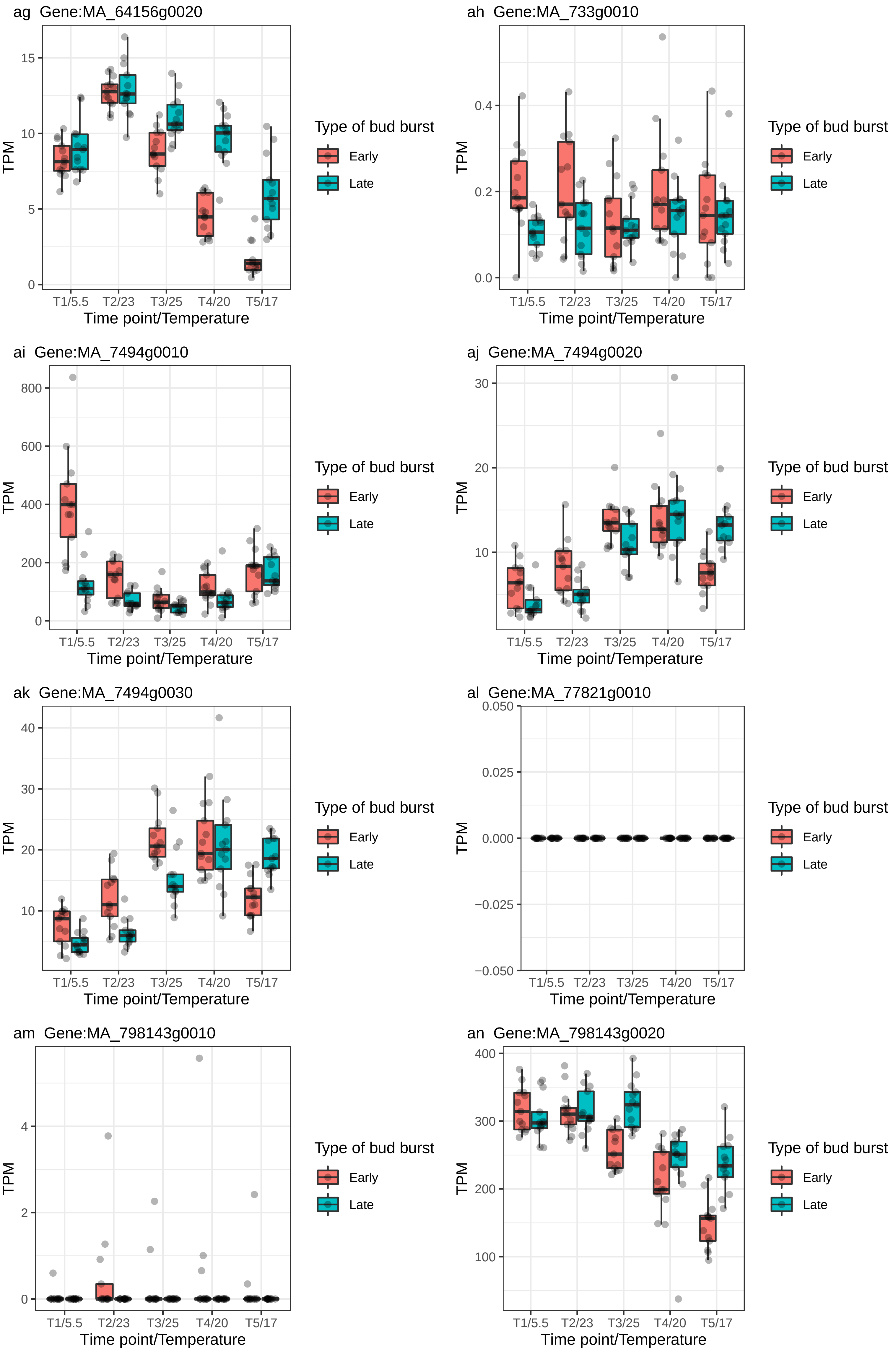
ag Gene:MA_64156g0020
ah Gene:MA_733g0010
ai Gene:MA_7494g0010
aj Gene:MA_7494g0020
ak Gene:MA_7494g0030
al Gene:MA_77821g0010
am Gene:MA_798143g0010
an Gene:MA_798143g0020
TPM
Time point/Temperature
T1/5.5
T2/23
T3/25
T4/20
T5/17
Type of bud burst
Early
Late

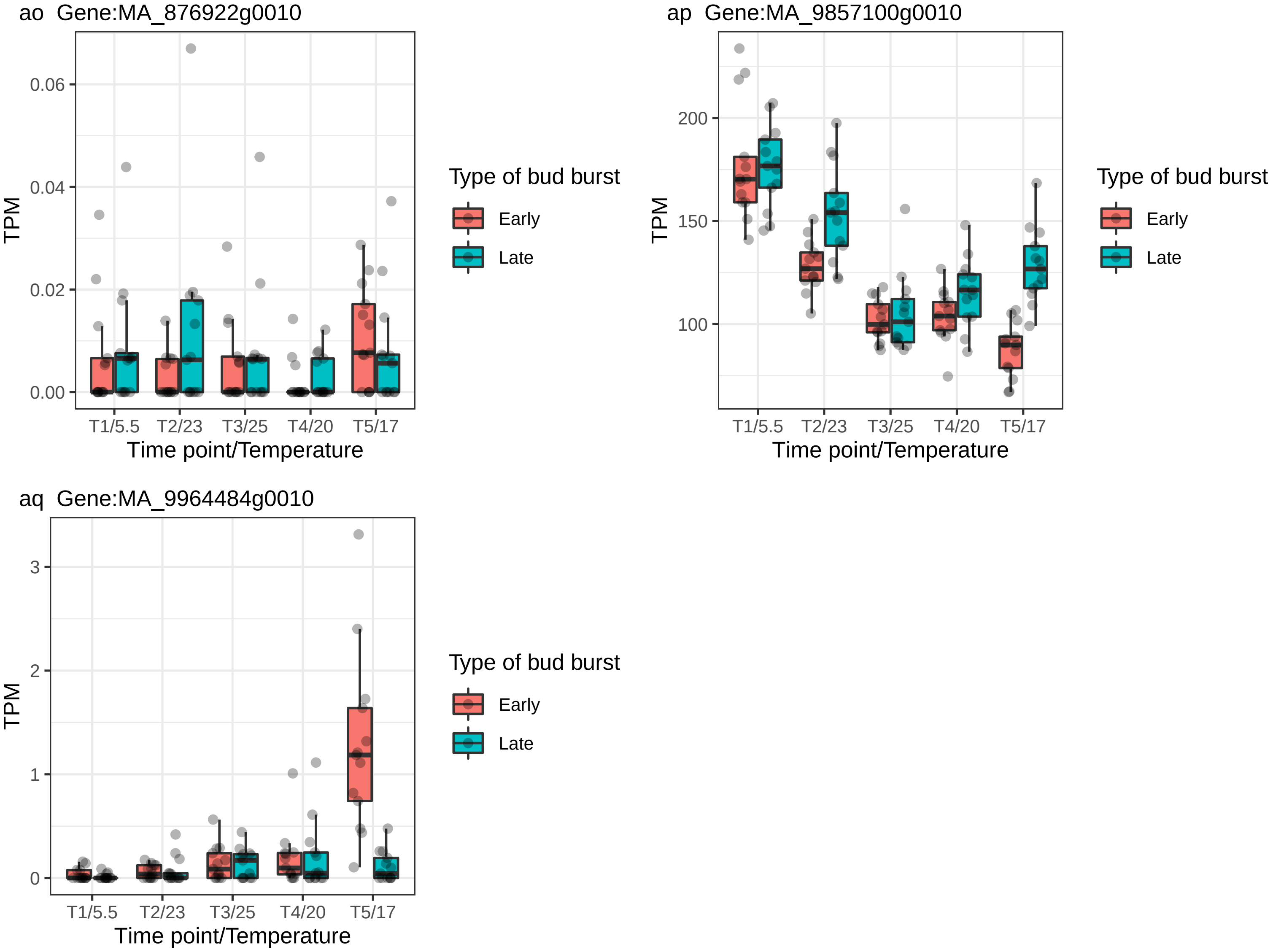

Figure S9 The expression difference in TPM (Transcripts Per Kilobase Million) of 43 genes related to budburst stage and frost damage. a) Expression difference of gene MA_10100176g0010, in buds with early budburst and late budburst in five sampling times (T1–T5) and also the temperatures from 5.5 to 25 ℃. b–aq) the expression difference of the rest of 42 genes in buds with early budburst and late budburst in five sampling times (T1–T5) and also the temperatures from 5.5 to 25 ℃.

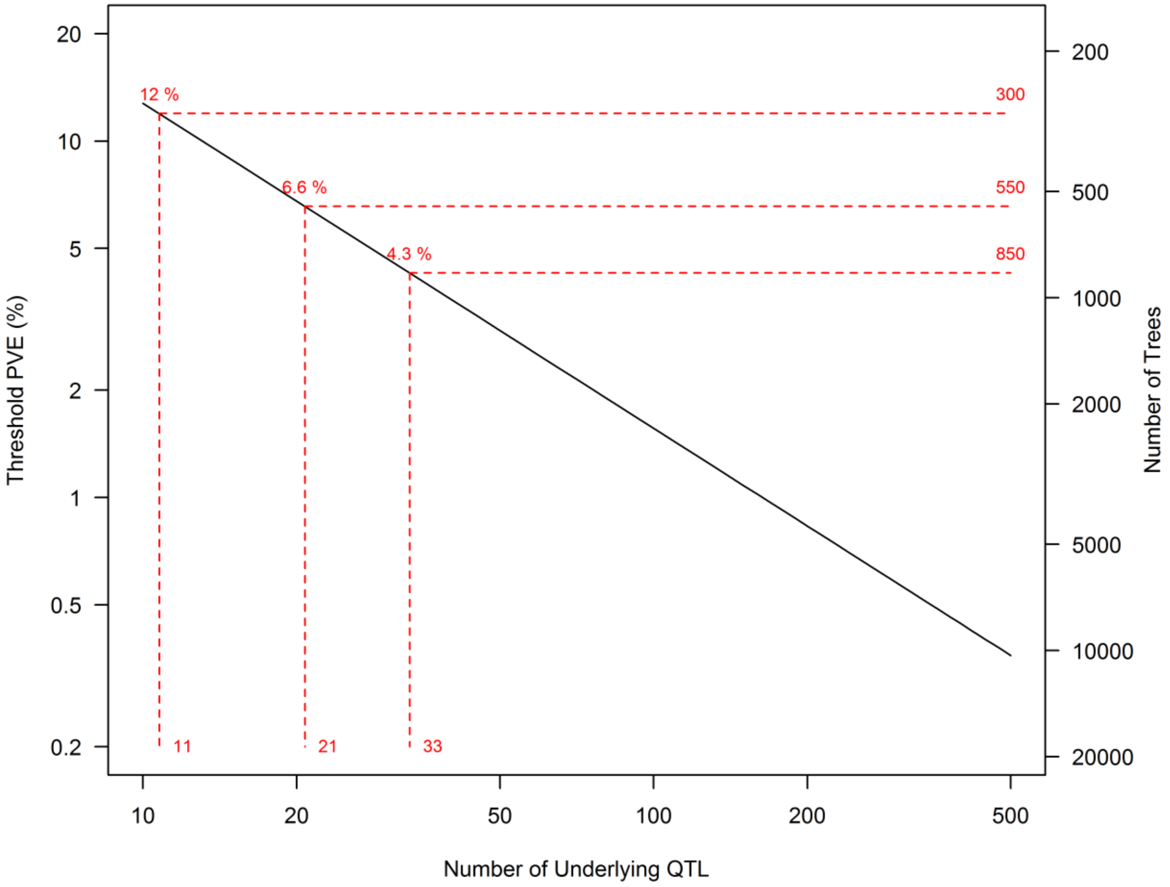

Figure S10 Relationship between the underlying number of QTLs and both required tree number and threshold of the percentage of variance explained (PVE) detectable for individual QTLs under 80% power and a significance level of 1×10-7

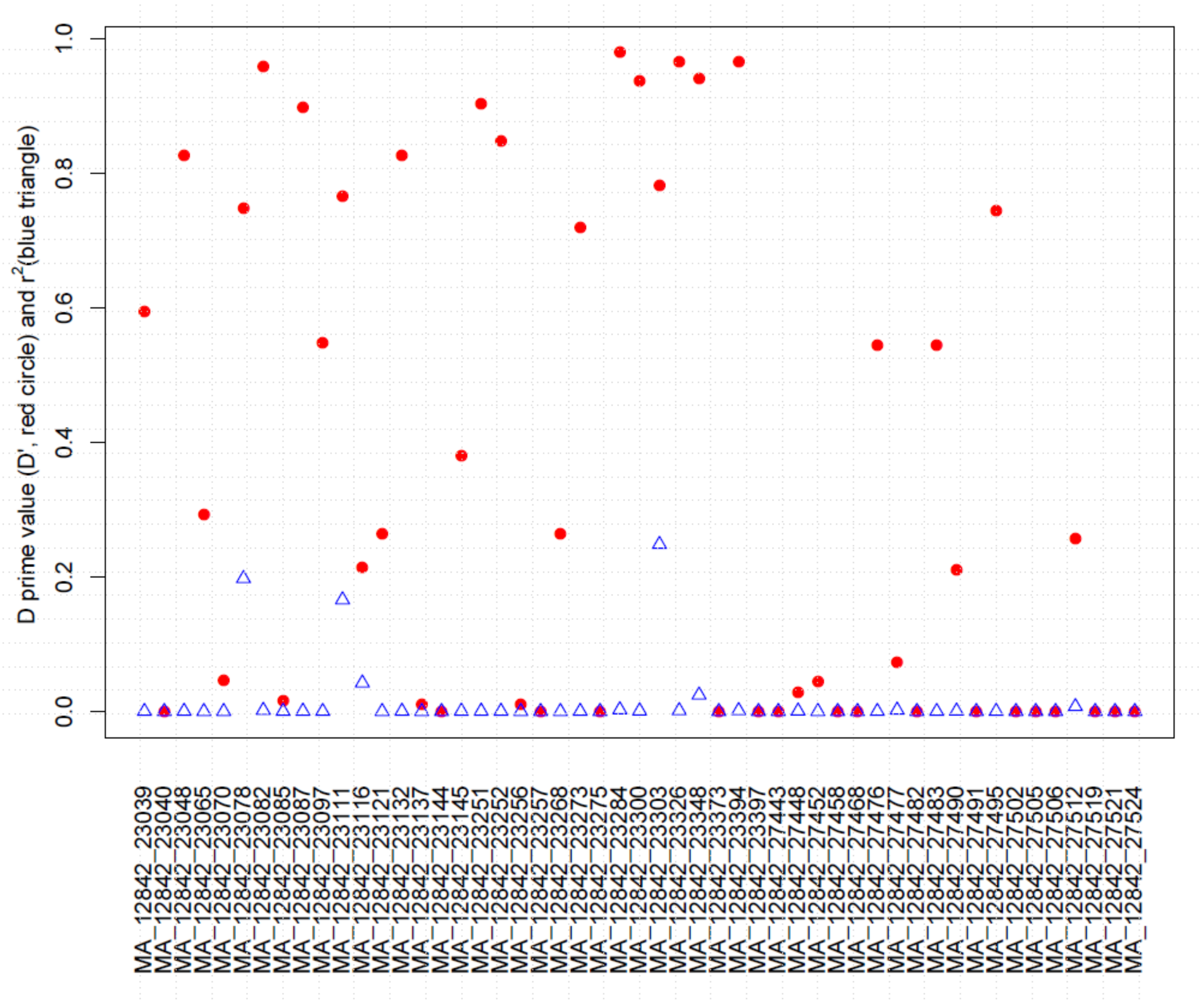

Figure S11 Linkage disequilibrium (LDs) between SNPs in the gene model MA_12842g100030 and the significant SNP MA_12842_2490 in contig MA_12842.

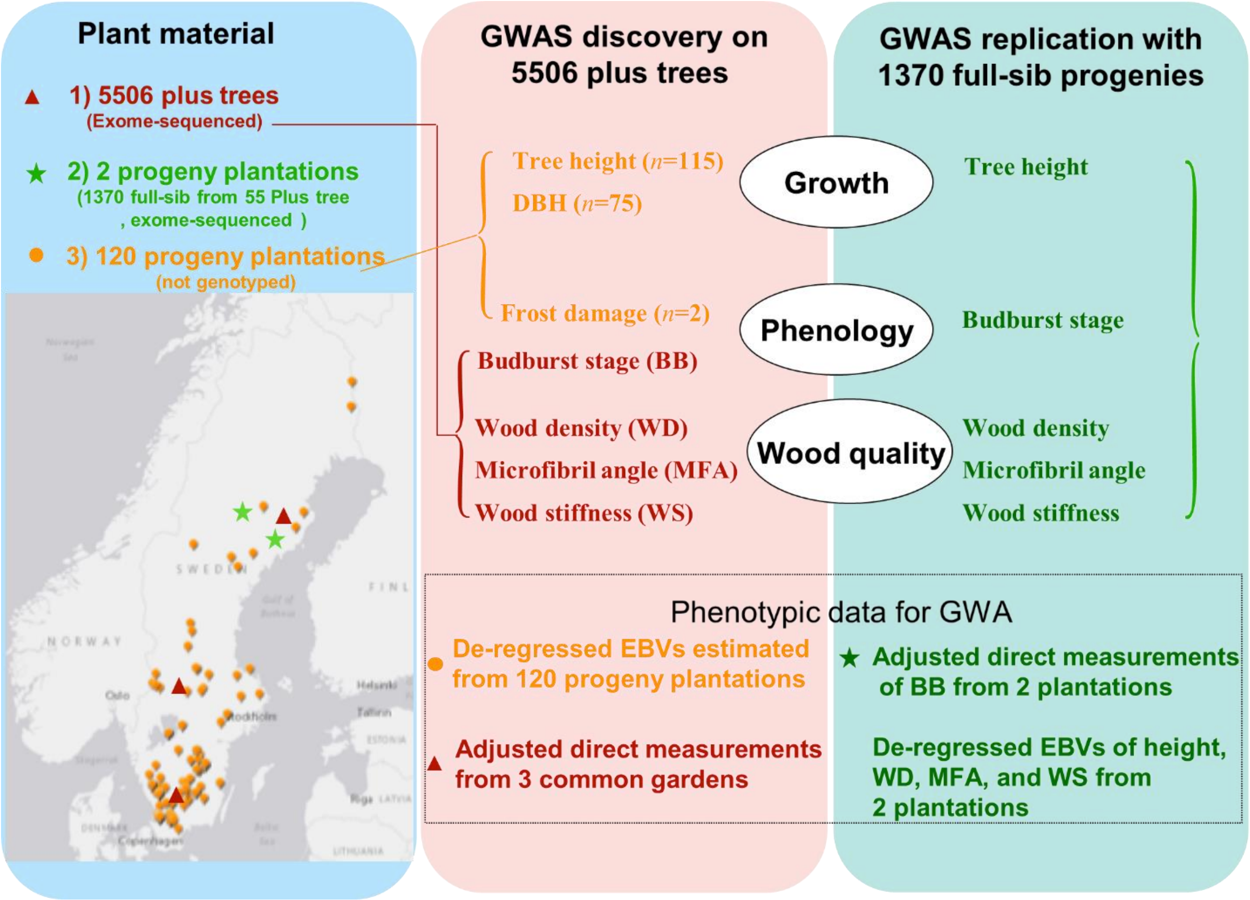

Figure S12 Left side of Figure: three sets of Norway spruce plant materials used in our GWAS: 1) 5506 plus trees drafted in three common gardens (dark red triangle), 2) 1370 full-sib progenies planted in two full-sib field plantations (green star), and 3) 120 progeny plantations (orange dots). Middle of the figure, 1) we showed the traits phenotyped in how many numbers of field plantations and common gardens (n). De-regressed breeding values (EBVs) estimated from 120 progeny plantations for height, DBH, and frost damage (FD) were used as the adjusted phenotypic values for GWA. The adjusted direct measurement of budburst stage (BB), wood density (WD), microfibril angle (MFA), and wood stiffness (WS) were used as adjusted phenotypic values for GWA The right side of Figure: the traits were measured and adjusted phenotypic values for wood quality traits, and de-regressed EBVs of height, were used for causal variants validation in two full-sib plantations.

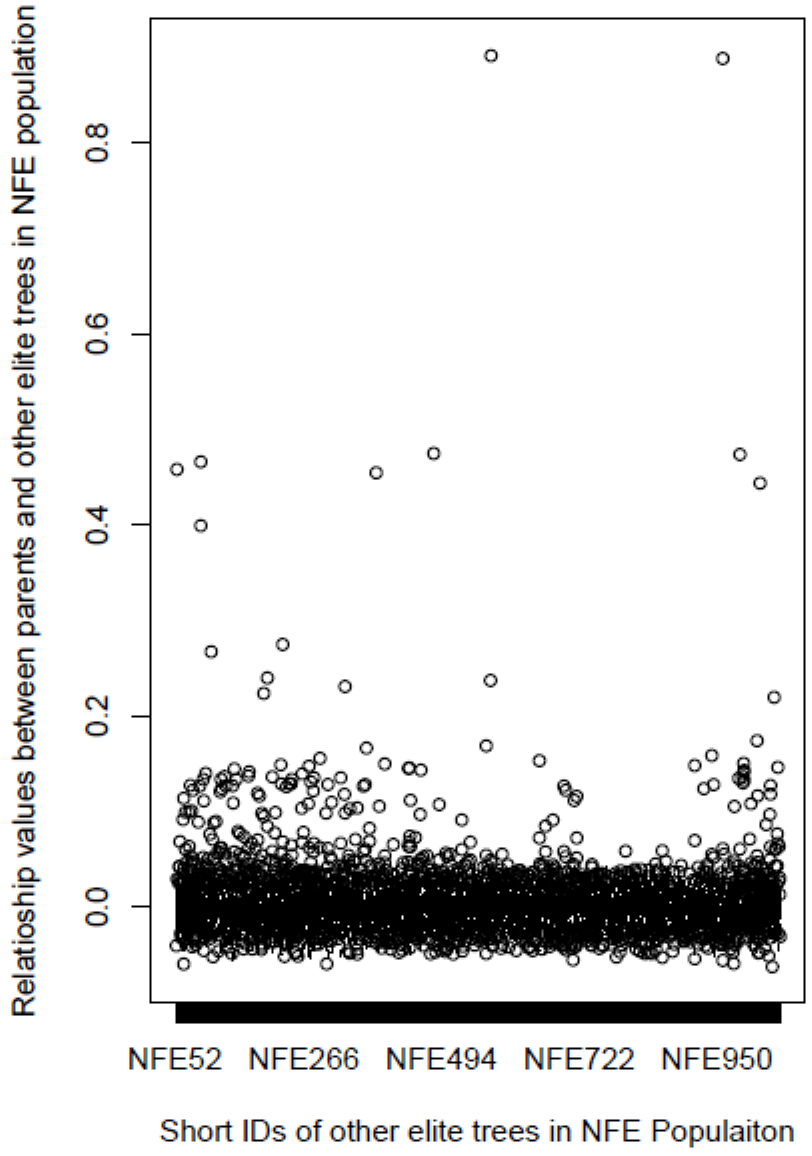

Figure S13 Relationship values between parents and other elite trees in the Fennoscandia group (NFE) population.
