# Supplementary material for: Leveraging breeding programs and genomic data in Norway spruce (Picea abies L. Karst) for GWAS analysis
Source: Genome Biol. 2021 Jun 13;22:179. doi: 10.1186/s13059-021-02392-1 (PMC8201819; doi:10.1186/s13059-021-02392-1)
Supplement: Supplementary file 3 — Additional file 3: Supplementary Methods. [file 13059_2021_2392_MOESM3_ESM.pdf]

Supplymentary methods

Adjusting environment effects using spatial model for tree height and DBH in field plantations

All available individual data from 120 field experimental plantations (Table S1) in Sweden were used to adjust environment effect for tree height and diameter at breast height (DBH) in each field plantation. To adjust the environmental effects for each trait, a mixed linear model was fitted using a restricted maximum-likelihood method for each plantation in ASReml 4.1 [1]. The model included fixed effects for blocks and the random effect of plus trees in a complete nested model. In addition, the model included random row and column residual effects with a first-order autoregressive covariance structure to exclude the spatial environmental effects of field variation [2].

The general mixed linear model can be expressed in matrix form as:

$$\boldsymbol{y} = \mathbf{X}\boldsymbol{b} + \mathbf{Z}\boldsymbol{u} + e \qquad [1]$$

where $\boldsymbol{y}$ is a vector of measured data, $\boldsymbol{b}$ is a vector of fixed effects with corresponding design matrix noted as X, $\boldsymbol{u}$ is a vector of random effects with corresponding design matrix $\mathbf{Z}$, $\boldsymbol{e}$ is a vector of residuals. Fixed and random effects solutions are obtained by solving the linear mixed model equations:

$$\begin{bmatrix} \mathbf{X}'\mathbf{R}^{-1}\mathbf{X} & \mathbf{X}'\mathbf{R}^{-1}\mathbf{Z} \\ \mathbf{Z}'\mathbf{R}^{-1}\mathbf{X} & \mathbf{Z}'\mathbf{R}^{-1}\mathbf{Z} + \mathbf{G}^{-1} \end{bmatrix} \begin{bmatrix} \hat{\boldsymbol{b}} \\ \hat{\boldsymbol{u}} \end{bmatrix} = \begin{bmatrix} \mathbf{X}'\mathbf{R}^{-1}\boldsymbol{y} \\ \mathbf{Z}'\mathbf{R}^{-1}\boldsymbol{y} \end{bmatrix} \qquad [2]$$

where **R** is the variance-covariance matrix of the residuals and **G** is the direct sum of the variance-covariance matrices for each of the random effects. Residuals are assumed to be independent and normal distributed and **R** is denoted as $\sigma_e^2\mathbf{I}$ in the base model. Here, spatial analysis allows **R** to have a different structure based on a decomposition of $\boldsymbol{e}$ into spatially dependent ($\boldsymbol{\xi}$) and independent ($\boldsymbol{\eta}$) residuals (nugget effect). The spatially dependent ($\boldsymbol{\xi}$) residuals are modelled using a covariance structure that assumes a separable first-order autoregressive process in rows and columns, for which the **R** matrix is

$$\mathbf{R} = \sigma_\xi^2\,[\mathrm{AR1}(\mathrm{p_{col}}) \otimes \mathrm{AR1}(\mathrm{p_{row}})] + \sigma_\eta^2\boldsymbol{I} \qquad [3]$$

where σ_ξ^2 is the spatial dependent residual variance, σ_η^2 is the independent residual variance, I is an identity matrix, $\otimes$ is a direct product (the Kronecker product) for two matrices and AR1(p_{col}) and AR1(p_{row}) represent a first-order autoregressive correlation matrix in column and row directions, respectively.

Estimates of variance components for four datasets

In this study, four datasets were used to estimate the variance components and genetic parameters:

1) In three common gardens, variance components of budburst stage (BB) and wood quality traits (Density, microfibril angle, and wood stiffness) were estimated using the following general mixed linear model:

$$y = X\beta + Za + e \qquad [4]$$

Where y is a vector of the phenotypic observations of a single trait in each common garden. β is a vector of fixed effects including the grand mean and block effects, a and e are vectors of random additive and random error effects, respectively, and X and Z are the incidence matrices. Random additive effects in vector assuming $a \sim N(0, \sigma_u^2 K)$, e is the vector of residual effects assuming $e \sim N(0, \sigma_e^2 I)$. Here, K is a pedigree-based relationship matrix (*A*), but those plus trees are unrelated, thus A=*I*. Variance components of repeatabilities for budburst stage (BB), wood density, microfibril angle, and wood stiffness were estimated by the question [4].

2) In two half-sib progeny field plantations (F1215 and F1150, Table S5), variance components of tree height and diameter at breast height (DBH) were estimated using the following general mixed linear model:

$$y = X\beta + Wb + Zf + e \quad [5]$$

Where $\boldsymbol{y}$ is a vector of phenotypic observations of a single trait; β is a vector of fixed effects, including a grand mean, site, post-block within site effects, b is a vector of random incomplete block effects, f is a vector of random effects, including family and family by site interaction effects. *X*, *W*, and *Z* are incidence matrices for β, b, and f, respectively. The random effects (*f*) assume to follow $f \sim N(0, \begin{bmatrix} \sigma_f^2 I_{n12} & & \\ & \sigma_{fs1}^2 I_{n1} & \\ & & \sigma_{fs2}^2 I_{n2} \end{bmatrix})$, where family variance σ_f^2 is the ¼ additive variance, σ_{fs1}^2 and σ_{fs2}^2 are the variances of family-by-site 1 and family-by-site 2 interaction effects, respectively. The residual e was assumed to follow $e \sim N(0, \begin{bmatrix} I_{n1}\sigma_{e1}^2 & \\ & I_{n2}\sigma_{e2}^2 \end{bmatrix})$, where σ_{e1}^2 and σ_{e2}^2 are the residual variances for site 1 and site 2, I_{n12}, I_{n1} and I_{n2} are identity matrices, n_{12}, n1 and n2 are the number of individuals for two sites, site 1 and site 2, respectively. When we estimated the heritability for joint-sites, variance structures for the family by site interaction in μ and e are assumed as homogeneous.

3) In the two half-sib progeny field plantations, variance components of frost damage (FD) were estimated using a generalized linear mixed model. Due to the FD was scored as a binary variable (0 or 1) and had a binomial (η, π) distribution where η is the conditional function g(u) and u is the conditional mean, π is the probability of a tree that did not be affected. In this study, the variance component of FD and EBVs were estimated using the following generalized mixed linear model:

$$\eta = log[\pi/(1-\pi)] = X\beta + Wb + Zf + e \quad [6]$$

Where all the parameters explained are similar in equation 5 as using the same material.

4) In two full-sib progeny field plantation used for variants validation, variance components of tree height, budburst stage, wood density, microfiril angle, and wood stiffness were estimated using the following general mixed linear model:

$y = X\beta + \mathrm{W}b + \mathrm{Z}\mu + e$ [6]

Where $\boldsymbol{y}$ is a vector of phenotypic observations of a single trait; β is a vector of fixed effects, including a grand mean and site effect, b is a vector of random post-block within site effect, μ is a vector of random site by additive effects of individuals. X, W, and Z are incidence matrices for β, b, and μ, respectively. The more details of the two field trials could be referred to two published papers [3, 4]. The random additive effects (u) assume to follow $u \sim N(0, A\begin{bmatrix}\sigma_{a1}^2 & \sigma_{12} \\ \sigma_{12} & \sigma_{a2}^2\end{bmatrix})$, where A is the additive genetic relationship matrix, σ_{a1}^2 and σ_{a2}^2 are the additive genetic variances for site 1 and site 2, respectively, σ_{12} is additive genetic covariance between site 1 and site 2. The residual e was assumed to follow $e \sim N(0, \begin{bmatrix}I_{n1}\sigma_{e1}^2 & \\ & I_{n2}\sigma_{e2}^2\end{bmatrix})$, where σ_{e1}^2 and σ_{e2}^2 are the residual variances for site 1 and site 2, I_{n1} and I_{n2} are identity matrices, n1 and n2 are the number of individuals in each site. When we estimated the heritability for joint-sites, variance structures for family by site interaction in μ and e are assumed as homogeneous and family effect was added in the model in order to dissect G×E.

5) For all trait from the whole plus trees population, variance components were estimated using the following general mixed linear model

$y' = X\beta + Za + e$ **[7]**

Where y' is a vector of one of seven traits (i.e. the de-regressed breeding values of tree height, DBH, and FD or adjusted phenotypic values of three wood quality traits or BB). β is a vector of fixed effects including a grand mean, a and e are vectors of random additive and random error effects, respectively, and X and Z are the incidence matrices. random effects (additive) in vector assuming $a \sim N(0, \sigma_u^2 K)$, e is the vector of residual effects assuming $e \sim N(0, \sigma_e^2 I)$. Here, K is the genomic relationship matrix (GRM). The later will be specified the difference between A and G.

1) ABLUP

The ABLUP is the traditional method that utilizes a pedigree relationship matrix (A) to predict the EBV. For ABLUP the vector of random additive effect (a) in equation [4] is assumed to follow a normal distribution $a \sim N(0, A\sigma_a^2)$, where σ_a^2 is the additive genetic variance. The residual vector e is assumed as $e \sim N(0, I\sigma_e^2)$, where I is the identity matrix.

2) GBLUP

The GBLUP model is the same as ABLUP, with the only difference being that the genomic relationship matrix (GRM) replaces the A matrix. The GRM is calculated as $GRM = \frac{(M-P)(M-P)^T}{2\sum_{i=1}^{q} p_i(1-p_i)}$, where M is the matrix of samples with SNPs encoded as 0, 1, 2 (i.e. the number of minor alleles), P is the matrix of allele frequencies with the ith column given by

$2(p_i - 0.5)$, where p_i is the observed allele frequency of all genotyped samples. In GBLUP, the random additive effect (a) in equation [4] is assumed to follow $a \sim N(0, I_p\sigma_g^2)$, where σ_g^2 is the genomic-based genetic variance. The inverse of G matrix was estimated using write.realtionshipMatrix function in synbreed package in R.

Estimate of heritability

In this study, we estimated two types of narrow-sense heritabilities: 1) pedigree-based narrow-sense heritability (h_a^2) and 2) SNP-based narrow-sense heritability (h_g^2). The $h_a^2$and h_g^2 were calculated as

$$h_a^2 = \frac{\sigma_a^2}{\sigma_{pa}^2};\ h_g^2 = \frac{\sigma_g^2}{\sigma_{pg}^2}$$

respectively, where σ_a^2 is the pedigree-based additive variance estimated from ABLUP, while σ_g^2 is the marker-based additive variance estimated from GBLUP. σ_{pa}^2 and σ_{pg}^2 are phenotypic variances for pedigree-based and marker-based models, respectively. Due to using the de-regressed breeding values of tree height, DBH, and FD as the phenotypic value y' in equation [7], the SNP-based narrow-sense heritability (H_g^2) for height, DBH and frost damage (FD) was calculated as

$H_g^2 = h_a^2 * h_g^2,$

Where h_a^2 is the pedigree-based narrow-sense heritability estimated from the two half-sib progeny plantations. Here, because of lacking ca. estimates of pedigree-based narrow-sense heritability in whole plus tree population, we just used the ca. estimate as a surrogate.

Estimate of repeatability

Repeatabilities (p) of three wood quality traits and BB were estimated as

$$p = \frac{\sigma_c^2}{\sigma_c^2 + \sigma_e^2}$$

Where σ_c^2 is the clone variance estimated from equation [4], σ_e^2 is the residual variance estimated from equation 1 using ASReml 4.0.

Estimates of genetic correlation

Bivariate models were used to estimate the pedigree-based and SNPs-based genetic correlations. The bivariate model is an extended equation from the single trait model for each dataset.

Pedigree-based genetic correlation between pairs of traits was estimated as:

$$\mathrm{r}_{(\mathrm{x,y})} = \frac{\widehat{\mathrm{Cov}}_{(\mathrm{x,y})}}{\sqrt{\hat{\sigma}^2_{(\mathrm{x})} \times \hat{\sigma}^2_{(\mathrm{y})}}}$$

where $\hat{\sigma}^2_{(x)}$ and $\hat{\sigma}^2_{(y)}$ are the estimated phenotypic and genetic variances for traits x and y, respectively, $\widehat{\mathrm{Cov}}_{(x,y)}$ is the estimated phenotypic and genetic covariance between traits x and y. Their standard errors were estimated using the Taylor series expansion method in ASREML 4.0 [1] The SNP-based genetic correlation between pairs of traits was estimated as pedigree-based genetic correlation. Only the variance and covariance were estimated using different relationship matrices.

References

1. Gilmour AR, Gogel BJ, Cullis BR, Welham SJ, Thompson R. ASReml user guide release 4.1. In. Hemel Hempstead, UK: VSN International Ltd; 2015.
2. Chen Z-Q, Helmersson A, Westin J, Karlsson B, Wu HX. Efficiency of using spatial analysis for Norway spruce progeny tests in Sweden. Ann For Sci. 2017; 75(1):2.
3. Chen Z-Q, Baison J, Pan J, Westin J, Garcia-Gil MR, Wu HX. Increased prediction ability in Norway spruce trials using a marker x environment interaction and non-additive genomic selection model. J Hered. 2019; 110(7):830–843.
4. Chen Z-Q, Baison J, Pan J, Karlsson B, Andersson B, Westin J, et al. Accuracy of genomic selection for growth and wood quality traits in two control-pollinated progeny trials using exome capture as the genotyping platform in Norway spruce. BMC Genomics. 2019; 19(1):946.
