# Supplementary material for: Leveraging breeding programs and genomic data in Norway spruce (Picea abies L. Karst) for GWAS analysis
Source: Genome Biol. 2021 Jun 13;22:179. doi: 10.1186/s13059-021-02392-1 (PMC8201819; doi:10.1186/s13059-021-02392-1)
Supplement: Supplementary file 4 — Additional file 4. Review history. [file 13059_2021_2392_MOESM4_ESM.docx]

Review History

**First round of review**

**Reviewer 1**

**Are you able to assess all statistics in the manuscript, including the appropriateness of statistical tests used?**

**Yes.**

**Comments to author:**

Manuscript of " Leveraging breeding program and genomic data in Norway spruce (Picea abies L.Karst): a GWAS on adaptive and wood quality traits" (GBIO-D-21-00035) performed GWAS to reveal candidate genes related to 5 traits combining different phenotypic data. This study takes advantages of phenotypic data from different breeding programs, which was quite meaningful, especially for perennial tree study. The results will provide reference for Norway spruce genomic study. But more experiments and analysis should be provided and improved to support the key findings.

The conclusion seems too primary. The candidate genes identified were not validated and it is a long way to perform gene editing.

The comments are listed below:

1. Although the limitation of reference genome for Norway spruce, too little SNPs were identified using exon-sequencing for more than 4000 individuals. Is it possible to get much more genotypes? Such as low depth sequencing. If so, the GWAS result would be much more reliable. Because many traits were not directly associated SNPs in exon.

2. Why the author used maf value 0.03?

3. In GWAS, two models were used (BLINK and CMLM), why selected these two? As we know, the CMLM would ignore much more related SNPs, and only kept the most significant signal, which might reduce the number or important loci that might be related to trait.

4. Why the author compare the results of BLINK and CMLM? Do you want to select a much better model?

5. Actually, the authors performed GWAS using different population or clusters and different models to idenfify QTLs. But how to make sure which results were much accurate. Too many ways would increase the false positives. How to deal with such problem?

6. Page10 line 33, what is the possible reason for the higher phenotypic variation of BB.

7. Page10 line 45, why delete FD and DBH?

8. In GWAS, what is the threshold of P-value, how do you determine it?

9. There are SNPs in gene intron region, such as page 11 line 33-40. Such SNPs can not change gene function, so how they affect the phenotypes? Do they affect the gene expression? Much more experiments should be carried out.

10. Page 11 Line 45, the word "inside gene", which region? Should be declared clearly.

11. When selecting candidate genes based on significant SNPs, have you considered the LD? Or just selected the gene containing SNPs. As we know, many traits were not caused by a single SNP, but large genome variations.

**Reviewer 2**

**Are you able to assess all statistics in the manuscript, including the appropriateness of statistical tests used?**

No, I do not feel adequately qualified to assess the statistics.

**Comments to author:**

This manuscript reports the most extensive GWAS performed for a tree species. Because of the challenges of establishing, maintaining and phenotyping large tree populations for these types of quantitative genetics studies, the authors cleverly exploit extensive breeding data generated over multiple decades to produce accurate phenotypic estimates. While I applaud the effort to expand GWAS efforts in such difficult to study organisms, the population size used in study is still within orders of magnitude what is necessary to capture a reasonable number of loci that contribute to phenotypic variation. Unsurprisingly, the number of SNPs associated with each trait is quite small.

The manuscript needs extensive and careful editing. Starting with the abstract, writing errors are abundant. For example, in page 3: line 13 ("challenge" instead of "challenging"), line 17 (exome sequencing "of" 5,056 parental trees, not "for"), line 28 (what is "the first time"?). These are just a few instances, and just in the abstract. Errors of this type extend throughout the entire manuscript. Writing should be extensively revised.

Other concerns are outlined below:

Pg.4. Ln. 23-27. I don't think it can be argued that GWAS has supported genomic selection. On the contrary, the power of genomic selection is in avoiding the need to identify specific loci that contribute to complex traits, and rather use all the existing genetic variation to predict phenotypes.

Pg. 4. Ln. 48-52. This does not make sense - why would measure few traits in more individuals limit the statistical power? In GWAS, statistical power is enhanced by larger populations.

Pg. 7. Ln. 16-18. Imputation is difficult in highly diverse species/populations with limited LD. Further explanation about the imputation effort is necessary in the Results section.

Pg. 7. Ln. 36-40. Is there a measure of confidence in this assignment to specific populations of origin? Also, is there a reason why the authors have not used other well-established methods to assign individuals to population of origin, such as Pritchard's Structure?

Pg. 7. Ln. 49. Define dEBV.

Pg. 7. Ln. 55-57. The authors state that growth, phenology, and BB displayed strong correlations with latitude and longitude (for BB), which is expected. A concern in GWAS is that the population structure is so significant that it can not be accounted for by corrective measures (such as using it as a covariate in the model). It is important for the authors to describe here how and why they think they are able to properly account for these effects. Also, I can't find any account of the degree of population genetic differentiation, such as the pairwise Fst observed between all populations.

Pg. 9. Ln. 14. Do they mean r2>0.2?

Pg. 9. Ln. 16-17. In the same lines of the comment above regarding population structure, it is striking that so many more loci were identified as associated with BB, compared to the others. Clearly, BB is a highly heritable trait and likely to produce the most associations. On the other hand, it is also a usually considered a trait that is controlled by fewer loci than, for instance, growth. Thus, how many of these associations may be driven by population structure?

Pg. 9. Ln. 26. Considering the outline of the manuscript (Results before Methods), it may be useful to introduce the readers to the fact that two approaches were used to detect associations, and the reason to apply both methods.

Pg. 10. Ln. 45. The sentence reads as if all the SNPs were validated. I don't think that this is correct or what the authors want to convey. I think that all the SNPs were evaluated, and some were validated in the full-sib progeny.

Pg. 10. Replication of the study. This is an critically relevant component of the manuscript, considering the lack of experimental validation by genetic transformation. However, there is an absence of information regarding the parental composition of these families. Are they part of the unstructured population used in this study? Considering they must be elite genotypes, I assume they most likely are. What is their relationship to the other members of the population? The reason for the need of close scrutiny, is because if there is a close relationship between the parents of the of the full-sib families and the population evaluated in this study, then this analysis does not really validate the results. Moreover, it is relevant to notice that in a full-sib progeny the extent of LD will be much higher than in the population of unrelated trees. Could it be that what is being validated are other loci related to the trait that are in linkage with the associations detected in the original population?

Pg. 11. Identification of candidate genes underlying associated SNPs. While I understand that the authors tried to further dissect the role of these SNPs, this section of the manuscript is highly speculative and deprived of strong scientific evidence. Take for example the transcriptome analysis of genes expressed differentially between early and late burst buds, or at different sampling times and temperature. All of these conditions are known to trigger very large changes in the transcriptome. Thus, it is expected that many genes will be found responding to these treatments. Does that support in any way the relationship between SNPs and genes? Not at all. Furthermore, we have to remember that the SNPs assayed in this study represent largely coding variants - not regulatory variants? IF LD was high, I would consider the possibility that some of the SNPs are in LD with regulatory variants - but LD decays extremely fast. So, I don't think this is the case here.

**Authors Response**

**Point-by-point responses to the reviewers’ comments:**

Reviewer #1:

Manuscript of " Leveraging breeding program and genomic data in Norway spruce (Picea abies L.Karst): a GWAS on adaptive and wood quality traits" (GBIO-D-21-00035) performed GWAS to reveal candidate genes related to 5 traits combining different phenotypic data. This study takes advantages of phenotypic data from different breeding programs, which was quite meaningful, especially for perennial tree study. The results will provide reference for Norway spruce genomic study. But more experiments and analysis should be provided and improved to support the key findings. The conclusion seems too primary. The candidate genes identified were not validated and it is a long way to perform gene editing.

*We have done more experiment and analysis to strengthen the key findings. To further validate the candidate genes detected in our GWAS, we now added an extra dataset that comprises 914 full-sib progeny from 32 families and 49 elite trees/parents, of which 36 are from the whole GWAS population. They were planted in three new field plantations with ca. 9 replicates/ramets per genotype, and individuals were phenotyped for budburst stage (time) and genotyped using a newly developed Norway spruce 50K SNP array (Bernhardsson et al., 2020). This SNP array includes SNPs located inside gene model (exome) and intergenic SNPs. We found that a novel SNP located in the most important gene MA_12842g0020 in this study annotated as MAP3K in Arabidopsis, which is still associated with budburst stage at field age six of the new trials (Table S11).*

*Before the submission of the original manuscript, we had a similar idea as the reviewer to validate the QTL MAP3K using genetic transformation as done in Arapidopsis, crops and poplar, but there is no mature transformation system in any conifer (and gymnosperm) tree species that can be used for genetic transformation in order to verify any gene function. Conifer breeding is also very different from other crops (rice, maize, wheat, etc). Our conifer breeding system is the same as in animal (dairy cattle, sheep, etc) system based on population improvement because genetic transformation is not developed and ready at the moment.*

*Our scientists at UPSC (Umeå Plant Science Centre, Sweden) have made a great effort in the last 25 years to establish a somatic embryogenesis (SE) system for Norway spruce. Even though we are leading in the SE research for conifer transformation, it is still not possible to use SE for genetic transformation. It may still take many years of development to be able to conduct genetic transformation and geneediting. At the moment, we are re-assembling the Norway spruce reference genome and improving the SE system. We are hoping to be able to carry out genetic transformation and gene editing for Norway spruce (and conifer in general) in the next 5 to 10 years.*

*At this moment, we only can follow Human geneticists’ approach to conduct GWAS using a population as large as possible, and use alternative ways to validate our key findings. In this study, we, therefore, tried three alternative ways to validate the findings. First, we performed cross-validation in a large field plantation growing at a different climate condition for several decades (ca. 30 years in this study). Second, we performed RNA-seq among early budburst and late budburst trees sampled in the field experiment (common) garden. To further verify the key finding as suggested, we performed crossvalidation using a new genotype data (50K SNP array data from 914 genotypes) for budburst stage at field age six from three southern Sweden populations.*

*Since we are not able to conduct genetic transformation and gene editing for any conifer species (and Gymnosperm species, in contrast to Angiosperm species) for a perceivable time, uur use of verified candidate gene is to increase the efficiency of genomic selection in the breeding program as is done in animal breeding (Meuwissen and Goddard. 2010).*

*One of the main objectives of this study is to set an example for effective GWAS in tree species (particularly conifer). That is to use all unrelated trees from as many breeding populations as possible. So far, most GWASs in conifers use a small population (usually less than 500 unrelated trees) planted in a single plantation at the same time. Here we showed that all unrelated trees in a national breeding program that spanned more than a half-century can be used for a more comprehensive GWAS. Also, our population size is still small comparing to the GWAS in human being, but we were able to use all trees selected in the last 60 years (10 times bigger than the usual population in conifer GWAS studies). We hope this will inspire other tree breeding programs to follow our example to substantially increase future population size in GWAS in tree species.*

The comments are listed below:

1. Although the limitation of reference genome for Norway spruce, too little SNPs were identified using exon-sequencing for more than 4000 individuals. Is it possible to get much more genotypes? Such as low depth sequencing. If so, the GWAS result would be much more reliable. Because many traits were not directly associated SNPs in exon.

*Yes, we agree that more SNPs would have been discovered, if we used whole-genome resequencing with low depth sequencing, such as 3X coverage. We also agree that GWAS result would be much more reliable using this type of data, particularly for large (>20G) and heterogeneous genome such as Norway spruce. SNPs from intergenic regions, such as promoters and enhancers will be associated with traits. So far we have only re-sequenced and analysed 30 trees. The cost of re-sequencing many Norway spruce trees is prohibitive. Currently, even if we were to only use 3X coverage, each sample will cost ca. US$ 521 (4,500 SEK) because of the large and highly heterozygous spruce genome. Thus, it would be too expensive to do GWAS in a medium-size population using re-sequencing (for example of 1000 trees, costing US$ 521,000) at the moment.*

2. Why the author used maf value 0.03?

*Based on previous simulation (Li et al., 2011), when more than 2000 individuals are genotyped and with an average coverage of 15x, SNP discovery with maf value of more than 3% could be ~100% detected (Figure 1 in the Li et al. 2011). Thus, we selected maf value 0.03.*

3. In GWAS, two models were used (BLINK and CMLM), why selected these two? As we know, the CMLM would ignore much more related SNPs, and only kept the most significant signal, which might reduce the number or important loci that might be related to trait.

*First, we wanted to only use the most advanced model to detect SNPs in our GWAS. BLINK was shown to be able to find more SNPs compared to most of the single-locus models described by Huang et al. (2018). Second, we also wanted to test the detected loci in subpopulations. However, BLINKwill ignores more* *related SNPs, and only keep the most significant signal. Thus, we performed GWAS using both methods, as stated in the previous version, Page 26, lines 54-58. Based on another reviewer’s suggestion, we now have moved it into results.*

4. Why the author compare the results of BLINK and CMLM? Do you want to select a much better model?

*Based on the literature (Huang et al., 2018), multi-locus BLINK has a stronger power to detect SNPs with relatively smaller effect than other single-locus models, such as CMLM, fixed and random model circulating probability unification (FarmCPU) method (Liu et al., 2016), and single marker genomewide efficient mixed-model association (GEMMA) (Zhou & Stephens, 2012). But the BLINK will exclude those SNPs that are in strong LD with the most significant SNP. However, we wanted to test the replication of the associated locus in each genetic cluster, thus, we also used the CMLM method.*

5. Actually, the authors performed GWAS using different population or clusters and different models to idenfify QTLs. But how to make sure which results were much accurate. Too many ways would increase the false positives. How to deal with such problem?

*Statistically, it will indeed increase the number of false positives. On the other hand, GWAS performed within subpopulation could be more reliable since the effect of population structure will be less. One of the main purposes of performing GWAS in subpopulations is to validate the QTLs and detect the repeatability of QTL detected in the whole population and subpopulations. Thus, we will not try to use a smaller p-value in this GWAS. We specified when QTLs were only detected from one of the subpopulations in the results.*

6. Page10 line 33, what is the possible reason for the higher phenotypic variation of BB.

*Budburst stage (BB) has a higher heritability than growth and wood quality traits and this means that BB has higher genetic variation relative to environmental control in comparison with other traits. This is one of the reasons that we detect several independent SNPs with large effects for budburst phenotypic variation.*

7. Page10 line 45, why delete FD and DBH?

*For these two traits, we don’t have phenotypic data in many sites. First, for frost damage (FD), only the field plantations that experienced strong frost events can be used to collect data for frost damage. Second, in the Northern Swedish breeding populations, DBH was not usually measured in the field plantation even if it is a target trait in the breeding, and height was used as a correlated (surrogate) trait for DHB as the genetic correlation between height and DBH usually was ca. 0.9. Thus, we don’t have the data from the two northern Swedish field plantations.*

8. In GWAS, what is the threshold of P-value, how do you determine it?

*We used a false discovery rate (FDR) <5% as the threshold for each GWAS model. The criteria are slightly lower than that based on the Bonferroni correction 0.05/number of loci. In Table S7 (revised version), we presented that the largest p-value for associated SNPs is 8.74*10-6.*

9. There are SNPs in gene intron region, such as page 11 line 33-40. Such SNPs can not change gene function, so how they affect the phenotypes? Do they affect the gene expression? Much more experiments should be carried out.

*It would have been more intuitive if our GWAS hit is located inside the gene body that affects proteincoding. However, a large proportion of the GWAS hits identified in human, animals and plants were found in the intronic or intergenic region (Bartonicek et al., 2017). One possible explanation is that the detected SNPs were not the causal variants themselves, instead they tag the variants by linkage disequilibrium (LD). Another possibility is that they were regulatory variants that mediate the transcripts level, as has been demonstrated in other studies (Schierding et al., 2016). We should state that what we report here may not be the actual functional variants and future experiments are required to validate the results.*

10. Page 11 Line 45, the word "inside gene", which region? Should be declared clearly.

*We have revised it.*

11. When selecting candidate genes based on significant SNPs, have you considered the LD? Or just selected the gene containing SNPs. As we know, many traits were not caused by a single SNP, but large genome variations.

*Yes, we considered the LD as described as in page 28, Line 4-16 in the previous manuscript. Most of the selected genes (52 of the total 57 genes) contained SNPs. A few genes (7 genes of the total 57 genes) closed to SNPs associated SNPs within a contig also could be selected.*

**Reviewer #2:**

This manuscript reports the most extensive GWAS performed for a tree species. Because of the challenges of establishing, maintaining and phenotyping large tree populations for these types of quantitative genetics studies, the authors cleverly exploit extensive breeding data generated over multiple decades to produce accurate phenotypic estimates. While I applaud the effort to expand GWAS efforts in such difficult to study organisms, the population size used in study is still within orders of magnitude what is necessary to capture a reasonable number of loci that contribute to phenotypic variation. Unsurprisingly, the number of SNPs associated with each trait is quite small.

The manuscript needs extensive and careful editing. Starting with the abstract, writing errors are abundant. For example, in page 3: line 13 ("challenge" instead of "challenging"), line 17 (exome sequencing "of" 5,056 parental trees, not "for"), line 28 (what is "the first time"?). These are just a few instances, and just in the abstract. Errors of this type extend throughout the entire manuscript. Writing should be extensively revised.

*Dr Chris Harwood from CSIRO edited the whole manuscript.*

Other concerns are outlined below:

Pg.4. Ln. 23-27. I don't think it can be argued that GWAS has supported genomic selection. On the contrary, the power of genomic selection is in avoiding the need to identify specific loci that contribute to complex traits, and rather use all the existing genetic variation to predict phenotypes.

*Yes, the power of genomic selection is in avoiding the need to identify specific loci that contribute to complex traits. But on the other hand, if just including all QTLs in genotypic data or including most significant QTLs in the genomic data rather than just random SNPs could improve the GBLUP model (Lenz et al., 2017; Chen et al., 2018; Tan & Ingvarsson, 2019). Especially, if we include the SNPs with large effects as a covariate in genomic prediction, gain could be improved by 10% or even more based on the literature (Bernardo, 2014; Sarinelli et al., 2019).*

Pg. 4. Ln. 48-52. This does not make sense - why would measure few traits in more individuals limit the statistical power? In GWAS, statistical power is enhanced by larger populations.

*Yes, statistical power could be enhanced by larger populations. But if we consider the multivariate model, more traits with reasonable genetic correlations (not the completed correlation or no correlation) will improve the power of the model.*

Pg. 7. Ln. 16-18. Imputation is difficult in highly diverse species/populations with limited LD. Further explanation about the imputation effort is necessary in the Results section.

*Yes, it is difficult for imputation with limited LD if it is based on GBS data. If it is based on exome capture data, which here ca. 6 SNPs within 240 bp (up/downstream 120 bp) for each probe (Vidalis et al., 2018) and 15x coverage, it is still quite good using Beagle for imputation as described in results and Figure S1.*

Pg. 7. Ln. 36-40. Is there a measure of confidence in this assignment to specific populations of origin? Also, is there a reason why the authors have not used other well-established methods to assign individuals to population of origin, such as Pritchard's Structure?

*In this study, we used Admixture to ascertain the best number of populations. However, the detailed results (Figure below) were not included in the submitted version because we thought that is not a population genomics manuscript. Like the program STRUCTURE (Pritchard et al. 2000), ADMIXTURE is model-based and classifies individuals into K ancestral clusters, allowing individuals to have an admixed origin. ADMIXTURE was used with ten-fold cross-validation and 200 bootstraps. The K-value corresponding to the lowest cross-validation error was considered as the “best” one, although identifying the “true” number of clusters remains an elusive problem and, as stressed by many authors, the number of clusters should be treated carefully (Pritchard et al. 2000; Bradburd et al. 2018). In a previous study using a subset of southern populations (Chen, J et al., 2019), fivefold crossvalidation using Random Forest showed that this method was robust (error rate <8%).*

*Figure: Admixture analyses describing the proportion of ancestral components for K=11, Yellow and dark red represent P. omorika and P. obovate genetic background, respectively, while other colours represent P. abies. Dodger blue and violet present Romania (ROM) and Central European (CEU) domains, respectively. Black represents the Fennoscandian (NEF) domain. Dark orange may represent in southern Sweden domain (CSE). Green yellow and forest green represent parts of central Sweden (CSE). Cyan and midnight blue might present two Alpine domains (ALP), separately. Blue represents the ALP domain. To overview the complicated NFE and CSE populations, we sorted them by K information again (B). More than half of NFE population, the part of the ancestral component is from Northern Finish or Rus-Baltic group. And also maybe there is a solo group with green colour in the northern population without Rus-Baltic group information, and also there may be a solo group in central and southern Sweden (orange).*

Pg. 7. Ln. 49. Define dEBV.

*We defined it.*

Pg. 7. Ln. 55-57. The authors state that growth, phenology, and BB displayed strong correlations with latitude and longitude (for BB), which is expected. A concern in GWAS is that the population structure is so significant that it can not be accounted for by corrective measures (such as using it as a covariate in the model). It is important for the authors to describe here how and why they think they are able to properly account for these effects. Also, I can't find any account of the degree of population genetic differentiation, such as the pairwise Fst observed between all populations.

*Our population is indeed heavily structured, represented by proportions of individuals belonging to the same subpopulations, commonly known as the Q matrix (Thornsberry et al., 2001; Larsson et al., 2013). One way to account for population structure is to compute the Q matrix using ADMIXTURE or PCA derived from genome-wide genetic markers (Reich et al., 2008; McVean, 2009) and fit it as a fixed effect in the model. Statistically, this is equivalent to fitting all the markers in the model (known as SNP-BLUP model) and further equivalent to fitting one random effect using the Kinship matrix derived from genome-wide genetic markers as covariance structure (Known as G-BLUP) (Yang et al., 2010). Here we used the GBLUP model implemented in CLMM and an SNP-BLUP like model implemented in BLINK to account for relationship, also we tested adding one to several principal components in both CMLM and BLINK to test the effects of population structure. Population structure and relationship usually lead to inflation for the test statistics measured using inflation factor (regression slope for nulltest statistics vs. real test statistics). Applying CMLM without fitting the PC as a covariate, inflation factors were all within the range of 0.95 to 1.05 indicating there was no confounding with population structure. Applying BLINK without fitting the PC as a covariate, we found the inflation factors for all the populations and traits in this study are within 1.0 ± 0.05, except for the genetic cluster ROM with small population size (n=30) and the trait BB for the whole population (IF=1.09).*

*I have added this part of the result in Table S4 and the discussion.*

Pg. 9. Ln. 14. Do they mean r2>0.2?

*Yes. We changed it.*

Pg. 9. Ln. 16-17. In the same lines of the comment above regarding population structure, it is striking that so many more loci were identified as associated with BB, compared to the others. Clearly, BB is a highly heritable trait and likely to produce the most associations. On the other hand, it is also a usually considered a trait that is controlled by fewer loci than, for instance, growth. Thus, how many of these associations may be driven by population structure?

*One of the reasons is that several large effects SNPs were detected in both BLINK and CLMM. Multiloci BLINK could have more power to detect the small effects SNPs when a few large effects SNPs were fixed in the model because residuals of the model are becoming smaller. If those loci are not replicated in each such population, it is possible that they are driven by population structure.*

Pg. 9. Ln. 26. Considering the outline of the manuscript (Results before Methods), it may be useful to introduce the readers to the fact that two approaches were used to detect associations, and the reason to apply both methods.

*Yes, it is a good suggestion. We have moved the reasons in Pg. 28, Ln 48-58 in the previous version to results.*

Pg. 10. Ln. 45. The sentence reads as if all the SNPs were validated. I don't think that this is correct or what the authors want to convey. I think that all the SNPs were evaluated, and some were validated in the full-sib progeny.

*Yes, you are right. We correct it.*

Pg. 10. Replication of the study. This is an critically relevant component of the manuscript, considering the lack of experimental validation by genetic transformation. However, there is an absence of information regarding the parental composition of these families. Are they part of the unstructured population used in this study? Considering they must be elite genotypes, I assume they most likely are. What is their relationship to the other members of the population? The reason for the need of close scrutiny, is because if there is a close relationship between the parents of the of the full-sib families and the population evaluated in this study, then this analysis does not really validate the results. Moreover, it is relevant to notice that in a full-sib progeny the extent of LD will be much higher than in the population of unrelated trees. Could it be that what is being validated are other loci related to the trait that are in linkage with the associations detected in the original population?

*We knew the parental composition as 51 of the total 55 parents were genotyped and are a subset of the Fennoscandia group (NFE) in this GWAS (Chen et al., 2018; Chen, Z-Q et al., 2019). In this study, ca. 5000 elite trees are P0 generation, ca. 60 individuals are F1 generation from crosses from P0 generation. Ideally, P0 elite trees should be unrelated based on the criteria of phenotypic selection (controlling inbreeding depression in Norway spruce breeding population). In the revised version, we added a boxplot of relationships between the 51 parents (5% of the total NFE population with 1041 elite trees) and other 990 northern elite trees (supplementary Figure S13 and also below). From Figure S13, we could see 16 elite trees (relationship value r>0.2) could be related to 51 elite trees/parents due to maybe some mistakes, such as sampling errors, labelling error or errors in lab work.*

*Yes, the candidate SNPs that are being validated may be other loci related to the trait that is in linkage with the associations detected in the original population. However, considering the large variation such as microenvironmental variation within a field plantation (ca.1 hectare) and also the climate variation within 28 years for some traits in the field, even if LD is high in the full-sib progeny, we think it is still strong evidence for validation.*

Pg. 11. Identification of candidate genes underlying associated SNPs. While I understand that the authors tried to further dissect the role of these SNPs, this section of the manuscript is highly speculative and deprived of strong scientific evidence. Take for example the transcriptome analysis of genes expressed differentially between early and late burst buds, or at different sampling times and temperature. All of these conditions are known to trigger very large changes in the transcriptome. Thus, it is expected that many genes will be found responding to these treatments. Does that support in any way the relationship between SNPs and genes? Not at all. Furthermore, we have to remember that the SNPs assayed in this study represent largely coding variants - not regulatory variants? IF LD was high, I would consider the possibility that some of the SNPs are in LD with regulatory variants - but LD decays extremely fast. So, I don't think this is the case here.

*We agreed with the reviewer’s opinion that transcriptome analyses are not direct evidence to support the relationship between SNPs and genes. This is why we used another, but related population to further support the observation. We also agree that the exome represents largely coding variants. The good news is that now we have a new SNP array to including a large number of SNPs (23,381SNPs) from intergenic in our new genotyping population to further support the results. LD is very low in Norway spruce as in other conifers as suggested.*

References:

*Bartonicek N, Clark MB, Quek XC, Torpy JR, Pritchard AL, Maag JLV, Gloss BS, Crawford J, Taft RJ, Hayward NK, et al. 2017. Intergenic disease-associated regions are abundant in novel transcripts. Genome Biology 18(1): 241.*

*Bernardo R. 2014. Genomewide selection when major genes are known. Crop Science 54(1): 68- 75.*

*Bernhardsson C, Zan Y, Chen Z, Ingvarsson PK, Wu HX. 2020. Development of a highly efficient 50K SNP genotyping array for the large and complex genome of Norway spruce (Picea abies L. Karst) by whole genome re‐sequencing and its transferability to other spruce species. Molecular Ecology Resources.*

*Chen J, Milesi P, Jansson G, Berlin M, Karlsson B, Aleksic J, Vendramin GG, Lascoux M. 2019. Genomic data provides new insights on the demographic history and the extent of recent material transfers in Norway spruce. Evolutionary Applications(12): 1539–1551.*

*Chen Z-Q, Baison J, Pan J, Karlsson B, Andersson B, Westin J, García-Gil MR, Wu HX. 2018. Accuracy of genomic selection for growth and wood quality traits in two controlpollinated progeny trials using exome capture as the genotyping platform in Norway spruce. Bmc Genomics 19(1): 946.*

*Chen Z-Q, Baison J, Pan J, Westin J, Garcia-Gil MR, Wu HX. 2019. Increased prediction ability in Norway spruce trials using a marker x environment interaction and non-additive genomic selection model. Journal of Heredity 110(7): 830–843.*

*Huang M, Liu X, Zhou Y, Summers RM, Zhang Z. 2018. BLINK: a package for the next level of genome-wide association studies with both individuals and markers in the millions. Gigascience 8(2): 1–12.*

*Larsson SJ, Lipka AE, Buckler ES. 2013. Lessons from Dwarf8 on the strengths and weaknesses of structured association mapping. PLOS Genetics 9(2): e1003246.*

*Lenz PRN, Beaulieu J, Mansfield SD, Clément S, Desponts M, Bousquet J. 2017. Factors affecting the accuracy of genomic selection for growth and wood quality traits in an advanced-breeding population of black spruce (Picea mariana). Bmc Genomics 18(1): 335.*

*Li Y, Sidore C, Kang HM, Boehnke M, Abecasis GR. 2011. Low-coverage sequencing: implications for design of complex trait association studies. Genome Research.*

*Liu X, Huang M, Fan B, Buckler ES, Zhang Z. 2016. Iterative usage of fixed and random effect models for powerful and efficient genome-wide association studies. PLOS Genetics 12(2): e1005767.*

*McVean G. 2009. A genealogical interpretation of principal components analysis. PLOS Genetics 5(10): e1000686.*

*Reich D, Price AL, Patterson N. 2008. Principal component analysis of genetic data. Nature Genetics 40(5): 491-492.*

*Sarinelli JM, Murphy JP, Tyagi P, Holland JB, Johnson JW, Mergoum M, Mason RE, Babar A, Harrison S, Sutton R, et al. 2019. Training population selection and use of fixed effects to optimize genomic predictions in a historical USA winter wheat panel. Theoretical and Applied Genetics 132(4): 1247-1261.*

*Schierding W, Antony J, Cutfield WS, Horsfield JA, O’Sullivan JM. 2016. Intergenic GWAS SNPs are key components of the spatial and regulatory network for human growth. Human molecular genetics 25(15): 3372-3382.*

*Tan B, Ingvarsson PK. 2019. Integrating genome-wide association mapping of additive and dominance genetic effects to improve genomic prediction accuracy in Eucalyptus. bioRxiv: 841049.*

*Thornsberry JM, Goodman MM, Doebley J, Kresovich S, Nielsen D, Buckler ES. 2001. Dwarf8 polymorphisms associate with variation in flowering time. Nature Genetics 28(3): 286- 289.*

*Vidalis A, Scofield DG, Neves LG, Bernhardsson C, García-Gil MR, Ingvarsson P. 2018. Design and evaluation of a large sequence-capture probe set and associated SNPs for diploid and haploid samples of Norway spruce (Picea abies). bioRxiv.*

*Yang J, Benyamin B, McEvoy BP, Gordon S, Henders AK, Nyholt DR, Madden PA, Heath AC, Martin NG, Montgomery GW, et al. 2010. Common SNPs explain a large proportion of the heritability for human height. Nature Genetics 42(7): 565-U131.*

*Zhou X, Stephens M. 2012. Genome-wide efficient mixed-model analysis for association studies. Nature Genetics 44(7): 821–824.*

**Second round of review**

**Reviewer 1**

Although the studies on tree species were meaningful, the authors still did not perform experimental validation of candidate genes identified in GWAS, which was greatly important to enrich the results. Although the transformation system was not available, they should take appropriate model species to validate it. The authors have been following the Human researches to perform GWAS using large number of individuals, while they used quite low-density SNPs in this study. For human genetic study, the transformation was also not performed in human, but they used other animals, such as Mus musculus. The authors explained that the low-depth re-sequencing would cost a lot. But the results of this manuscript would only be reliable under any of such two conditions, full experimental validation or high-density SNPs. Taking these two aspects, the results in this study might be not reliable for other researchers.

**Reviewer 2**

The authors have provided a satisfactory reply to all my questions and concerns. The study has limitations, but they are primarily a consequence of the system under study. As the authors point out, the study is a strong demonstration of the use of large breeding populations studies over many decades.

**Authors Response**

**Point-by-point responses to the reviewers’ comments:**

Reviewer #1:

Although the studies on tree species were meaningful, the authors still did not perform experimental validation of candidate genes identified in GWAS, which was greatly important to enrich the results. Although the transformation system was not available, they should take appropriate model species to validate it. The authors have been following the Human researches to perform GWAS using large number of individuals, while they used quite low-density SNPs in this study. For human genetic study, the transformation was also not performed in human, but they used other animals, such as Mus musculus. The authors explained that the low-depth re-sequencing would cost a lot. But the results of this manuscript would only be reliable under any of such two conditions, full experimental validation or high-density SNPs. Taking these two aspects, the results in this study might be not reliable for other researchers.

*We have directly validated the most important gene by using three large datasets including two large populations from 5 field plantations located across Sweden and also by using RNA-seq data.*

*We agree with the reviewer that full experimental validation is an important step to support a GWAS results. Thus we would like to validate it in Norway spruce in the future when the genome assembly is well done and the gene transformation system becomes mature.*
